# Supplementary material for: The experience of living with human immunodeficiency virus among adolescents at Felege Hiwot Comprehensive Specialized Hospital Bahir-Dar, Northwest Ethiopia, A phenomenological study
Source: PLoS One. 2025 Jan 9;20(1):e0308347. doi: 10.1371/journal.pone.0308347 (PMC11717265; doi:10.1371/journal.pone.0308347)
Supplement: S1 Annex — (DOCX) [file pone.0308347.s001.docx]

**Supplementary information**

**Annex: Semi structured open ended interview guiding questions in English**

**Part I: Semi-structured interview guide questions for adolescents living with HIV/AIDS**

**1. Back ground data of participants**

- Probe: Demography (age, sex, educational level, occupation, marital Status)

2. What does living with HIV means to you? How you are living with HIV

Probe

- How do you express living with HIV?
- How do you feel when you think about your HIV status?
- Are there any challenges you have faced or experiencing currently?
- How do you see the burden of HIV/AIDS on your health, and life

3. How do you know yourself about you are living with HIV?

Probe

- Who told you, when, where, what were the conditions?
- How did you feel when you found out about your status?
- Have you disclose yourself for others(family, peers and any individuals)
- For those to whom you have told, what were your measures for picking those people?
- What were the conditions? How did the individual react to this news?
- Have you experienced stigma and discrimination after disclosure of yourself?
- For those not unveiled to your status, for what reason didn't you reveal to them?

4. What is your intention to have a boyfriend/girlfriend or Husband/ wife currently or in the future?

Probe

- Is he/she aware of your HIV status? Are you in a sexual relationship;
- Are you rehearsing safe sex?
- What do you think about HIV transmission and avoidance strategies?
- What sort of husband/wife do you need in the future, their HIV status?

5. How you are taking ART medication?

- - On habit and challenges of taking ART?
  - About pill-burden, fear of taking ART in front of other people, side effect, and facility related issues?

6. Is there anything you want to add?

7. Thank you very much! For your participation.
