# Supplementary material for: The experience of living with human immunodeficiency virus among adolescents at Felege Hiwot Comprehensive Specialized Hospital Bahir-Dar, Northwest Ethiopia, A phenomenological study
Source: PLoS One. 2025 Jan 9;20(1):e0308347. doi: 10.1371/journal.pone.0308347 (PMC11717265; doi:10.1371/journal.pone.0308347)
Supplement: S1 File — (DOCX) [file pone.0308347.s002.docx]

ተሳታፊ አንድ፡

**ከኤች አይቪ ቨይረስ ጋር አብሮ መኖር ማለት ለአንቺ ምን ማለት ነዉ**

መላሽ፡ ከኤች አይቪ ቨይረስ ጋር አብሮ መኖር ማለት ለኔ እንደማንኛዉም ሰዉ በልቶ ጠጥቶ ተጨዉቶ መኖር ነዉ ግን ይህ የሚሆነዉ ግን መድኃኒታችንን በአግባቡ ስንወስድ ነዉ፡፡ አሁን ለምሳሌ ሳይመረመሩ ብዙ ኤች አይቪ ቨይረስ ያለባቸዉ ሰዎች አሉ፡፡ በመጀመሪያዎች አካባቢ በ1980ዎቹ ኤች አይቪ እንደመጣ ሰዉ ይገለል ነበር፤ መድኃኒትን ሰይወስድ ይሞት ነበር አንድ እግሩ ይቆረጥ ነበር አልጋ ለይ ነበር ሚተኘዉ አሁን ለይ ያለዉ ሁኔታ ግን ሁሉም ሰዉ በይባልም በአግባቡ ይመገባል መድኃኒትም ይወስደል፡፡ የተመጠጠነ ምግብ ይወስዳል፤ እኔም ጭምር መድኃኒቱንም በአግባቡ እወስደለሁ፡፡ ምግብም እበላለሁ፤ ፆምም እፆማለሁ፡፡ ችግር የለብኝም ምክኒያቱም ማታ ስለምወስደዉ

**ከቨይረሱ ጋር እየኖርሽ እንደሆነ ስታስተዉሺ ምን ይሰመሻል**

ቨይረሱ የተገኘብኝ ሶስት ዓመቴ ጀምሮ ነዉ ያወቅሁት ግን አምስተኛ ክፍል አካባቢ እያለሁ ነዉ፡፡ እኔ ግን ሚሰማኝ ነገር የለም ግን ለወንድሞቼና ለእህቶቼለይ ይህ ነገር እታፈንታ እንዲደርሰበቸዉ አልፈልግም፡፡ በዚህ ጉደይ ደሞ እናት አባት ታለቅ ታናሽ መካከል ለይ ያሉት ለዚህ ነገር ግዴታ ለእነሱ /ለተናነሾቹ ስለሚያስፈልጉ በእነሱ ብቻ ሳይሆን እነሱ ጋር አብሮ ሚሄድ ስለሚያሰፈልገቸዉ ይህንን ነገር ቢከታተሉ ጥሩ ነዉ፡፡ ለምሳሌ ሴቶች በእርግዝና ወቅት መከታተል አለባቸዉ፤ ወንዶችም አብሮ ሂደዉ መማርመር የሚለዉን ነገር ማደበር አለበት በማንኛዉም ቦታ ምርመራ መሰጠት አለበት፡፡ያን መማርመርን ትደግፊያለሽ ማለት ነዉ አዎ እደግፋለሁ፡፡

ያገጠመሽ ነገር/ፈተና ሁኖብኛል ምትይዉ ነገር አለሽ

ያገጠመኝ ነገር የለም ያጋጠሙ ፈተና የምለዉ ነገር የለም፤ ግን አንድ ጊዜ የሆነ ሰዓት ለይ በሆነ ምክኒያት መደሃኒት አቆርጬ ነበር እና ያማል፡፡ የራስ ምታት የሕመም ስሜት አለዉ ግን ቆይቶል የዛሬ 5/6 አመት አካባቢ ነዉ እናም መድሃኒት መቆራረት ያሰምማል/ራስ ህመም አለዉ ሰዓት መሸራረፍ ራሱ በጣም ከባድ ህመም አለዉ

መድሃኒት አቆርጠሽ የነበርሽበት በምን ምክኒያት ነዉ

የሆነ ቦታ ሄጄ ነበር እና ስሄድ መድሃኒቱን ረስቼዉ ሂጄ ለአንድ ሳምንት ቆይቼ ተመልሼ ስወስድ ራሴን የማመም ስሜት ተሰመኝ ከዛ በሆላ መድሃኒቱን አቆርቼ አለዉቅም

በቨይረሱ ምክኒያት የደረሰብሽጫና /ተፅኖ አለሽ

እስካሁን ያገጠመኝ ነገር የለም /የወደፊት ይታያል ነዉ እንጂ

እህቴም የእህቴ ባልም አባቴም ይደግፉኛል፡፡

ቨይረሱ እንደላብሽ ማን ነገረሽ/እንዴት አወቅሽ

የሆነ ሰዓት ለይ ህፃን ሁኜ ፕላምፒ ኔትና ሽሮፕ ነበር ምወስደዉ እና በጣም ነበር የምጠላዉ እና ፕላምፒ ኔት ሲሰጠኝ እናቴን እምቢ ነበር የምላት እየኮየሁ እየኮየሁ እናቴ ታመመች እና እዚህ ምታመጠኝ አክስቴ ነበረች እንደዚህ ኤች አይቪ ነዉ ያለብሽ ብላ ነገረችኝ /5ኛ ክፍል እያለሁ ያን ጊዜ ምንም አልተሰማኝም፡፡ መድኃኒቱን ምትወስጂዉ እንዳትሞቺ ነዉ ያዉ ህፃንነት ጅልነት ነዉና ምንም አልተሰማኝም/ምንም ሁኔታ አልነበረኝም፡፡ ይህ ነገር አሁን ለይ ቢነገረኝ ኑሮ እኔ እንጃ የሆነ ስሜት ነበር ሚሰማኝ/በጣም ነበር የምጎደዉ እኮንም በህፃንነት ተነገረኝ/አወቁ ስለዚህ በልጅነቴ ሰለአወቁ ደስተኛ ነኝ፡፡ አደጌ/ተልቅ ሁኜ ያንን ነገር በዉቀዉ በስነ-ልቦነ እጎዳ ነበር፡፡ አሁን ለይ በዉቀዉ እኔ ብቻ ነበር ሚመስለኝ

ለወደፊቱ ለህፃነት በህፃንነት እድሜ ቢነገረቸዉ አሪፍ ነዉ ትያለሽ

አዎ፤ በልጅነታቸዉ ቢነገረቸዉ ይሻለል ምክኒያቱም ህፃናት ንፁህ ወረቀት ናቸዉ እና ምንም ነገር ስለመያዉቁ ይህንን ነገር በ9/10 ዓመቱ ቢነገረዉትክከለኛዉ እድሜ ነዉ በዚህ እድሜ ስለጨዋታ ነዉ እንጂ ትኩረቱን ሚያደርገዉ ስለመድሃኒቱ አያዉቅ ስለቨይረሱ አያዉቅ ግን አሁን ሳይገቡ መማርመር ጥሩ ነዉ እንደላበት ከወቀችም ለልጆ ጣቃሚ ነገር ነዉ

ሲነገርሽ ስሜተሸ ለይ ለወጥ ነበረሽ/ምንም አይነት የስሜት ለወጥ አልነበረኝም፡፡

ራስሽን ለሌላ አሰዉቀሻል/አዎ ቤተሰብ ያዉቃል ለምሳሌ አክስት/የአክስቴ ባል ያዉቃሉ እነዲሁም የቅርብ/ምርጥ/ ጎደኛዬ አለችኝ ነግሬታለሁ መድሃኒት እንድወስድ ታግዛኛለች/አንቺ ምን ሁነሽ ነዉ መድሃኒት ዉሰጂ ትላለች፡፡ ይህ ምንም ማለት አደለም እኔም የነርስ ልጅ ስለሆንኩ አዉቀዋለሁ አለችኝ፤ እንዴት መረጥሺያት የ4ዓመት ጎደኛዬ ስለሆነች

ስትነግሪት ምን አይነት ሁኔታዎች ነበሩ/ ከነገርኮት በሆለ ለኔ በጣም ነዉ የቀለለኝ ይህንን ነገር ደብቄያት ሰለቆየሁ ከብዶኝ ነበር ከነገርሆት በሆለ በጣም ቀለለኝ፡፡ ምንም ማለት አይደለም እንደዚህ ብዪ እንደዚህ አርጊ እያለች ትመክረኛለች እንዲሁም ከነገርሆት በሆለ ግነኙነታችን ጨምሮል፡፡ ለሌሎችስ ለምን አልነገርሺያቸዉም /ምክኒያቱም አሁንም መገለልና መድሎ አልቀረም፡፡

ት/ቤት ለይ ቨይረሱ እያለበት ያለበት ማያስመስል ሰዉ ስላለ እንዲሁም በዚህ ፕሮግረም /OTZ/ ለይም አይሳተፍም ነዉ እንጂ ማህበረሰቡ 5% ቢሆን ነዉ ከቨይረሱ ነፃ ሁኖ ሚኖረዉ ማለትም አብዛኛዉ ሰዉ በቨይረስ ተይዞልምክኒያቱም ሰዉ በአግባቡ 1ለ1 መወሰን መታቀብ የሚለዉን ስለማይተገብር እንደፈለገዉ/በፈለገዉ መንገድ ስለሚሄድ አሁን እህቴ የለባትም እኔና አባቴ ብቻ ነን ያለብን እናቴ ሙታለች፡፡

ጎደኛዬ ከነገርኮት በሆለ ይበልጥ እቀረበች ነዉ የሄደችዉ እንጂ አልሸሸችም የነርስ ልጅም ስለሆነች ታዉቃወለች ከእሶ መገለል የሚል ነገር የለም፡፡ ሌላ ሰዉ የሰማ ስለሌላ ምንም መገለል አልደረሰብኝም፡፡

ቨይረሱ በት/ት ለይ ያደረሰብሽ ጨና/ተፅዕኖ /በት/ት ለይ የለም ግን OTZ ፕሮግራመ ሳይጀመር ት/ት ለመከታተል ያስቸግር ነበር ማለትም መድሃኒት ለመዉሰድ እኔ ከት/ቤት መቅረት ወይም እህቴ ታመጣልኛለች አሊያ ኖት ያልፋኛል አሁን ለይ ይህ ነገር ቀርቶል፡፡ መድሃኒት ምንወስደዉ ቅዳሜ እስከ ስድስት ሰዓት በለዉ ነዉ፡፡ ሌላዉ ፆም በጣም ነበር ምወደዉ ና መድሃኒቱ ጠዋትና ማታ ስለሚሆን አባቴ እንድፆም አይፈቅድልኝም ቁርስ በልቼ በፊቱ እያዬኝ መድሃኒት ወስጄ ትምህርት ቤት እንድሄድ ነበር ሚፈልግ እና አሁን ለይ በቀን አንዴ ስለምወስድ እንደ ድሮ በቀን ሁለቴ ስለማልወስድ አሁን ለይ ምንመ ተፅዕኖ የለም፡፡ከፆም በሆለ በልቼ ስለምወስድ ችግር የለም

በባህርይ ለይ ያሰደረብሽ ተፅዕኖ አለ/ የለም በተፈጥሮ መነጫነጭ ቁጡነት የራሴ በህርይ /ፀበይ ነዉ ሚያስቀይም ሰዉም ካለ ፊት ለፊት እዘዉ ነዉ ምነገር ይህንን የመድሃኒት ነዉ ብዬ አለስብም

የወንድ ጎደኛ /ፍቅረኛ አለሽ/የለኝም ለወደፊትስ ምን ሀሳብ አለሽ/ለወደፊቱ ፈጣሪ ያውቃል፡፡ለወደፊቱ ማይቀር ጉደይ ነዉ

ግን ቢኖርም እንኮ ኔጋቲቭ የሆነ ሰዉ መያዝ አልችልም ይከብዳል ነገሩ ቢኖረኝም እንኮ ተመሳሰይ የሆነ ሰዉ ቢሆን እመርጣለሁ የሆነ ነገር ብትለዉ ለይቀበል ይችላል ግን ሚወደኝ ከሆነ እስከመጨረሻዉ ይቆያል ይህ ነገር ላንቺ ነዉ ለኔ ምንም አይደላም ሊል ይችላል ፖዘቲቭ ከሆነ ሰዉ ጋር ቢሆን የበለጠ ትቀርበዋለህ ፣ትነጋገራለህ፣ለልጆች ታስበለህ እኔ ፖኣቲቭ ሁኜ እሱ ኔጋቲቭ ከሆነ ግን የማይወደኝ ከሆነ ጥሎኝ ነዉ ሚሄድ ግን በትክክል ሚወደኝ ከሆነ ግን አብሮኝ ይቆያል ፣ጥሎኝ ከሄደ ይህ ደሞ ለሴት ልጅ በጣም ከበድ ነዉ ፣፣ ይህንን ለመወሰን ጊዜ ያስፈልጋል

ስለ ኤች አይቪ ስርጭት አሁ ለይ ስርጭቱ ይቀንስ አይቀንስ በላዉቅም ግን እንደ እኛ እዚህ ፈለገ ህይወት እንደሚሰጠዉ ኦቴዝ ሌሎች ለይም ቢሰጥ እነሱ ብቻ እንዳልሆኑ ቢረዱ እና ቢወያዩ ጥሩ ነዉ አ.አ አለ መሰለኝ ከዛ የመጣ ልጅ ነግሮኛል ግን እንደ እኛ ቲሸርት የላቸዉም

ኦቴዝ እንዲስፋፋ ማን ምን ማድረግ አለበት፣ መንግስት ኃለፊነት ወስዶ ማስፋፋት አለበት መንግስት በጀት ከለቀቀላቸዉ ሀኪሞች አናረግም አይሉም ምክኒያቱም እነሱም ልጅ ሰላለቸዉ የልጅ ሁኔታን ስለሚያዉቁት እና ከመንግስት ይህ ይጠበቃል

በዚህ ፕሮግራም እንደ እኛ ሁነዉ ተሰባስበዉ ቢያዉቁ፣ ቢረዱ ሌላ ሰዉ እንደላ ቢያውቁ ጥሩ ነዉ መድኃኒት እየተወሰደ ህይወት ይኖራል፣በአልጋ ለይ ሁኖም ህይወት ያልፋል

ለወደፊት ልጅ እንዲኖርሽ ትፈልጊያለሽ

ልጅ የእግዚአብሔር ፀጋ ነዉ ግን ልጄ እኔ እናቱ የሆንኩትን እንዲሆን አልፈለግም ቨይረሱ ከእኔ ወደ እሱ እንደይተላለፍ መከለከያ መድሃኒቶችን እጠቃምለታለሁ ልጄ የኔ ነገር እንዲገትማዉ/እነዲደግማዉ አልፈልግም ግን ሳይታሰብ እንኮ ከሆነ ለልጄ በህፃንነቱ ነዉ ምነግረዉ ከዘበሆላ ምንም እንደይሰማዉ እሱ ብቻ እንደልሆነ እኔና አባቱም እንደለበት እነግረዋለሁ/አስረደዋለሁ

ከኤች አይቪ ነፃ የሆነ ልጅ እንዲኖር ምታደርጊያቸዉ ጥንቃቄዎች ምን ምን ናቸዉ

ክትትል ማድረግ እና ሐኪሞችን መማከር እነዲሁም ስለታማ ነገሮችን ለየብቻ መጠቀም

የመድኃኒት አወሳሰድ ልምድ፤

መጀመሪያ ለይ ጤና ጣቢያ በሽሮፕ ነበር የጀመርኩት ከዛበኆለ ወደ ክኒን ተቀየረልኝ ፡፤ አክስቴ ፤የአክስቴ ባልናአባቴ እንድወስድ ያደርጉኛል ሰዓት እንደያልፍብኝ አለርም እቀጥራለሁ ስራ ቢኖረኝም እንኮ አቆርጬ እወስደለሁ ሁለት ሰዓት ለምወስደዉ አንድ ሰዓት ለይ አላርም እቀጥረለሁ ከእህቴ ቤት ከሆንኩ ታናነሽ ህፃናት ያስተዉሱኛል

አንድ ጊዜ አሳይመንት ለይ ሁኜ ሰዓት አሳልፌ ወንድሜ ተቆጥቶኝ ነበር እንደዚ አልጠብቅሽም ነበር ሁሉ ብሎኛል፤ ይህም ለኔ ብሎ ነዉ

ስለክኒን ብዛት በተመለከተ

የሆነ ሰዓት ለይ ራሴን ስቼ ታምሜ ነበር እና ብዙ መድሃኒት ተሰጦኝ /የጨጎራ ተጨምሮብኝ በዝቶብኝ ነበር እንጂ ከዛ በሆላ ምንም አያስቸግረኝም ፡፡ መድሃኒት ከሰዎች ፊት ለመዉሰድ ትፈሪያለሽ

አዎ ድንገት እንግዳ ቢመጣ ዉሃ ወደ ክፍሌ አስገብቼ እወስደላሁ አሊያ ወደ ዉጭ ወጣ ብዬ እወስደላሁ፡፡ ቀደም ብዬ ማድረግ ያለብኝን ነገር እወስደለሁ/አደርገለሁ፡፡

የጎንዮሽ ጉዳትን በተመለከተ

ማንኛዉም ነገር ሲሳራ ትቅምም የጎንዮሽ ጉዳትም አለዉ፡፡ በኔ ለይ እስካሁን የጎንዮሽ ጉዳት እስካሁን አልታየብኝም ፡፡ መድሃኒቱን በአግባቡ ከልወሰድኩት የሆነ ነገር እሆነለሁ ማለቴ አልጋ ለይ እወድቅ እችላለሁ/የአልጋ ቁረኛ እሆነለሁ/እታመማለሁ፡፡ አይገድልም ይበላል እንጂ ይህ በሽታ እኮ ይገድላል ይህ እንደይሆንም መድሃኒት በአግባቡ እወስደለሁ

ለወጣቶቹ ማን ምን ማድረግ አለበት ትያለሽ

እኔ ምለዉ ለኔ ብቻ ሳይሆን ድጋፍ ሚያስፈልገቸዉ አንድ አንድ ህፃናት አሉ

ለሀፃናት ድጋፍ ያስፈልጋል ፤ድጋፍ ሲባልም በገንዘብ ብቻ አይደለም በስነ ልቦና በፍቅርም መደገፍ /ፍቅር ማሳየት መንከባከብ አለባቸዉ ሁሉም ሰዉ ፍቅር ማሳየትመስጠት አለበት

ቤተሰብ ጋር ተገጭተህ መደሃኒት ልታቆርጥ ትችላለህ ስለዚህ ወለጆች በህፃናት ፊት በይጨቀጨቁ ይመረጣል /የቤተሰብ ጭቅጭቅ ህፃናት መድሃኒት እንደይወስዱ ያደርጋል፡፡ስለዚህ ወለጀች በህፃናት ፈት ፍቅር ማሳየት አለባቸዉ

መንግስትም ለባለሙያዎች ድጋፍ ቢያደርግ ምክኒያቱም እሁድ ቅዳሜ ለእኛ ብለዉ ነዉ በእረፍት ቀናቸዉ ሚገቡት /ግዴታም የለባቸዉም ፡፡ ከዚህ በተጨማሪ ምለዉ ሁሉም ሰዉ ቢመረመርና ራሱን ቢያወቅ ቢኖርበትም መድሃኒት እተወሰደ ይኖራል፤ ህይወት ይቀጥላል፡፡ አይደላም ይህ ኮቪድም ይገድላል ኤች አይቪም ይገድላል ሁሉም ሰዉ መማርመር አለበት ልክ እንደ ኮቪድ ኤች አይቪም በሰዓቱ ተመርምረን መድሃኒት ከልወሰድን ጊዜ አይሰጥም የጤንነት ሁኔታ ሲለወጥ ራሱ ሂጄ ቼክ ላድርግ ማለት አለበት

ፍቅር ከማሳየት አንፃር ከማህበረሰቡ ምን ይመስላል

ከማህበረሰቡ በኩል ሁለት ቦታ አለኝ

አንዱ፤ አንዳንዴ ጥሩ ማህበረሰብ ያጋጥማል ሌላዉ መጥፎ ማህበረሰብ ያጋጥማል/ መጥፎ ስል ጥሩ ፊት/ፍቅር መያሳይ ያጋጥማል፡፡ሌላዉ ፍቅር ማሳየት ስል ማህበረሰቡ ለእኛም ልክ እንዳ ልጆቻቸዉ ማየት አለባቸዉ፡፡

በመጀመሪያ ፈጣሪ ሲፈጥረን እያንዳንደችን ራቁታችን ነዉ የተወለድነዉ ፈጣሪ ለሁላችንም ፍቅር ሰቶናል ስለዚህ አሁን ለይ ገንዘብ ሚባል ነገር መጦ ሰዉ ሁሉ ገንዘብ ወደጅ ሆነና ከፍቅር ገንዘብ እያስበለጠዉ ይገኛል ብዙ ማህበረሰብ መድሃኒት እየወሰዱ ከማህበረሰቡ የተገለሉ አሉ እናም ማህበረሰቡ ፍቅር ቢያሳዩ ምክኒያቱም ቨይረሱ በሠላምታ አይተላለፍም በራሰችን እንዝላልነት ሚመጣ ነዉ ከዛም ራሳችንን መጠበቅ ነዉ ሌላዉ የ መ ህጎችን በመተግበር መካለከል ይችላል እና ሁሉም ሰው ራሱን አዉቆ ለሌሎች ፍቅር መስጠት አለበት

ከበላሙያ በኩል ፍቅር ከማሳየት አንፃር

ብዙሃኑ በለሙያዎች ፍቅር ይሰጣሉ/በፍቅር ያወያያሉ፤ ያስተናግደሉ፤ እንክብካቤም ያደርጋሉ ግን ከስንት አንዴ

አሁን ለይ አንድ አንድ ሚያመነጭቅ በለሙያ አለ/አልፎ አልፎ ምንጭቅ ምንጭቅ ሚያደርጉ በለሙያዎች አሉ ከስንት አንድ የበህርይ ለዉጥ አለ ይህም በስራ ጫና ሊሆን ይችላል ወይም በሆነ ነገር ተነዶ ሊሆን ይችላል ይህ ደግሞ ሁሌ ሳይሆን አልፎ አልፎ ነዉ ሚከሰት፡፡ በሰዉ ለይ ሚያገጥም ነገር ነዉ

በመጨረሻ ለይ ምትጨምሪዉ ነገር ከላ

በመጨረሻ ማለት ምፈልገዉ አባቴ ተስፋ ሰጦኝ ነበር ይህም መድሃኒት አልወስድም ስለዉ አይዞሽ ለአንድ ዓመት ዉሰጂ እና አንዴ ተወግተሸ ለአንድ ዓመት የሚሆን በመርፌ ሚሰጥ መድሃኒት እገዛልሻለሁ ጠብቂኝ ይል ነበር ፤ ይህ መድሃኒት ቢመጣ ለበለሙያም ለእኛም በጣም አሪፍ ነዉ ምክኒያቱም በየቀኑ መዉሰድ ሰዓት አለፈኝ፤ ረሳሁት ብሎ ማሰብ የለም፡፡ አሁን ለይ ሳስበዉ ግን አባቴ አዉቆ ተስፋ እየሰጠኝ እንደሆነ ነዉ የገባኝ፡፡ ይህ መድሃኒት ለአንድ ዓመት/ለስድስት ወር ሚሆን ቢመጣልን ጥሩ ነበር

**ተሳታፊ ሁለት**

**ከኤች አይቪ ጋር አብሮ መኖርን እንዴት ትገልፃዋለህ?**

አዎ ኤች አይቪ እኛ ፈልገነዉ የመጣነዉ አይደለም በቤተሰቦቻችን እንዝላልነት የመጣ ነዉ ግን የመጣዉን ነገር አምኖ መቀበል ያስፈልጋል ለምን በኔ ለይ መጣ ብሎ ያለግባብ አላስፈለጊ ነገሮችን ማድረግ አያስፈልግም ያዉ የመጣዉን ነግር አምነን ዶክተሮች ጋር አብሮ እየተግባበህ መከተል ነዉ ሚያስፈልግህ እና ኤችአይቪ ሲገኝብህ መድሃኒት በአግባቡ በመዉሰድ እንደማንኛዉም ሰዉ መኖር ይቻለል እና ጥሩ ደረጀ ለይ መድረስ ትችላለህ ሚከብድ ነገር የለዉም

ስለ ኤች አይቪ ስታስብ ምን አይነት ሰሜት ይሰማሃል?

አንዳንዴ ለጎደኞቼ አወጥጠህ መናገር አትችልም ያያ ተፅዕኖ ሚያደረስ ነገር ይኖራዋል አሁን ለምሳሌ ክፍል ወስጥ እንደዚህ ነኝ ብለህ ብትነገር በጣም ሚከብድ ነገር ሊደርስብኝ ይችለል በእርግጥ በቨይረሱ ምክኒያት የሆነ ነገር ልታስብ ትችለለህ መቼስ አታስብም ማለት አይደለም/ሌላዉ ይሰማ ይሆን/ይፋ /ሚስጢር ይወጣብኝ ይሆን ብሎ መጨነቅ አለ/ ግን ሚስጢሩ ከልወጣ እንደማነኛዉም ሰዉ መከተል ይቻለል ምጨነቀዉም ከማህበረሰቡ ሚመጠዉ መልስ ጥሩ ነገር ለይሆን ይችለል መለትም መገለልን በመፍረት ነዉ ምጨነቀዉ ይህ ከሆነ ደሞ ሊጎገኝ ይችላል

ያጋጠሙ ፈተናዎች አሉ? የለም

የደረሰብህ ጫና አለህ? የለም

እንዴት አወቅህ ማን ነገረህ?

መድሃኒት መዉሰድ የጀመርኩት ከህፃንነት ጀምሬ ነዉ በዚሁ ስለደግሁ ቤተሰብ እንደዚ ነህ ብሎ አልነገረኝም በራሴ ሂደት ነዉ ያወቅሁት እዚህ ማህበርሲመሰረት ከቡድን ዉስጥ 8/16 አበላት ነበርን ከነዚህ ዉስጥ አንዱ ስለነበርኩ ማህበሩ ግንዛቤ አስጨብቶናል ለጊዜዉ እንደዚህ ተብሎ አልተነገረኝም ዝምብዬ መድሃኒት እየወሰድኩ ለዉቅ የቻልኩት ይመስለኛል እዚህ ማህበሩ ለይ ስንቀላቀል መረጀ ስለሚሰጡን/ሲነግሩን መድሃኒት እየወሰድከ ስለመጣሁ ምንም አልመሰለኝም

ስትሰማ ሁኔታዎች ምን ነበር ላንተ?

ብዙ ጎደኞቼ አብሮ ስለነበሩ ቀድመዉም በተለያየ መንገድ አድርገዉ ይነግሩኝ ስለነበር ለምሳሌ መድሃኒቱ እድሜ ልክ እንደሚወሰድ በሽታዉ ይህ ነዉ ተብሎ በይነገርም እዚህ ማህበሩ ለይ በሚሰጡ ትምህርቶች/ለዉይይት ከሚነሱ ሳቦች/ ግልፅ እየሆነ መጣ በተጨማሪም 4 ክፍል ሳይንስ ለይም እንማር ነበር

ቨይረሱ እንደላብህ ከተረደህ በሆለ የተሰማ ስሜት ነበር?

ቤተሰብ ያዉቁ ስለነበር እኔን ይቀጠሉኝ ነበር ምንም አልተሰማኝም ግን ምፈራ የነበርኩ ትንሽ ከወጣ ብዬ ነበር ቤትም ይተወቅ ነበር ግን አልወጠም

ለሎች ራስህን አሰዉቀሃል ፤ የለም ከበተሰብ ዉጭ ሌላ አያዉቅም

ለመነገር አስበህ ታዉቀለህ/ለመነገርም ፍለጎት የለኝም ምክኒያቱም አሁን ህብረተሰቡ የተማረ ነዉ ቢባልም በተግባር ግን ይቀራዋል ከእነሱ ሚመጡ ቃለቶች ጥሩ አይደሉም መራረቅም ሊመጣ ይችላል

ቨይረሱ ያደረሰብህ ጫና አለ? ምንም የለም ጥሩ ነዉ

ጎደኛ /ፍቅረኛ አለህ የለም ለወደፊትስ እኔ እንጃ ሚኖረኝ አይመሰለኝም እንደ እኔ አሁን ህይወቴ ከተስተካከለና ከተሰካልኝ /ራሴን ችዬ መስተዳደርና ቤተሰብን ማገዝ ከቻልኩ/ቨይረሱ ስላለብኝ ብቻ ሳይሆን በይኖርብኝም ዝምብዬ መከተል ነዉ ምፈልግ ያን ያህል ፍለጎት የለኝም እና ከቤተሰብ ጋር መለትም ከእናቴ ጋር ና ከእህቶቼ ጋር አብሮ መኖርን ነዉ ምፈልግ

ስለ ሴት ጎደኛ ለወደፊት አያስፈልግም ትለለህ አሁን ለይ አያስፈልግም ግን ለወደፊቱ ሰዉ ነህ እና አይተወቅም

እጮኛ ምታረጋት ልጅ የኤችአይቪ ሁኔታዋስ ያንተ ምርጫ ያለባትን ነዉ የሌላበትን አሶ የሌላበት ሁና ምትቀበለኝ ከሆነ ችግር ያለዉ አይመስለኝም እሺ ብላ ከተቀበለችኝ በግንኙነት ጊዜ ኮንዶም መጠቀም ልጅ ከፈለግን ደሞ ቴክኖሎጂ እያደገ ስለመጣ ከኔም ከእሶም ዘር ተወስዶ ማደቀል ነፃ የሆነ ልጅ እንዲወለድ

**ስለ መድሃኒት አወሳሰድ**

ዛሬ በቀን አንዴ ነዉ ምወሰወደዉ ያዉም ማታ አንድ ጊዜ ድሮ ግን በቀን ሶስቴ እወስድ ነበር ይህ ያስቸገር ነበር አሁን ለይ ቀርቶል አሁን ለይ እየተሻሸለ የመጠበት ሁኔታ ነዉ ያለዉ ለወደፊትም በመርፌ ይሆናል ሚባል ወሬ አለ ዉጭ ለይ በመርፌ እየሆነ ነዉ ይባላል ዉጭ ለይ ያለች አክስቴም በዛ ዙሪያ ስለምትሰራ እንደዛ ተለኛለች ግን እዛ ለመድረስ መድሃኒት በስነስረዓት መዉሰድ አለብኝ

መድሃኒቱን ከሰዎች ፊት በነፃነት ትወስደለህ …የለም መድሃኒት ምወስደዉ ማታ ነዉ አንዳንዴ ማይመቹ ሁኔታዎች ሊያጋተሙ ይችላሉ በዚህ ሰዓት በማያስነቀ መልኩ መዉሰድ የሌላ በሽታ ነዉ ብሎ መነጋር ምክኒያት በመስጠት ማስተባበል

የጎንዮሽ ገዳት አለ ? የለም

መድሃኒት ሁሌ መውሰድ ይሰለቸሃል ወይ…? አስተውየዉ አለዉቅም

መገለልን በተመለከተ… እንደዚህ አለበት ተብሎ ሲወራ ሰምቼ አለዉቅም ግን አይተወቅም

ሌላዉ ብዙ አይነት ሰዎች እንደሉ አይቼለሁ ማለቴ መደሃኒት አቆርጠዉ የሞቱ ሁሉ እንዳሉ እና ከዓመትዓመት የተሸለ መድሃኒት እየመጣ ነዉ ለወደፊት ፈቱን የሆነ መድሃኒት እንደሚገኝ ተስፋ አደረገለሁ

ከአገልግሎት አሰጣት አንፃር ከበለሙያዎች በኩል ሚሰጠዉ እነክብካቤ መገለል እንዴት ታያዋለህ

ከበለሙያዎች በኩል ምንም ችግር የለም በጥሩ ሁኔታ ያስተናግዱናል፡፡ ሌላ ምለዉ ነገር የለም ያዉ ከጥናቱ ጥሩ ነገር እጠብቃለሁ፡፡

**ተሳታፊ** **ሦስት**

ከኤች አይቪ ጋር አብሮ መኖርን እንዴት ትገልፃዋለህ

ከኤች አይቪ ጋር መኖር ማለት ለኔ በሽታ ሚወድ የለም ነገር ግን በሽታዉ ቢመጣም ከሰዉ እኩል ሁኛለሁ ከሰዉ በታች አልሆንኩም እና ከኤች አይቪን ይጄ አድሎና መግለል ሰይደርስብኝ አለሁ

ቨይረሱ በመኖሩ ምክኒያት ሚሰማህ ስሜት አለህ …የለም

ምንስ ታስበለህ …እነደማንኛዉም ሰዉ መኖሬን አስበለሁ እናም በዛ ደስተኛ ነኝ ከዚህ የተለዬ ስሜት የለኝም

ያጋጠመህ ችግረ/ፈተና አለህ፡ አዎ በሽታዉ ትንሽ ክፍተት ከአገኘ የመበርታት በሕርይ አለዉ ነገር ግን በትርፍ አንጀት ምክኒያት ኦፕራሲ ተደርጌ ነበር በሰዓቱ ትንሽ ከበድ ነበር ለኔ ኦፕሬሽን በጣም ቆይቶ ነበር እስከ አራት ሰዓት ወስዶል መግል ነበረዉ በዛ በዛ ምክኒያት መድኃኒቶችም ከበድ ያሉ ናቸዉ እንደማንኛዉም ሰዉ ቀለል ያሉ አይደለም ብዙ መድሃኒትም እወስድ ነበር ይህንን ትልቁ ፈተና ብዬ እወስደዋለሁ፡፡ ሌላ ችግር የለም

ከኦፕራሲ ጋር በተያያዘ ምን ስሜት ተሰማህ፡ በሳዓቱ ምንም አልተሰማኝ ህመሜና ህመሜን ብቻ ነበር ማደምጥ እነደበረታብኝም ያወቁት ከወጣሁ በሆለ እንደኔ የተሰራለት ሰዉ ይህንን ያህል መድኃኒት ወሰድኩ ሲለኝ ያለብኝንና እሱ የሌለበትን ሳነፃፅረዉ እኔ የወሰድኩት መድሃኒት ብዙ ስለነበር ለካ እንደዚ ነኝ ብዬ ሳስብ ነበር

ምታስበዉ ምን ነበር፤ በወሰድኩት መድሃኒት ብዛት ነዉ ሳስብ የነበርኩት

ቨይረስ እንደለብህ ራስህን አሰዉቀሃል፡ ቤተሰብ ያዉቃል ለሌላዉ የለም

መቼ ያወቅሃዉ፡ በልጅነት ጀምሬ ነዉ መድሃኒት ምወስደዉ የተላለፈብኝም በጡት ነዉ በሰዓቱ ምንም አለዉቅም ነበር ቤተሰብም መድሃኒቱም የሌላ በሽታ እንደሆነ ነዉ ሲነግሩኝ የነበሩት…ነርሶን ስጠይቃት ነገረችኝ በደምብ ከአስረደችኝ በሆላ

እዚህ ማህበሩ ለይ አስመዘገበችኝ እስካሁን አለሁ

ያኔ ስትሰማ ምን ተሰማህ፡ በሰዓቱ እንደሌላ ሰዉ አደለሁም በተፈጥሮ ኮንፊደንስ አለኝ እነደዚህ አይነት ነገሮች ለይ አልደነቀፍም ስለዚህ ምንም አይነት የስነልቦነ ችግር አልደረሰብኝም፡፡

ለምንድነዉ ለጎደኞችህና ለማህበረሰቡ ቨይረስ እንደለብህ ያለሰወቅሃዉ/ያልነገርሃቸዉ፡ ከማህበረሰቡ አንፃር ማለትም ምታካፍለዉ ጎደኛ ችግሩ ችግሬ ነዉ አይልም እንደ አንተ/እንደ ራስ ስለማያየዉ በሽታዉን ለሌላ ሊዘረዝረዉ/ሊነግረዉ ይችላል በተለይ ስትጠላ ወይም በጨዋታ ጊዜ ፍታታ ሚባል ነገር አለ ማለትም በነገሮች መሳደብ ፤ማሽሞታት፤በዘይቤ አነጋገር ስለሚኖር በዚህ ምክኒያት አልነገርኮቸዉም፡፡ ራስህን ከወቅህ በሆለ የተሰማ ስሜት አለህ፡ አስቤዉ አለዉቅም

ኤችአይቪ በትምህርት ለይ በህርይ ለይ ያደረሰብህ ተፅዕኖ አለ? ፡

የለም

በአሁኑ ሰዓት እጮኛ አለችህ ? ፡ የለኝም

ለወደፊተስ የመያዝ እቅድ አለህ፡ አዎ ሰዉ ነኝና ፍለጎት ይኖራኛል፤ ከኔ ጋር ተመሳሰይ የሆነችዉን እመርጣለሁ፤ከቨይረሱ ቨይረስ ቢተላልፍም ይህንን ለመካላከል ሁለታችን ምክክር ማድረግ ለምሳሌ የግብረ ስጋ ግነኙነት ድግግሞሽን መቀነስ የሐኪሞችን ምክር መተግበር መድሃኒቱን ሰዓት ሳነሰልፍ መዉሰድ

ከኤች አይቪ ነፃ የሆነ ልጅ እነዲኖርህ ምታደርጋቸዉ ጥንቃቄዎች ምን ምን ናቸዉ

ልጁ ነፃ ሁኖ ሊወለድ ይችላል ይህን እዉን ለመድረግ ሴቶ ክትትል እንድታረግ ማድረግ እና ከሕክምና ሚሰጠዉን ምክር መተግበር

ስለ መድሃኒት አወሳሰድ ያለህ ልምድ ምን ይመስላል?

ሁሌም ከምሽቱ 3፡00 እወስደለሁ፤ ማን ነዉ ሚያስተዉስህ፡ አዎ አለ ሌላ ሰዉ ሰያስፈልግ ራሱ ህሊና ምግብ እንደ ምተስተዉሰዉ ሁሉ አምጣ ይልሃል፤ ማለትም ይርበሃል ሳይሆን ያስተዉሰሃል ወደ መድሃኒት ትሄደለህ ምትረሳህ ከሆነማ በሽታዉ እንዲፃና ተረገዋለህ ወይም ለራስህ ግድየለሽ ነህ ምንም ፍለጎት የለህም ማለት ነዉ ለማደን ስትፈልግ ግን ብዙ ነገር ታደርጋለህ ለምሳሌ፤ በየሰዓቱ መሄድ መዋጥ ማታ ኮስ በማይበት ሰዓት መድሃኒት በኪስ ና ዉሃ ይጄ እገባለሁ ሰዓቱ ሲደርስ ወጣ ብዬ ወስጄዉ እመለሳለሁ

ከመድሃኒት በተያያዛ የጎንዮሽ ጉዳት አለ፡ የለም ምናልባት ፊቴ ለይ ያለዉ ብጉር ኦፕራሲ ስሰራ ምጭ ነዉ ብዬ ነበር ለስድስት ወር አልታጠብኩም ነበር ያልታጠብኩትም ቁስሌ መረከዘ/ቁስል አመጣ መግል ያዛ/ ከዛ ዉሃ እንደይነቀ ተብዬ ነበር በዘዉ ዳነ ከዛበሆለ ነዉ ፊቴ እንደዚህ የሆነ …ቀስል ነገር/ጠበሳ ነዉ የሆነ ፡ ምችም፤ብጉርም ይድናል ፤ይህ ጠበሳ የተፈጠረዉ ከኦፕራሲ በሆለ ነዉ ስሰማ ደሞ ኤች አይቪ እንደዚህ ያመጣል አሉ ነገር ግን መድሃኒቴን አሳልፌ ስለማለዉቅ ችግሩ ከምን እንደመጣ እስካሁን አለዉቀዉም ለዚህ ብጉር ነዉ በሚል ህክምነም አለረግኩም ያ ነገር ከሆነም ሆነ ስላለወቁት ነዉ

የተቆም ርቀት እንዴት ነዉ መድሃኒት ለመዉሰድ ርቀቱ ያስቸግርሃል፡ አያስቸግርም ስሄድም ስመጣም ታክሲ አገኛለሁ

የታክሲን ወጪ በተመለከተ፡ ተማሪ ስለሆንኩ እኔ ምቀበለዉ ከእናቴ ነዉ ቤት ሳይኖረቸዉ ሲቀር ከጎደኞች መቀበል አለ ይህ ጉደይ ከበድ ሁኖ ሳይሆን አኔም ስራ ስለማልሰራ ፤አስቸጋሪ ነዉ ብዬ በልጠራዉም ትንሽ እንደ ቻሌንጅ እወስደዋለሁ

የመድሃኒት ብዛትን በተመለከተ

ድሮ ብዙ ስወስድ ነበር ከ3/4አመት ወዲ ግን አንድ ፍሬ ነዉ ምወስደዉ ይህ ደሞ ለአወሳሰድም ምቹ ነዉ ምክኒያቱም ማታ ማታ ለመዉሰድ ምቹ ነዉ..አንዴ ብቻ ስለሚሰጥ በጣም አሪፍ ነዉ

ከአገልግሎት አሳጣት አኮያ በተለይ በሆስፒታል በኩል ሚታይ ችግር/ክፍተት ከላ

ሙሉ በሙሉ መድሃኒት በናጣም ለሶስት ወር ሚሰጠዉን ለአንድ ወር መዉሰድ እንጂ የመድሃኒት እጥረት የለም

የበለሙያዎች አገልግሎት አሰጣት አኮያ ሚታይ ክፍተት አለ፡ የለም ድሮ ጀምረን ስለምነዉቀቸዉ ምንም ክፍተት የለባቸዉም ፡፡ አዉነት ለመነገር ከቤተሰቦቻችን ይልቅ በለሙያዎች አሰድገዉናል ብንል ማገነን አይሆንም በበለሙያዎች በኩል ሚሰጠዉ እንክብካቤ በጣም አሪፍ ነዉ፡፡

ከማህበረሰቡ ….

ከመንግስት አኮያ…ከሞላ ጎደል መንግስት ጥሩ እየሰራልን ነዉ ማሳያዉም ነገ ሚሰጠዉ መታወቂያ በጣም አሪፍ ነዉ ይህም በአጋጣሚ ወደ ሎላ አገር ብሄድ ይህንን መታወቂያ በማሳየት መድሃኒት መግኘት እችላለሁ፡፡

በቨይረሱ ምክኒያት የደረሰብህ ጭንቀት/የበህሪ ለዉጥ አለ?

ይህ እኔን አያስጨንቀኝም ግን ብዙ ምግብ እፈልገላሁ ምክኒያቱም በሽታዉን ለመቆቆም መድሃኒት ብቻ ሳይሆን ምግብም ያስፈልጋል፡፡ ይህ ነገር ነዉ እንጂ ሌላ ጭንቀት የለብኝም ጭንቀትን ሚያመጠዉ የግል ታሪክህ ነዉ ሌላዉ ደሞ ልጆች ከቤተሰብ ጋር ሲጣሉ ወይም ይህ ነገር ከልተደረገልኝ አልወስድም የሚሉ አሉ እንደዚህ አይነት በኔ ለይ የለም

**ራስህን ለሌሎች ያለሰወቅህበት ምክኒያት ምንድነዉ**

ማህበረሰቡ ያልሆነ ስም መስጠትና መገለል ስለላ በኔ ለይ በይደርስብኝም በቲቪ ያየሁትን ልንገርህ ሐዋሳ ለይ ልጅቱን አክስቶ ምትጠራት ቼች አይቪ እያለቸት ነዉ ከዛ ማህበረሰቡ ተቀበላና ኤችአይቪ ብሎ መትራት ጀመረ ..ትክክለኛዉ ስሞ የምስራች ነዉ..ግን ህብረተሰቡ ሚጠራት ኤችአይቪ እያለ ነዉ፤ በዛ ለይ ተደፈረች ልጅም ወለደች 18 ዓመቶ ነዉ በዚህ ተጎደች ግን መንግስት ቤት ሰጣት...የሞራል ካሳ ከፈላት ...የኛ ማህበረሰብ አስተሳሰብ ብዙም አልተቀየረም ነገ በኔ ሚባል ነገር የለም.. ይህንን ማሳወቁ ጉደይ/ግዴታ አደለም ነገር ግን ግዴታ ሚሆንበት ሁኔታ ለፈጠር ይችላል ለምሳሌ የመኪና አደጋ በሚደርስበት ሰዓት ለጎደኛ ልትነግረዉ ትችላለህ ጥንቃቄ እንዲያደርግ እሱ ለይኖረዉ ይችላል፤ምክኒያቱም ጎደኛህን እንደወንድሜ ማየት ስላለብኝ በዚህ ጉደይ ልትነግረዉ ትችላለህ እነጂ ወሬ ብለህ አትነግረዉም

ለወደፊት ራስህን የት ቦታ ለይ ደርሰህ ማየት ትፈልጋለህ ፡ የሰዉ ልጅ ነኝና አሪፍ ሀብት ኑሮኝ ከሁን በተሻለ ኑሮ ለይ ብገኝ ደስ ይለኛል

እዚህ ቦታ ለይ ለመድረስ ምን ማድረግ አለብህ/ማን/ምን ማድረግ አለበት

መማር በዛ ለይ ደሞ ሚመለከተዉ አካል መርዳት በገንዘብ ግድ ትምህርት ብቻ አይደለም በትምህርትም ጎበዝ ሁኖ ተመርቆም ስራ ለያገኝ ይችላል ፤ከተመረቁ በሆለ መንግስት ስራ ማስጀመር/መስገባት ማለትም ከቨይረሱ ጋር ሚኖሩትን ማስቀደም አለበት ብዙ እንደያስቡ ቢያመቻች ጥሩ ነዉ ሌላዉ መንግስት አዳዲስ ሀሳቦችን ቢያመጣ ልክ እንደ መታወቂ ካርድ እና ኦቲዜድ ፕሮግራም /ኦቲዜድ ፕሮግራም እኛ ለይ ብቻ ነዉ ያለዉ ለምሳሌ አ.አ የለም ሂጄ ስለየሁት ሳይሆን ከዛ የመጣ ልጅ ስለነገረን በኦቲዜድ ቲሸርት የላቸዉም

ከማህበረሰቡ ያለዉ አመለካከት እንዴት ነዉ

ለማህበረሰቡ ብትነግረዉ ነጋ ከነጋ ወዲያ ስትጠለ ሚስጥሩን ያወጣብሃል ባትነገረዉ ደገሞ አንተን አምኖ ተቀብሎህ ይኖራል ከነገርከዉ በሆለ ደገሞ ያኔ ሳትነግረዉ ያረገሀዉን ነገር ለምሳሌ መዳበስ ከሆነ ከነገርሀዉ በሆለ ለመዳበስ ስትሞክር እነዳትነካኝ ሊል ይችላል ገና የማህበረሰቡ አመለካከታችን አልተለወጠም በዚህ ጉደይ በማህበረሰቡ ለይ ችግር አለ ይህንን ቢሰሙና ቢቀየሩ

ሌላዉ በፆታዊ ጉደይ ትምህርት ቢሰጥ /ለምሳሌ በልጃገረዶች ዙሪያ/ ፡ በፆታዊ ግንኙነት በኩል ማህበረሰቡ ቨይረስ ያለበትን ይችን በኮንዶም ነዉ ማረጋት ፤በመላጣዉ ነዉ ማረጋት የሚል አመልካከት አለባቸዉ

በኮንዶም ነዉ ማደርጋት ሚሉት እነማን ናቸዉ

ቨይረስ ያለባቸዉ ሰዎች፡ ይህ ደሞ ስህተት ነዉ ኮንዶም ቢጠቀሙም በግንኙነት ወቅት ሃይለኛ ፍትጊያ ስለሚኖር የመተላለፍ እድሉ ሰፊ ነዉ እና በዚህ ዙሪያ ትምህርት ቢሰጥ ይህ ሀሳብ መቀረፍ ያለባት ጉደይ ነዉ

አንተስ ለወደፊት ጎደኛ በምትይዝበት ሰዓት በግንኙነት ወቅት ምታደርገዉ ጥንቃቄ እንዴት ነዉ

እኔ ስይዝ እንደ እኔ ፖዘቲቭ የሆነችዉን ስለምይዝ ኮንዶም እጠቀማለሁ የሚል ሀሳብ የለኘም

ከቤተሰብ አኮያ ፡ የመጀመሪያም የመጨረሻም ልጅ ስለሆንኩ ጥሩ እነክብካቤ ይደረግልኛል

**ተሳታፊ አራት**

**ከኤች አይቪ ጋር አብሮ መኖርን እንዴት ትገልፃዋለህ?፤**

ያዉ ኤች አይቪ ጋር መኖር ያዉ ማንኛዉም ሰዉ እንደሚኖር በሕይወት እንኖራለን የተለየ ነገር የለም የተለየ ነገር ምንለዉ ቨይረሱ በደሙ እንደለዉ ልክ እንደ አንድ ወጣት ከቨይረሱ ጋር አብሮ መኖር ነዉ በዚህ ኑሮ ሁኔታ ዉስጥ ስኖር ቨይረሱ ይበልጥ እኔን እንደይጎደኝ ያሉትን ጥንቃቄዎችን ማድረግ ያለብኝ ነገሮች እያደረግሁ እኔንም ሳይጎደኝ ካሰብኩበት እንድደርስ የተሸላ ጤንነት እንዲኖረኝ የዕለት ተዕለት ተግባርን ጥንቃቄ በተሞለበት ሁኔታ በማድረግ ፤ ከፀረ-ኤች አይቪ መድሃኒትን በአግባቡ በመዉሰድ ፤የጤና ክትትል በማድረግ ፤የቨይረሱን መጠን በመከታተል ያለኝን የጤና ሁኔታ በመወቅና ከሐኪሞች ጋር ምክክር በማድረግ የተሸለ ጤና እንዲኖረኝ አደርጋለሁ፡፡

**ስለኤችአይቪ ስታስብ ምን አይነት ስሜት ይሰማሃል**

አመለካከቴ በፊት ልጅም ስለነበርኩ ሲነገረኝ በወቅቱ ከማህበረሰቡም አመለካከት ጋር ስታያይዘዉ ባጣም አስቸዳሪ ነዉ እንደገና ያለህን ኮንፊደንስ ሊነካህ ይችላል ልጅም ስለሆን እኩል ከጎደኞቻችን ጋር መጨዋት ማንችል ይመስለናል በሆነ አጋጣሚ ቢያዉቁብኝ የመግለልና እንደዚህ ከማህበራዊ ግንኙነት የማዉጣት ሂደት እንደ እነሱ/ፍሪ/ ነፃ ሁኜ ብዙ ነገሮችን አብሬ ማድረግ እንደማልችል አይነት ስሜት ይሰማኝ ነበር

አሁን ለይስ ይህ ፤ አሁን ለይ እዚህ ጤና ክትትል በማደርግበት ሰዓት በየጊዜዉ በሚሰጡ ምክሮች አልፎም እንደዚህ በሉት ማህበረት ፀረ-ኤችአይቪ መድሃኒት ተጠቃሚ ወይም ደሞ ቨይረስ በደማቸዉ ያለበቸዉ አፍላ እድሜ በሚገኙ ወጣቶችና ወጣቶች ማህበር በመኖሩ በዚህ ማህበር ስንታቀፍ እዉቅና እየተፈጠረልን ከነ ሲስተር ከዶክተሮቻችን ጋር የርዕስበርዕስ ምክክር እያደረግን አሁን እኔ በለሁበት ደረጀ በዚህ ማህበር ዉስጥ ከልጅነት ጀምሬ ነዉ ያለሁት እና በህፃናት ህክምናና ክትትል ክፍል ጀምሮ እስካሁን አባል ሁኜ ቀጥያለሁ ከአባልነት በላፈ ደሞ ኦቲዘድ የተሰኘ ፕሮግራም አለ ይህ ፕሮግራም ምንድነዉ ተማሪዎች ከሰኞ እሰከ ዓርብ በሉ የስራ ጊዜያት ዉስጥ ለመገልገል አመቺ ለይሆን ይችላል ተመሪዎች በትምህርት ምክኒያት ኦቨርላፕ ሊየርግባቸዉ ና ለይመጡ ይችላሉ ምክኒያቱም ከሩቅ ቦታ ከወረደም የሚመጡ ሰዎች አሉ ይህንን በአመከለ መልኩ ቅዳሜ ለሰረቪስ ክፍት እነዲሆን እና አገልግሎትን በኦቲዜድ ፕሮግራም ለተማሪዎች ብቻ ከ19 በታች እንዲሆን ተደርጎ በዘማህበር ዉስጥ የረሴ ቡድን አለኝ የቡድኔም ተወካይ ነኝ እና በበቂ የሆነ ግንዛቤም አለኝ የመነቃቂያ ትምህርት እሰጣለሁ አወያያለሁ በዚህ በኩል እና ሲስተር ስልጣነዉን ያመቻቹልናል እንደ በድን መሪም ከኔበታች የሆኑትን ህፃናትን ኬር/እንከበከበለሁ/ አደርጋለሁ በዚህ ምክኒያት የሻምፒዎን ተወካይ ነኝ እነደዚህ አይነት ዉይይት ስለምነረግ እየተለመመድን መጠናል ከዚህ በፊት ለክትትል ስመጣ ከእናቴ ጋር ነበር አሁን ግን ብቻዬን መጥቼ መድሃኒት እወስደለሁ ቨይራል ሎድ አሰራለሁ ለራሴ ኃለፊነት በመውሰድ ህክምና አደርገለሁ በዚህ ማህበር ስታቀፍ የነቃ ተሳጥፎ ያለዉ ተብዬ በመመረጥ ለሻምፒዎንነት እቹ ተወዳዳሪ ነኝ ከጎደኞቻችን ጋር የአቻ ለአቻ ዉይይት እናደርጋለን የመወያያ ደብተሮች ከጤና ቢሮ ይሳጠናል እሱን እያነበብን ለልጆች እዉቅና እንፈጥራለን

**ያጋጠሙ ፈተናዎች አሉ**

የስነ-ልቦነዊ ጫና ይኖራል ለምሳሌ ቤት እንግዳ ሊመጣ ይችላል ስለማልነግረቸዉም ጭምር መድሃኒት ከእነሱ ተደብቄ ለመዉሰድ በማደረገዉ ነገር የስነልቦነ ጫና ይኖራል እንግዳ በለበት ሰዓት ዉሃ በመያዝ ወደ ምኝታ ቤት ገብቼ መድሃኒት ጨርሼ ነዉ ምወጠዉ የራሴ ልምድ.. ከሌሎች ልጆች የሰማሁት ተሞክሮ ደሞ አንደንዶች እነደዚህ በመይመች ሁኔታዎች ወሰጥ ሲሆኑ መድሃኒትን በምራቅ ሊወስዱ ይችላሉ ያ ደሞ በራሱ ለመዋጥ ምቹ በለመሆኑ በሆደቸዉ ለይ ለያርፍ ይችላል ፤ጉሮሮ ለይ ሊቀር ይችላል እና መድሃኒቱ በአግባቡ ትቅም ለይ ለይዉል ይችላል እና ዉሃ በሚያስፈልገቸዉ ጊዜ ዉሃ ሳያገኙ በምረቅ ብቻ መውሰድ ፤ መድሃኒት መቆረት በጎደኞቻቸዉ ምክኒያት ወደ ሱስ የመግባት ፈተናዎች ያጋጥማሉ

አሁን ለይ ይህ የስነልቦነ ጫና የለብኝም

ከቨይረሱ ጋር እንደምትኖር ማን ነገረህ/እንዴትስ አወቅህ/ማን ነገረህ/የት/ሁኔታዎችስ እንዴት ነበር?

እዚህ ክትትል አደርግ ነበር የጀመርኩትም በህፃንናት አይደለም ለስምንት አመት ያህል መድሃኒት አልወስድም ነበር የምርመራ ዉጤቶች ይላያያሉ እናም መድሃኒት አልወስድም ነበር ፤ ከዛ በሆላ 2ኛ ክፍል እያለሁ ወደ 9/10 አመቴ ነበርኩ እና ከ15 አመት በታች የሆኑት ህፃናት መድሃኒት መዉሰድ አለበቸዉ ስለተበላ መዉሰድ አለብህ ተባልኩ በዚህ ሰዓት ደሞ እናቴ መውሰድ የለባትም በምግብ ብቻ መቆቆም ይችል ነበር ብለ ነበር ፤አይ አስገዳጅ ሁኔታ ነዉ ተበላችና መድሃኒት ጀመርኩኝ መድሃኒቱን ብዙም ስለልለመድኩት አስቸግሮኝ ነበር በተለይ እንክልፍ እምቢ ብሎኝ ነበር

እንደተነገረህ ስትሰማ ምን ስሜት ተሰማህ ፤ መድሃኒት እንድወስድ የተነገረኝ ቀን ያዘን ቀን ለምንድነዉ ምወስደዉ የሚል ጥያቄ ወዲያዉኑ መጣልኝ ለምን የሚል ጥያቄ ስለመጠብኝ እናቴ ወደ ምኝታ ክፍል አስገብታ በቨይረሱ ስለተያዝክ መድሃኒት መዉሰድ አለብህ መደሃኒት በመዉሰድ እነደማንኛዉም ሰዉ ጤናኛ ሁነህ መኖሮ ትችላለህ ብለ ነገረችኝ የመድሃኒት ሰዓት እንደያልፍብህ አላርም እቀጥርልሃለሁ በማለት የስነልቦነ ጫና እንደይደርስብኝ ብዙ አርጋልኛለች እነዲሁም ከጨዋታ እየተጠረሁ ስወስደ ነበር ጠዋትና ማታ ነበር ምወስደዉ መድሃኒቱ እየተሻሸለ መጠና በቀን አንዴ ሀኖል

በመጀመሪያ ለይ ጫና አለ ማላትም መድሃኒት ለምን እወስዳለሁ ሌላዉስ መቼ ይወስዳል እንደ ሰዎች ምግብ ብቻ በመውሰድ እኖራለሁ በምልበት ሰኣት እናቴ በደንብ አስረደችኝ ከዛበሆለ አኔም አለስቸገርኩም

ራስህን ለሌሎች አሳውቀሃል፤ የለም አለሰወቁም፤ ግን የጋራ ምነደርገቸዉ ሊኖሩ ይችላሉ ለምሳሌ የደም ልገሳ በሚኖርበት ሰዓት ለምን አትለግስም የሚል ጥያቄ ያስነሳል በዚህ ሰዓት ለበስ በማድረግ መፍትሄ ማፈላለግ ኪሎ ስለማይሞለኝ ነዉ በማለት መሰለፍ ወይም ልምዱ የለኝም በማለት የሀሳብ ለዉጥ ማድረግ

ለሌሎች ማሳወቅ ያልፈለግክበት ምክኒየት ምነድነዉ?

አንደኛ የአመለካከት ችግር በማህበረሰቡ ለይ ስለ አለ ማህበረሰቡ የነቀ ነዉ ብዬ አለስብም ህብረተሰቡ ከሰማ ከማሀረበራዊ ህይወት ሊያገልል ይችላል ፤ እንዲሁም መጠቆቆሚያ ሊያደርጉኝ ይችላሉ ከሚል አንፃር እነዲሁም ኮስ በምንጨወትበት ሰዓት ለረሳቸዉም ሆነ ለኔ ብለዉ የሆነ ነገር ሊነገሩ ይችላሉ በጋራ ሚያገነኙ ነገሮች ለምሳሌ በአንድ ሐይለንድ ዉሃ መጠጣት አብሮ መብላት አለ በዚህ ቨይረሱ እንደለበኝ ከወቁ ከእሱ ጋር አልበለም አልጠጣም ሊሉ ይችላሉ ከአመለካከት እጥረት እና እኔ በዛ ሰዓት የእነሱን አመለካከት ክፍተት ሰይሆን እኔ ከእነሱ ጋር አብሮ መከተል እንደማልችል እረደለሁ በዚህም የስነልቦነ ጫና ይደርሳል

**ኤችአይቪ በትምህርት ለይ ያደረሰብህ/ሽ ተፅዕኖ ፡** እግዚአብሔር ይመስገን ብዙም የለም

በፊት ለይ ፆም በሚሆንበት ሰዓት መመገብ ሊኖርብኝ ይችላል ሌላዉ ሲፆም ለምንድነዉ ማይፆመዉ የሚል ሀሳብ ይነሳል በዚህ ጉደይ ሳስብ ነበር አሁን ለይ ግን መድሃኒት ማታ አንዴ ስለምወስድ ምንም አለስብም እድሜም ከፍ እያለ ሲሄድ ግንዛቤዉ እየጨመረ ይሄዳል

በአሁነ ሰዓት የሴት ጎደኛ አለህ ፡ የለኝም እንደዚህ አይነት ለይ በርግት እዚም ትምህርት ስለሚሰጥ ብዙ አይነት ማህበራዊ ግንኙነት ኖራል ፆታዊ ፍለጎትም በዚህ እድሜ ሊከሰቱ ይችላሉ ግን ይህንን ነገር እንዴት ነዉ ማሰተነግደ ምችልዉ ሚለዉን ከዚህ በፊት ብዙ ስልጠና ወስጄለሁ አሁን በለዉ ነገር ምንም አይነት ፆታዊ ግንኙነት የለኝም ከክፍል/ከሰፈር ጎደኝነትበለፈ የለም

ለወደፊቱስ የመያዝ ፍለጎትስ ፡ ህይወት በአንድ መስመር ለይቀጥል ይችላል የሆነ እድሜ ለይ በጋራ ምትኖርበት ጊዜ /ብቻዬን ማልኖርበት ጊዜ ይመጣልና ሁሌም አስባለሁ ሳድግ ትምህርት ጨርሼ ዩኒቨርሲቲ ተመርቄ የራሴን ስራ ይጄ ቤተሰብ መስርቼ መኖር ሚለዉ ነገር ያሳስባል ያን ሳስብ ደሞ እነዴት ነዉ አካሄዱ ሚለወን ራሴን አጠይቃለሁ ፡ለዚህም ልክ እንደ እኔ ኑሮ/ጤና ሁኔታ ውስጥ ያለችዋን አንዲት ሴት የግል የሕይወቴ አካል በማድረግ መከለከያዉን በመጠቀም የተለያዩ ነገሮችን በመጠቀም ህክምነዉ የደረሰበትን አክም በመጠቀም ነፃ የሆነ ልጅ የመዉለድ ሀሳብ አለኝ እሰከሁን በለኝ ደረጀ የተሸላ ኑሮ በመኖር ጤነኛ ልጅ አንዲኖረኝ ነዉ ፍለጎቴ

**ስለ መድሃኒት አወሳሰድን በተመለከተ**

ድሮ ስወስደዉ የነበርኩት በብዘት አንደነገርኩህ ድሮ ጠዋት እና ማታ ነበር አሁን ለይ በቀን አንዴ ማታ ብቻ እወስዳለሁ ይህም በቨይረሱ መጠንና በበሽታዉ ሁኔታ ሚወሰን ነዉ፤ የኔ የቨይረስ መጠን አሪፍ/አንደኛ/ በሚባል ደረጀ ነዉ ያለዉ እና በዛ ሁኔታ ስለለሁ በሽታን መቆቆም በምችልበት ደረጀ ለይ ስለሆንኩ አንድ ፍሬ ብቻ ነዉ ምወስደዉ

**መድሃኒቱን ከሰዎች ፊት የመዉስድ ልምድ ከፍራጫ አንፃር**

**በ**ርግት ሚያወቁ ሰዎች አሉ ቢኖሩም ከህይወት ስለመይበልጥ እወስዳለሁ

የጎንዮሽ ጉዳትሰ አለ፡ የለም

የተቆሙ ርቀትስ በተለይ ከትራነስፖርት ወጪ ጋር በተያያዘ፡ በርግት በፊት ነበር አስብ ነበር ሁልጊዜ በየወሩ መመላለሱ እንዴት ነዉ ይህ ሁኔታ በሚል አስብ ነበር አሁን ግን ወደዚህ ሰፈር ስለመጣን አያስቸግረኝም

ግን ከጎደኞቼ ጋር ስንወያይ ቅርብም ከሩቅም እሰከ ወረደ ድረስሚመጡ አነስተኛ ገቢ ያላቸዉ ልጆች ስላሉ አንድአንድ ገዜ በዚህ ፕሮግራም ትራንሰፖርት ይሻፈናል ተብሎ ስለሚመጡ በጀት የለም በሚባልበት ጊዜ ሲቸገሩ እናያለን እኔንም ይጠይቁኛል/ተባበሩን ይላሉ፡ ከገቢ አንፃር ችግር አለ

ለወደፊቱ ራስህን የት ቦታ ለይ ትጠብቀዋለህ

ትምህርቴን በዩኒቨርሲቲ ተምሬ ጨርሼ በሶሻል ዘርፍ የመንግስት አማካሪ አካል መሆን ነዉ ምፈልግ ይህን ያልኩበት የራሴ ምክኒያት አለኝ አንደኛ ከለኝ ልምድና ከተሰዕቶ/ጊፈት/ ሁኔታ

ሁለተኛ ትምህርት ቤት ለይ ምሳተፍበቸዉ ክባባት ያሉትን ሰዎች ሳማክር በዚህ ዙሪያ ብትሰራ ጥሩ ነዉ ስለሚሉኝ

ከተቆማት ምን ይጠበቃል

የተለያዩ ተቆማት በተለያዩ መስክ ድጋፍ ያደርጋሉ

ተቆማትም የመንግስት አካልም ግብረ ሰናይ ድርጅቶችም ትኩረት ሰጠዉ መስራት ይኖረበቸዋል ለምሳሌ እኔ ያገኘሁትን እድል ያለገኙት ቤት ያሉት ብዙዎች ናቸዉ፤ ብዙ ተሰአቶ ያለቸዉ ራሳቸዉን በሱስ የደበቁ ሊኖሩ ይችላሉ መንግስትም ትኩረት ሠጦ አምራች ሀይል እንዲኖር ማድረግ

በሆስፒታል እየተሰጠ ያለዉን አገልግሎት በተለይ በበለሙያዎች በኩል ሚሰጠዉ አገልግሎት እንዴት ነዉ

በበለሙያዎች በኩል ምንም ችግር የለም በዚህ አጋጣሚ ሆስፒታሉን ማመስገን ምፈልገዉ እንደዚህ አይነት ዉስጥ ያሉ ሰዎችን የስነልቦነ ምክር ሲሰጡ እንክብካቤ ሲያረጉ ብዙ ነገር እያረጉልን እንደ ተጨማሪ ወላጅ ሁነዉ እንደ ልጅ በማንካበከብ የስነ ልቦነ ምክር በመስጠት ደረጀ አመርቂ ስራ ሰርተዋል እየሰሩም ይገኛሉ እኔ በ7/8 አመት በየሁት ሁኔታ፡፡

ዋናዉ ነገር በዚህ ጤና ሁኔታ ውስጥ ስትኖር/ቨይረሱ ጋር/ የስነ ልቦነ ጫና ነዉና ለዚህም በዚህ ፕሮግራም የምክር አገልግሎት አግኝቼለሁ ነገር ግነ ይህንን እድል ያለገኙ ልጆች ሊኖሩ ይችላሉ ስለዚህ ለእነዛ ልጆች ምን አይነት እድል እንፍጠር በሚለዉ ዙሪያ ቢሰሩ መልካም ነዉ ሌላዉ በገቢ ራሳቸዉን እንዲችሉ ሁሉም የበኩሉን ድርሻ ቢያደርግ በኮሚቴ ተዋቅረዉ የስራ ዕድል ቢፈጠር ፤ ማህበረሰባችን እዉቅና አንዲኖረዉ ቢደረግ ኤች አይቪም ልክ እንደ ስኮር ደም ግፊት እንክብካቤ ያስፈልጋል

**ተሳታፊ አምስት**

**ኤችአይቪ ጋር መኖርማለት ላንቺ ምን ማለት ነዉ እንዴትስ ትገልፂዋለሽ**

ኖርማል ሰዉ ነኝ ማንኛዉም ሰዉ እንደሚኖረዉ ነዉ እየኖርኩኝ ያለሁት ምንም ነገር አልጎደለብኝም በሕይወት አለሁ ከሁሉም የተሸልኩ ነኝ ኤች አይቪ ኤድስ በሽታኛ መሆኔን እንዲሁ ሰዉ አይቶ ሊያዉቀኝ አይችልም ውስጤን እስከለወቀ ደረስ ማለት ነዉ እዚ አንድ አንድ ሰዎች ኤችአይቪን እንደ ሌላ ነገር ያዩታል ይቺ እኮ ኤች አይቪ አለበት ትገላለች አይባልም እንደዛ ምክኒያቱም እሱ ከሚኖረዉ በለይ ነዉ እኛ እየኖርን ያለነዉ ቼክ ከሚደረግልን እኛ ምርመራ በምነደርግበት እሱ በዓመት አንዴ ሂዶ ለማያደርገዉ እኛ ግን በየወሩ ምርመራ ማድረግ ቀላል ነገር አይደለም ለእኛ፡፡ እና ምንም ማለት አይደለም እሱ

**ከኤች አይቪ ቨይረስ ጋር መኖርሽን ሰታስቢ ምን አይነት ስሜት ይሰማሻል**

ምንም አይነት ስሜት አይሰማኝም ምክኒያቱም ያወቅሁት በቅርብ ጊዜ ውስጥ ነዉ በህፃንነቴ በዉቅ ኑሮ ትልቅ ተፅዕኖ ይደርስብኝ ነበር ምክኒያቱም አሁን ግን እኔና እኔ ብቻ ስለምነዉቅ ምንም አይመስልም

**ሀሳብ/ትካዜ ፤ ድብርት ውስጥ መግባት እንደዚህ አይነት ስሜት አይሰመሽም** ፡ አዎ

**በቨይረሱ ምክኒያት የደረሰብሽ ጫና አለሽ**

ድሮ ለይ እንደላብኝ አለውቅም ነበር ግን የስነ-ልቦና ችግር ነበረብኝና ማንም ስለማያዉቅ እናቴና እኔ ብቻ ነበር ምናዉቀዉ እና ዶክተሩ ራሱ ሊያውቅ አልቻለም እንዳለብኝ አያውቅም እሱ እና መሃል ለይ እኔ ረሴ ነኝ ያቆምኩት እዚህ ድርጅት ከገባሁ በሆለ ነዉ የተውኩት እንጂ **ሀይለኛ የሆነ የሰነ-ልቦና ችግር ነበረብኝ ሀይለኛ የሆነ የስነ-ልቦና ችግር ስትይ፤ዘርዘር ማድረግ ትቺያለሽ**

አዎ፤ለምሳሌ ከማንም ጋር አልነጋገርም ከትምህርት ቤት እመጣለሁ እመራለሁ እዘምራለሁ ስገባ ቤቴ ነኝ በሬን እዘጋለሁ አንድአንድ ድርሰቶችን እደርሳለሁ

**ምን አይነት ድርሰት**

ለምሳሌ፡ ልጆች ከምናያቸዉ አንፃር ትምህርት ቤት ነዉ ምዉለዉ ከማያዉ አንፃር አንድ ስለ ሀገር ፍቅር አንድ አንድ እኛ ማህበረሰብ ለይ የውጩን ዓለም እየተከተልን ነው ያለነው እና እነሱን ነው እኔ ስፅፍ ለየት ያለ ስሜት ሚፈጠር ነገር አለ እና ሰዎች አብደ ነው ወይ ብለው ራሱ ያምናሉ ወይም ፍቅር ይዞት ነው ብሎ ራሱ አንድ አንድ ሀሳቦች ይመጡልኛል፡፡ የነገሩኝ ማለት ነዉ እና መሃል ለይ ግን ተረድተዉኛል መፃሐፌን ሰሳያቸው ማለት ነዉ እና ብዙ ነገር አለ ግን በእኔ ለይ በኤች አይቪ ኤድስ ተፅዕኖ አልደረሰብኝም

**ይህ ተፅዕኖ የደረሰብሽ ኦቲዜድ ፕሮግራም ለይ አባል ከመሆንሽ በፊት ነው ወይስ በሆለ፤**በፊት

ሌላስ ተጨማሪ የደረሰብሽ ነገር አለ

ሌላው ማንንም አለዳምጥም የራሴን ሀሳብ ብቻ ነው የማራምደው አንድ እናቴን ብቻ ነው ወንድምቼንም አለያቸውም ከትምህርት ቤት ይምጡ አይምጡ አይመለከተኝም

**ለምን** ፡ እሱን አለውቅም

**አሁንሰ፡** አሁን ለይ እነደዚህ አይነት ነግር የለም

**እንደዚህ ስትሆኚ ምን ይሉሻል፤** ብዙ ነገር ይሉኛል

ዝም በምልበት ጊዜ፤ ፍቅር ይዞት ነው ብለው ሚያስቡ ሰዎች አሉ ግን እናቴ ብቻ ስለምትረደኝ ዝም ነው ያለች፤ እኔ ወላጅ አልባ ነኝ ግን አንድ ድርጅት ውስጥ ነው ምነድገው እንደዛ ብለህ ስለምታስብ ምንም አይመስልም ፤ እያደግሁት ያለሁት የስ ኦ የስ በሚባል ድርጅት ውስጥ ነው

**ኤችአይቪ ቨይረስ እነደለብሽ ማን ነገረሽ/እንዴት አወቅሽ**

እኔ ያወቁሁት የነገረኝ አሁን የለም ባሕር-ዳር ውስጥ አይደለምያለው ግን ሽምብጥ ጤና ጣቢያ በምከታተልበት ሰዓት ልጠይቀቸው ስል ነው እነሱ ራሳቸው የነገሩኝ በ14 ዓመቴ ነው ያወቁት ፈለገ ህይወት ከመምታቴ በፊት እና እዛ ሂጄ ልጠይቀው ልክ በሩ ለይ ስደርስ ነይ እፈልግሻለሁ አለኝ ከዛ እኔ ብቻ አልነበርኩም እዛ አብሮኝ ሚያድጉ ልጆችም ስለነበሩ አብረን በጋራ ነው ያወቅን፤ ሲነግሩኝ ምንም አልተሰማኝም ፤እኔን የገረመኝም እሱ ነው ፡ይህ የተነገረኝም እዘው ሽምብጥ ጤና ጣቢያ ነው፡፡

**ኤችአይቪ አለብሽ ብለው ሲነግሩሽ /ስትሰሚ ምን ተሰማሽ**

የሆነ ሰዓት ለይ መደሃኒት አልወስድም እንደልኩ ትዝ ይለኛል ከዛበሆለ ነው ወደዚህ እንድመታ የተደረገው መድሃኒት አልወስድም ስላቸው ወንድሞቼ ጎዳኞቼ እዚህ እነደሆኑ አለውቅም ነበር እኔ ከዛ በሆላ ወንድሞችሽ አሉሽ ሲሉኝ ነው ወደዚህ የመጣሁት እነሱም መክረውኛል፡፡

**ለምንድነው አልወስድም ያልሽው ምክኒያት**

መድሃኒት አልወስድም ያልኩበት ምክኒያት ስለሰለቸኝ ነው

ሁልጊዜ መውሰድ ለምንድነው ማትነግሩኝ ነው እኔ

**ከቨይረሱ ጋር እንደምትኖሪ ራስሽን ለሌላ አሳውቀሻል/ሌሎች ያውቃሉ**

አያውቁም

እናቴ ናት ምታውቀው ፤አሳዳጊዬና የድርጅቱ ኃለፊ ናቸው ሚያውቁ ሌላ ማንም አያውቁም

**ለሌላ ሰው አልነገርሽም**

ምን ይሳራል፡ ነገርኮቸው አልነገርኮቸው ለእነሱ ከተጠነቀቁላቸው ምን ይሳራለቸዋል

**እንደይተላለፍ ጥንቃቄ ታደርጊያለሽ፡**

አዎ

**ራስሽን ከአሳወቅሽ/ካወቁ በሆለ ያገላሉሽ ሰዎች አሉ/መገለል ደርሶብሻል**

የለም፡ በምጠይቀቸው ጥያቄዎች ሁሉንም ነገር ያደርጉልኛል ምክኒያቱም እነሱ ሚያስቡት ይሰማታል ብለው ስለሚያስቡ ማንኛቸውም ወንድሞቼ በደማቸው ያለው ቨይረሱ የጠየቅሆቸውን ነገር ሁሉ ያደርጉልኛል፤ ቼክ አፕ በየአመቱ አለን የሆነ ስሜት ከተሰማን ለወጥ ከልንባቸው ፊታችን ከተለወጠ ወዲያውኑ ነዉ ህክምና ሚወስዱን እዚኛው ሰይሆን የራሳችን አለ ጋምቢ አለን እንሄድና እንታያለን

**ቨይረሱ ትምህርትሽ ለይ /በሕርይሽ ለይ ያደረሰብሽ ተፅዕኖ አለ**

ምንም ነገር የለም፡ ስኮለርሽፕ ደረሰሽ ስባል እኔ እኮ በደሜ ኤች አይቪ አለብኝ እንዴት ነው ልሄድ ምችለው ስል ምንም ችግር የለም ሲሉኝ ፤እሺ ብዬ ነው የተቀበልኩት ግን ሌሎች ልጆች ሚያወቁኝ ማለት ነው እነዚህ ወንድሞቼ እንዴት ግን እነደዚህ አልጠየቅሽያቸውም እንዴት ዝምብለሽ ትቀቢያለሽ አመነሽ ስኮለሩን ነው ያሉ ምንም አለልኮቸውም እነሱ ምንም ነገር አለጠየቁኝም

**ስኮለር ደርሶሽ ነው?**

አዎ ሰሞኑን አዲስ አበባ እሄደለሁ

ቼክ ተደርጌም ችግር የለውም ብለውኛል

አዲስ አበባ ቃለመጠይቅ ተፈትኜ ወደ ጋና እሄዳለሁ

ጎደኞቼ እንዴት አልጠየቅሺየቸውም ዝምብለሽ እነዴት ትለፊያለሽ ነው ያሉሽ ስድስት አምት ሙሉ ነው የለፋሁበት ይህንን ከስድስተኛ ክፍል ጀምሬ ግነ መጀመሪያ ሲደርሰኝ ቨይረስ አለብኝ መሄድ እችላለሁ ወይ ብዬ አልጠየቁም ዝምብዬ ነው የተመዘገብሁት ግን አሁንም ችግር የለም ነው የተበልኩት እና ምንም አይነት ተፅዕኖ ትምህርት ለይ አለደረሰብኝም

**በበህርይ ለይስ፡** የለም

**በአሁኑ ሰዓት የወንድ ጎደኛ/ፍቅረኛ አለሽ፡** የለም

**ለወደፊትስ ባል ማግባት/ጎደኛ የመያዝ ሀሳብ አለሽ፡** ለሚከተለው እግዚአብሔር ያውቃል ነዉ የነገውን ውሎችንን እንኮ አናውቅም ስለዚህ ይህንን ልነግርህ አልችልም

**አስበሽበት አታውቂም፡** አለውቅም

ል**ጅ እንዲኖርሽስ፡** አለውቅም

**ስለመድሃኒት አወሳሰድ በተመለከተ፡** በፆምም ለኔ ይመቸል ምክኒያቱም በቀን አንድ ጊዜ ስለሆነ ማታ አንድ ሰዓት ነው ምወስደው፡ እህቴ ወይም እናቴ ናቸው ሚያስተውሱኝ ፡ ሰዓት ከያዝኩኝ እኔ ራሴ አስተውሳለሁ፡ ጥናት ለይ ስለምሆን አብዘኛውን ጊዜ አምሽቼ ስገባ ማመሽ ከሆነ ደውዬ እነግራቸዋለሁ ለይብራሪ ውስጥ ግን እነሱ ያመጡልኛል ሰዓቴን ጠብቀው ቤት ከሆንኩኝ ግን እኔ ራሴ ነኝ ምወስደው

ከልጆችሽ ፊት ስትወስጂ ያለፍራቻ ትወስጂያለሽ፡ ቤተሰብ ጋር ችግር የለም ፡

**ከለይብራሪስ፡** ውሃ ልጠጣ ብሎ መውጣት ነው

**ከመድሃኒት ብዛት ጋር በተያያዘ፡** አሁን ለይ አንድ ፍሬ ነው ምወስደው ድሮ ብዙ እወስድ ነበር የአሁነ በጣም የተሸለ ነው ከሰዓትም ፡ ከሐይማኖቴ አንፃርም ይመቸኛል፤ ምግብ ምናምንም አልልም ዝምብዬ እወስደዋለሁ እስከሁን ምንም አይነት ጫና አልደረሰብኝም

**በቀን ሁለቴ ስትወስጂ በነበርሽበት ገዜስ፡** የዚያን ጊዜ አለፆምም ነበር ፤ስለማይፈቅዱልኝ ከምግብ በሆለ ነው መድሃኒት ሲሰጡኝ የነበረ አሁን ግን ምንም አይነት ነገር አልበለም

**የጎንዮሽ ጉዳትስ አለ፡** አዲስ መድሃኒት ሲሆን አለው ፡ አዲስ ሲሆን ከመላመድ አንር ስላለው አለው ለምሳሌ፡ ሌሊት ሌሊት ስለምነሳ በአፍ ለይ ያለው ጠዕም/ቃና ለየት ያለ ነው እሱን ደሞ እኔ አልወደውም

**ሌሊት ምን ልታደርጊ ነዉ ምትነሺው፡** ጥናት አለ; ኢንተርቪው/ቃለ-መጠይቆችም በአብዛኛውም በውጮች ሌሊት ለይ ስለሆነ ለቃለ መጠይቅ ሌሊት ነበር ምነሰው

**አሁን ለይ ይህ ጠዕም/ቃና አለ፡** አሁን ለይ ለምጄዋለሁ የለም

**በተቆም ርቀት ጋር ተያይዞ ያሉት ነገሮች፡** ቅርብ ስለሆነ በእግሬ ነው ምመጠው

ሌላ ቦታ ሂደሽ ታውቂያለሽ፡ አዎ አብዘኛውን ከባህር-ዳር ውጭ እንሄደለን እና ለአለቆቻችን ነው ምንሰጠቸው ለህፃናት ሰይሆን ትልቅ ለሆኑ ለምሳሌ አዲስ አበባ ስሄድ አስረደሆቸውና በእቃ አድርጌ ለአለቃው እሰጠዋለሁምክኒያቱም ሌሎች ልጆች ማየት የለባቸውም ከዛ ሰዓቴን እነግራዋለሁ ሰዓቴን ጠብቆ ያመጣልኛል

ሌሎች እንዳያዩ ነው ለአለቃው ምትሰቸው፡ አዎ/ይጠፈብኛል በሚል ነው፤ ሌሎች ቢያዩ አይመለከተኝም የመስጠት ግዴታም አለብን

**ሆስፒታሉ እየሰጠ ካለው አገልግሎት አነፃር ክፍተት አለ፡** እዚህ መድሃኒት መውሰድ ከጀመርኩ አንድ አመት ከስድሰት ወር ሁኖኛል፡፡ ወዲያውኑ ነው ልጆች የተቀበሉኝ መጀመሪያ ለይ እነሱን ለመላመድ ተቸግሬ ነበር ፤ ከወንድሞቼ ጋር ብቻ ነበር ምሆነው ወንድሜ የእነሱ አለቃ ነው ግን ማነሳውን ሀሳቦች በጣም ነው የሚወዶቸው ጥያቄ ይጠይቃሉ ግዴታ መመለስ አለብኝ ከዛ ቀስበቀስ መለማመድ መጣ፤ አሁን ከእነሱ ጋር ልክ እንደ ወንድምና እህት ነን እዚ ጋ በምወጠበት ጊዜ ትናንትና አይተሃኝ ከሆነ ኖረማል ነው ማወራቸው ድፍረት የለኝም ነበር ቁጭ ብዬ ክፍል እንዳልኩህ ማንም ጋር አለዋራም መፃፍ ነበር የኔ ስራ፡፡

አሁን ለይ ግን ኮንፊደንስ አለኝ ሌሎች ፓርላማዎች ለይ እሳተፋለሁየአፍሪካ የህፃናት ቀን ለይ ወጥቼ የመነገር ክህሎት አለኝ እና እዚህ ድርጅት ለይ ምን አለ ብዙ ወንድም እና እህት ታፋራለህ አልፎ ተርፎም ምታገራው ብዙዎች ትልልቆች ናቸው በእድሜ ከኔ በለይ ናቸው ግን እነሱ መጠው ለኔ ልምድ ያካፍሉኛል ሰለም እምነት አለ ሌሎች ትምህርቶችንም እየሰጡ ነው ብዙ ነገሮችን እንማርበታለን እና አሪፍ ነገር ነው ያያዙት

**ከበለሙያስ ክፍተት አለ፡** እኔ አዲስ ስለሆነኩ ያን አለውቅም

**እየተሰጠ ያለው አገልግሎተስ፡** ለኔ አሪፍ ነው

**ከማህበረሰብ የደረሰብሽ መግለል/አድሎ አለ፡** ህፃን እያለሁ ማንም አያውቅም እኔ ለራሴ አለውቅም ግን ወንድሞችና እህቶቼ ለምን መድሃኒት እንደምወስድ ይጠይቆታል እናቴን እና ይመለከታችሆል ትለችዋለች፤ እኔም ስለማለውቅ ምንም አይመስለኝም በሆለ ለይ ግን እኔ ከወቅሁ በሆለ እነሱ ሲስሙ ምንም አልመሰለቸውም እነሱ የመሰለቸው እኔ ታምሜ ምተኛ ነበር ያሰበት ከዛ በሆለ ግን ምንም ነገር አለለችም

ከድርጅቱና ከቤተሰቤ በስተቀር ማንም ሚያውቅ የለም፡ እነዚህም አለደረሱብኝም ህዝቡም እየሰለጠን ነው ያለው አሁን ለይ

**ቨይረሱ ጋር ለሚኖሩ ወጣቶች ድጋፍ ያስፈልጋል ትያለሽ/ከማን**

እየተሠጠ ይገኛል እዚህ የተለየዩ ስልጠነዎች እኔ ሁለት ስልጠነዎች ለይ ልሳተፍ ነበር በአንደኛው ጋር ተሳትፌ በሁለተኛው ቃለ መጠይቅ ስለነበረብኝ ቀረሁ/ከፍታ፤ወጋገን/ሚሉ ስልጠናዎች ተሰጠዋል የተለያዩ ስልጠነዎች ይሠጣሉ

እየተሰጠ ያለው ስልጠና በቂ ነው ብለሽ ታስቢያለሽ፡ ዋና አለቆች ወንድሞች ስለሆኑ ምን ምን ስልጠነ እንደተሰጠ ይነግሩኛል እና እየተሰጠ ያለው ነገር አሪፍ ነው፡፡

**ከመንግስት በኩል እየተደረገ ያለው ድጋፍ በቂ ነው ትያለሽ፡** በቂ አይደለም በእርግት

ለምሳሌ በኢኮኖሚ ደረጀ አክመ ደካሞች አሉ ለእነሱ ሳሙና/የነፅህና መጠበቂያ/ ከመስጠት አልፎ ለእነሱ ሌሎች ነገርች ቢሞሉላቸው ብዬ አስባለሁ ለምሳሌ እንደ ልክ ቤት ውስጥ ማሞለት ከዚህ አገልግሎት በለፈ ስነ-ልቦናዊ የምክር አገልግሎት ቢሰጥ

**ከማህበረሰቡስ ምን ይጠበቃል፡** የምክር አገልግሎት ብቻ ቢሠጥ በቂ ነው ከእነሱ አልፎ ሁሉን ነገር ሚያደርጉልን ቤተሰቦችና ዶክተሮች ናቸው ከወቁ ደሞ ሚስጥር መጠበቅ ግዴታቸው ነው

**ሌላ መጨመር ምትፈልጊዉ ነገር ካለሽ፡** እኔ ቤተሰቦቼን ያጠሁዋቸው በቨይረሱ ምክኒያት ነው እነሱ ሊሄዱ የቻሉት በለመወቃቸው ነው ግን ማንም ሰው እናቴ እንዲህ ናት እናቴ ናት ያስያዘችኝ ብሎ ቤተሰቦችን ሊወቅስ አይችልም/አይገባም/ ይህ የእድል ጉደይ ነው ብዬ ነው ማምነውና ሰዎች ከተሰጠን አንፃር ነው እንጂ ራሳችን ፈልገን የምናማጠው አይደለም አንድ አንዶች ከማህበረሰቡ ጋር ያለው ነገር እኔ ተለቅ ብል ከማንም ጋር ስትልቀሰቀስ እኮ እነደዚህ አይነት ነገር ያጋጠማት ማለት የለባቸውም የጀርባ ታሪካችንንና የእኛን ምለሽ ሰያውቁ እናን ሊገመግሙ አይችሉም፡፡

**ተሳታፊ ስድስት፡**

**ከኤች አይቪ ጋር መኖር ላንቺ ምን ማለት እንደሆነ ግለፅ/አብራሪልኝ**

ከኤች አይቪ ጋር መኖር ያው በቃ ከጎደኛ ጋር አብሮ እንደመኖር ማለት ነው ማለትም ማድሃኒቱ አብረህ የምትወስደው ሁሌ ተላምደህ በየዕለቱ ምታደርገው ተግባር ስለሆነ ምንም የተለዬ ነገር የለም

**ቨይረሱ በደምሽ ውስጥ እንዳለ ስታስቢ ምን ይሰማሻል/ሚሰማሽ ስሜት አለ**

አንድ አንድ ጎደኞቼ የተለዬ ነገር ሲያደርጉ እዛኔ የሆነ ሚሰማኝ ነገር ይኖራል

**የተለዬ ስትይ ምን ምን**

ጎደኛ ሲኖረቸው/ፍቅረኛ ሲኖረቸው …እንዴ!…እኔ ለምንድነው ጎደኛ ማልይዘው ብዬ አስባለሁ ግን በሆላ ደሞ ከእነሱ የተሸልኩ ነኝ ብዬም አስባለሁ

**ጎደኛ እንዳትይዢ ያገደሽ ነገር አለ**

ሳይሁን የምትፈልገውን ነገር በምትፈልገው ሰዓት ለታደርግ ትችላለህ /በቨይረሱ ምክኒያት

**በቨይረሱ ምክኒያት ያጋጠሙ ፈተናዎች አሉሽ**

ያጋጠሙኝ ነገሮች፡ ብዙ ነገሮች አሉ በትንሹ ደሞ አንተ ሴት ስትሆን የሆነ ሚደርስብህ ጫናዎች አሉ ፤ተፈላጊ ትሆነለህ አስገድዶ መድፈር እነዚህ እነዚህ ያው ብዙ ወንዶች አንተን ተፈላጊ ያደርጉሃል/ፆታዊ ትንኮሳ ይደርስብሃል/…ፆታዊ ትንኮሳ/አስገድዶ የመድፈር ሙካራ ደርሶብኛል..እንደዛ ተደርጎ ግን እኔ ጠፍቼቸው አውቀለሁ..ሊያፍኑኝ ሁላ ሞክረው ነበር ግን አቅጣጫ ቀይሬ አመለትኮቸው፡፡

**ማን በምን አይነት ሁኔታ**

እኔ ጎደኛው እንድሆን ይፈልጋል እሱ እና ግን እኔ አልሆንህም ብዬ በጣም ብዙ ነገር ነግሬው ነበር እና ሊረዳኝ አልቻለም ፤ መናገርም አልፈለግሁም በወቅቱ ፤አልሆንህም አልኩት ምክኒያቱም የመነጋሪያ ጊዜና ሰዓቱ አይደላም ብዬ ስለሰብኩ ከዛ እኔ በላሰብኩበት ሰዓት ምሽት አካበቢ ነው እና ጎደኛው እዛጋ ነው ሌላ ቦታ እና እኔን አየተከታተለኝ ነበር ለካ ወደ ሰፈር አካበቢ ልገባ ስል አፍኖ ያዘኝና እዛ እንደምንም ብዬ ራሴን ተቆጠጥሬ በቃ ማድረግ ትቺያለሽ ብዬ ራሴን አበረታተሁና ከዛ ውስጥ ወጠሁ /አመለጥኩ /..ይህ ነው ትልቁ ፈተናዬ

ትንሹ ምለው ……….እኔ እንጃ እንግዲ.. እህህህ..በአካባቢ ሚያወሩት ወሬ**፡**

**ምን አይነት ወሬ፡** ማለት ሰዎች እየሄዱ እያሉ አዚህ ሆስፒታል ቢያገኙህ አንተ ምን ብለህ ትመልስለቸዋለህ..መቼስ መድሃኒት ልወስድ ነው ብለህ አትመልስላቸውም ሰው ልጠይቅ ነው ትለቸዋለህ ..ዘመድ ነው የታመመ ይሉሃል..እነሱ ደሞ አዎ ዘመድ ነው የታመመ ብለህ፡ የቅርብ ሰው ነው ወይስ አይደላም ብለው ይመልሱልሃል መልሰው እነሱ ከዛ አንተን ፊትህን አይተው ለመረዳት ምናምን ይሞክራሉ ግን ደሞ አንተ ያን ሁሉ ጫና እልፍ አድርገህ እንደ ቀልድ አድርገህ ታልፋቸዋለህ በቀልድ እያዋዘህ ማለት ነው …ትንሹ ፈተና ይህ ነው

**ሌላስ**…..የለም

**ቨይረሱ በደምሽ እንደላሽ ማን ነገረሽ /እንዴት አወቅሽ**

ያወቅሁት ራሴ ነኝ ማለት ቤተሰብ ነው ሚያሰጠቅመኝ መድሃኒት በእርግት ግን ደሞ እኔ እዚህ ስመጣ ቤተሰቦቼ ጉንፋን ነው ይሉኛል ጉንፋን አይለቀኝም ነበር ከዛ አንድ ቀን ለክትትል መጥቼ ኤችአይቪ እያሉ ያወራሉ ሰዎች ፡ እዚህ አብረን ሚወስዱ ጎረቤቶች አሉ ሲወስዱ ኤች አይቪ ምናምን ሲሉ ሰማሁዋቸው ኤችአይቪን ማውቀው በትምህርት ደረጀ ነው እንጂ እንደዚህ ይይዘኛል ብዬ አስቤ አለውቅም እዛ መድሃኒት ካርዴ ጋር ኦኤች ተብሎ ተፅፎል ያንን ሳይ ለካ በምፀሀራ ቃል (ሳቅ/ሃሃሃአአአ)

ዶክተሩዋን አንድ ቀን ጠየቅሆት፤ ይህ ምንድነው ሲስተር ስላት፡ ይህ ኤችአይቪ ማለት ነው አለችኝ እነዴ! እኔ ታዲያ የኤችአይቪ ተጠቃሚ ነኝ ስላት፡ አዎዎ፤ እነዴት አታውቂም አለችኝ ቤተሰቦቼ ሲያመጡኝ የጉንፋን መድሃኒት ልትወስጂ ነው ሚሉኝ አልኮት ፡ ከዛ ሲስተርዋ ችግር የለውም ይህ መድሃኒት ስላለው ችግር የለውም የሚል ምላሽ ነው የሰጠችኝ በወቅቱ እኔም እነዳልደነግት ስለፈለገች ሊሆን ይችላል በዘውም ደስ ሚል አጋጣሚ ነው አስደግቶኛልም፤

**ደንግተሽ ነበር፡** እንዴ እኔ ይኖርብኛል ብዬ አለሰብኩም የሰማሁትም ስድስት/ሰባት አመት ነበር፤

**ስትሰሚ** ምን ተሰማሽ፡ የተሰማኝ ደስታም ሀዘንም ተሰምቶኛል፤ እየሳቅሁ አለቅሰለሁ የደስታም የመቀፈትም ነው ፡ እንደዚህ ይጎደኛል ብዬም አለሰብኩም እኔንም ይይዘኛል ብዬ አለሰብኩም ፡ አልጠበቁትም ጭራሽ ፤ በወቅቱ እንግዲህ ቢያስለቅሰኝም ቢያሰዝነኝም ግን እንዴት ከኔ ጋ መጣ ብዬ ጠየቅሁ

**ማንን** **ጠየቅሽ፡** አባቴን እዛ ማን ነገረሽ አለ፡ አይ ዶክተሮን ጠይቂያት እኮ እንደዚ እንደዚ አለችኝ ስለው ይህ እኮ እና ችግር የለውም እኛም ምንወስደው መድሃኒት እንደዛ ነው አለኝ ፤ለካ በሽታው የመጣው ከእናት ወደ ልጅ ነው እድሜ ልኬን ስወስደው የነበረ ማለት ነው እናቴንም በዚሁ አጋጣሚ ነው ያጣሆት እኔን ከወለደች በሆለ ሞተች፤ እዛ ብዙ ህይወት ማሳጣት ስላለ መድሃኒትሸን መቆረት የለብሽም ብሎ አባቴ ራሱ ያረጋገ ነበረ፤ ያው ቤተሰብ ጋር ደስ ሚል ህይወት ይኖራሃል ፤ በዚህ ጉደይ ለብዙ ጊዜ ሀዘን ውስጥ ነበርኩ በየቀኑ አለቅስ ነበረ ከዛ አባቴ አንድ ቀን አየኝና ለምንድነው ምታለቅሺ አለኝ ፤ አባቴ ለምን ይህን መድሃኒት እወስደለሁ ጎደኛዬ እኮ አተወስድም አልኩት፤ ከዛ ይህ እኮ ችግር የለውም መድሃኒት ብትወስጂም አንቺ የተሻለ ሕይወት ትኖሪያለሽ አለ እንደማንኛውም ሰው ተምረሽ ሰርተሽ ሕይወት ኑሮሽ በቃ ማንኛውም ሰው ሚያረገውን ነገር ታረጊያለሽ አለኝ ይበልጡን እንደዚህ ሲል ደስ አለኝ ምክኒያቱም ሁሉም ነገር ማድረግ እንደምችል አወቅሁ

ከዚህ በፊት መደሃኒት ያልተገኘለት በሽታ ተብለን ነው የተማርን እንደዛ ስለሆነ ሚገድለኝ ሁሉ ነው የመሰለኝ ፤ይህ ሁሉ ያሰጨንቀኝ ነበር፤ ከአባቴ በተጨማሪ እህቴም ጎደኞቼም ከጎኔ ነበሩ፤ በጣም ምትቀርበኝ ጎደኛዬ አለችኝ የመድሃኒት ሰዓት እንዳላሰልፍ ደውላ ውሰጂ ትለኛለች ሰዓት አለፎብሻል ነይ ግቢ ትለኛለች ጎረቤትም እነደዛው ደውለው ሁለ ይነግሩኛል አለዚያ የሆነ ኮድ ነገር አለ መጠረሪያ ይጠሩኛል

**ምን አይነት ኮድ ነው ምትጠቀሙት፡** ኮዱ ፉጨት ሊሆን ይችላል ሲያፎጩልኝ እገባለሁ/ገብቼ ምድሃኒት እወስደለሁ/፤ሰዓት እነደያልፍብኝ ማለት ነው

**ለማንኛውም ሰው /ለጎደኞችሽ ከቨይረሱ ጋር እነደምትኖሪ ነግራቸዋል፡** ለማንኛዉም ሰው ሳይሆን ለአንዷ ጎደኛዬ ነው የነገርኋት

**ለምን መረጥሺያት፡** አብረን ስለአደግን **፤** አብረኝ ከኔ ጋር ናት እሷ

ከሰማች በኋለ ምን አይነት ምላሽ ነበረት፡ እናቷ ስለምትጠቀም በእርግት እሷም ምንም ችግር የለበትም ፤ ከነገርኋት በኋለ ይበልጡን ነው ምትወደኝ አብዝታ እኔም እወደታለሁ

ለሌሎችስ ለምን አልነገርሺያቸውም ፡ እንዴ ለምንድነውማ ይሄውልህ በጣም ብዙ ነገሮች ሊደርሱብህ ይችላሉ ፡፡ትምህርት ቤት ለይ እየተማርክ አንተ ወደ ትምህርት ታስብ ወይስ ልጆች ምን እያሰቡ ነው ብለህ ታስብ ይህ ትምህርት ለይ እና አምዕሮን ይረብሻል ፡ የትምህርት ጎደኞቼ ቢያውቁ በሽኩሽኩታ ቢያወሩ ራሱ ስለ እኔ ሚያወሩኝ ነው ሚመስለኝ

**ሌላ የለም**

**በአሁኑ ሰዓት የወንድ ጎደኛ አለሽ፤** ጎደኛ አለኝ በእርግት እዚህ አብሮ ነው ያለው ራሱንም ያውቃል ሚሰጠውን ትምህርትም አብረን ነው ምንወስደው

የግብራ ስጋ ግንኙነት ጀምራችሁል፡ አልጀመርንም

ስለ ኤችአይቪ ስርጭትና መተላለፊያ መንገዶችስ ውይይት ታረገለችሁ ፤ እዚህ ተምረን ስለምንሄድ እናውቃለን፡፡

ስለወደፊቱ ህይወት በተለይ ከቨይረሱ ነፃ የሆነ ልጅ ለመውለድ ምን አይነት ጥንቃቄ ተራጊያለሽ፤ እሱን ለወደፊቱ በለሙያ ምናመክረው ነው ሚሆነው

ስለወደፊት ህይወት ግን ደስተኛ የሆነ ሕይወት እንዲኖረን ነው ፍለጎታችን

**ስለመድሃኒት አወሳሰድን በተመለከተ፤** ሁሌ ነው ሚወሰድ እና ሰዓቱ እንደያልፍ በአለርም ነው ምንቀሰቀሰው ፤ አለርም ሲጮህ ቤት ገብቼ እወስደለሁ

የክኒኑን ብዛት በተመለከተ፡ ማታሁለት ክኒን ነው ምወስደው ፆምም እፆማለሁ ብዛቱም አያስቸግረኝም

ከሰው ፊት የመውሰድ ፍራጫ እንዴት ነው፡ ከሰዎች ፊት አልወስድም ፤ መድሃኒት ምወስደው ከክፍሌ ውስጥ ገብቼ ነው ድንገት በት ውስጥ/ክፍል ውስጥ እነግዳ ካለ /የሌላ በሽታ ነው ብዬ እናግረቸዋለሁ ፤ለምሳሌ፤የጉንፋን መድሃኒት ነው እላለሁ

የመድሃኒት የጎንዮሽ **ጉዳትስ፡** ሳትወስድ ስትቀር ነው እንጂ ሚምህ ሌላ የለም አንድ አንድ ሰዎች ለይ አይስማመም በኔ በኩል ችግር የለም

የተቆም ርቀትስ፡ ከትራንስፖርት አንፃር፤ ይህን ያህል አያስቸግርም ፤አንዳንዴ ከቤተሰብ ስትቀበል ትንሽ ሊደብርህ ይችላል ፤ግን እዚህ ለታክሲ/ትራንስፖርት ሚሆን ይሰጣናል፡፡

**ለወጣቶቹ ማን ምን አይነት ድጋፍ ቢያደርግ ጥሩ ነው ትያለሽ፡**

ለወጣቱ የተለያዩ የስራ እድል ቢፈጠርላቸው አሪፍ ነው፤ ማለትም አጫጭር ኮርሶች ቢኖሩ በትምህርትም ሆነ በሙያም ስልጠና ሊሆን ይችላል በእነዚህ የተውሰነ ድጋፍ ቢደረግ ቢያንስ ስራ አጦ ከሚቀመጥ ሰው የተወሰነ የእጅ ማፍታቻ ቢኖረው ቢያንስ የታክሲም እንኳ ይቸግራል ትንሽም ነገር ተደርጎ ይበልጥ የተሸለ ነገር ይኖራዋል

ሌላው የትምህርት እድል ያለገኙ ልጆች እዚህ መጠው ቢያገኙና ተጠቃሚ ቢሆኑ የመድሃኒት እጥረት ከለም መድሃኒት እንዲያገኙ ማድረግ፡ ከሌላ ሀገር ከክፍለ-ሀገር ሚመጡ ልጆች አሉ ለእነሱ በቂ የሆነ የትራንስፖርት ክፍያ ቢሰጠቸው ለምሳሌ ከደብረ-ታቦር ሚመጡ አሉ ለእነሱ በቂ የሆነ ክፍያ ቢሰጥ አሪፍ ነው

ከአገልግሎት አሰጣጥ ዙሪያ፡ እዚህ ሚሰጠው አገልግሎት በጣም አሪፍ ነው በለሙያዎችም እንደ እህት እንደ ወንድምና እንደ እናት ነው ሚንካበከቡን ፤ እርግት አንድአንድ አለማግባበቶችም አሉ ግን ይህንን በራሰችን ሚፈቱት ነገር ሊኖር ይችላል እነሱም ሚፈቱት ነገር ሊኖር ይችላል አንዳችን ለአንዳችን እየተሳሰብን እነፈጣዋለን

ከማህበረሰብ ማግለልና መድሎ የደረሰብሽ ነገር አለ፤ በአርግት

ድሮ ለይ በሰፊው ይታይ ነበር ለማህበረሰቡ ትምህርት ስላልደረሰው ቢያንስ ብትነከቸው እንኳ ሚተላልፍባቸው ነው ሚመስለቸው ግን አሁን ለይ ትምህርት እየተስጠቸው ስለሆነ አሁን ለይ ተቀርፎል፡፡ አሁን እኮ ይህ እኮ ጉንፋን ነው ኤችአይቪ ማለት ጉንፋን ነው እኛን ሚያስፋረው እንደ ሰኩወር ና ግፊት አንዴ ፀጥ ሚያደርጉ፡ ማስፋራት ያለባቸው እነዚህ በሽታዎች ናቸው እንጂ የኛ ኖርማል ነው፤ አሁን እኔ ያለብኝ አልመስልም በነፃነት እንደሌላው እወጣለሁ እገባለሁ በዛ ለይ ያው እንግዲ ወጣት ነኝ መስራት እችላለሁ፡

የማህበረሰቡ አመለካከት ትንሽ ይቀረቸዋል የመግለል በህርይ አለ በተለይ የተማረም አለ የተማረ አካል ነው ሚያጠፈው እኔ ለራሴ ከወቅሁ ደበቅ ማድረግ ነው እንጂ ያንን ስለወቅሁ ማውራት የለብኝም እኛ ወጣቶችም እናጠፋለን በርግጥ እድሜም ሚመክረቸው ሰዎች አሉ ወደ ቦታ እንድንመልስ ያደርጉናል

**ቨይረሱ ካንቺ ወደ ሌላ እንደይተላለፍ ምታደርጊው ጥንቃቄ አለ፡** መወሰን ሌላ ደግሞ መከለከያ መንገዶችን መጠቀም ፤ መስተማር ፤ እኔ ከደማሁ ሰዎች እንዲተጉ አለፈልግም ምክኒያቱም ያ ሰው ደሙን አይቶ እንዲሸሽም ደሙን እንዲነካም አልፈልግም ፤ ግን ሰው ነህና በአጋጣሚ አደጋ ሊፈጠር ይችላል ..በዚህ ሰዓት ጥንቃቄ ማድረግ በአጠገብ ያሉ ሰዎችም ካሉ ሊነግሩት ይችላል፡፡ ሌላው በበጀጅ ሲሄዱ ሴቶች ተጋደው ሊደፈሩ ይችላሉ ማለት ያቺ ሴት ምን እንዳለበት አትተወቅም ምን እንደላበት ለምን ይደፍራታል ያም ያለፍለጎቷ ፤ያለፍለጎት የሆነ ነገር ፈፃሜውም አያምርም እሷም ተጎጂ ትሆነለች ፤ሁለታቸውም ህይወታቸውን አሰልፈው ሰጡ ማለት ነው ..ይህን ለበለበጀጆች ነው ማስተላለፍ ምፈልግ …በጀጆች ከዚህ ድርጊት ቢቆጠቡ

**ድጋፍን በተመለከተ፡**

በበጀት ድጋፍ ቢደረግ ፤ማህበሩ ራሱን ችሎ እነቅስቃሴ እንዲያደርግ የሆነ ካምፓኒ/ድርጅት ሚንቀሰቀስበት ነገር ቢኖር አሪፍ ነው

**ተሳታፊ ሰባት፡**

**ከኤች አይቪ ቨይረስ ጋር አብሮ መኖርን እንዴት ትገልፂዋለሽ**

እሺ ኤች አይቪ በራሱ ጊዜ የራሱ የሆነ ችግር አለ:: ማለት ተመስገን ነው; ከድሮ ማህበረሰቡ ለይ ያለው አድሎና መገለል እየቀነሰ ነው:: ግን ሙሉ ለሙሉ አልጠፋም::

እእአ ችግሮችም አሉ፡፡

ማህበረሰብ ለይ በትምህርት ደረጀ ስናወራ ክለስ ለይ ስንማር የሆነ ባይሎጂ አካባቢ ሚመጣበት ርዕስ አለ እዛን ጊዜ ደሞ ተማሪው ኤች አይቪ ያለበትን ሰው እንደ ሰው መይቆጥሩበት አጋጣሚ አለ ከጎደኛዬ ጀምሮ ፤ ሰሞኑን ጎደኛዬ ኤችአይቪ ቢኖርብኝ ራሴን ማታፋ ይመስለኛል ብለኛለች ፤ እኔም ይህንን ተረድቼ ለማብራረት ሞክሬ ነበር ፤ ኤች አይቪ ከግፊት ከስኩዋር ከካንሳር ሚለይበት መድሃኒት በየቀኑ መውሰድ ይህ ልክ እንደ ግፊት ነው እና ኤች አይቪ አንድ ችግር ነው ብዬ ማስበው ከሰው ወደ ሰው መታላለፉ ብቻ ነው ያ ነገር ደሞ ፈጣሪ አንዴ ስለሰጠ መቀየር አይቻልም እና ሌላ የተለየ ነገር ራስን እስከማትፋት ሚያደርሰው ነገር ያለ አይመስለኝ ብዬ ስከረከራት በይ ተይዉ ብለ ዘገነው፤፤ማህበረሰቡ ለይ ትልቅ ችግር አለ፡ ምን ልበልህ በጣም ከበድ ነው ብዙ ይቀራል፡፡

**ስለ ኤች አይቪ ስታስቢ ምን ይሰመሻል፡** መቼም ደስታኛ ነኝ አልልህም ምክኒያቱም ስለልሆንኩ ግን ያው ተቀብዬም መድሃኒት በመውሰድ እየኖርኩ ነው፡፡

**ደስተኛ እንዳትሆኚ ያደረገሽ ምክኒያት ምንድነው፡** ቨይረስ በደሜ ውስጥ በመኖሩ፡

ምን ምን ችግር ደረሰብሽ ፡ በሞራል ለይ ማለት በአብዘኛው ጊዜ ሁሌም እንደዚህ አይነት ሳስብ ትዝ ሚለኝ በየክለሱ ኤችአይቪ ርዕስ በባይሎጂ መጽሐፍ ለይ ሁሌም አለ ፤ያንን መምህሩ እንደ አይነቱ አገላለፅ ይገልፃዋል ፤ መምህሩ ሲገልፅልን አንዱ ጥሩ አድርጎ ሲገልፅልን አንዱ ደግሞ ኤችአይቪ ሚበለውን ነገር ጭራሽ እንዳነስብ እና መጥፎ እንደሆነ ነገር ና አንተን የሚጎዳ ነገር ይናገራል ከመምህራን ያ ችግር አለ ያን ስትሰማ ደሞ አንዳንዴ ስሜት ይሰማል/ የስነ-ልቦነ ችግር/ጫና/ አለ/፡፡

**ያጋጠሙሽ ፈተና፡ መድሃኒት፤**የሆነ ፕሮግራም ነበረን ከ ጎደኞቼ ጋር እና መድሃኒት እእእ..የእነሱን እቃ በኔ ቦርሳ ያራጋሉ ስለዚህም የኔን ቦርሳ እንደፈለጉ ይወስደሉ ይህ ደሞ መድሃኒት ይዞ ለመሄድ በጣም ከበድ ፈተና ነው፡፡ **መጠጥም አልጠጣም ስትለቸው የሆነ ፋራ ምናምን ይሉሃል፡፡ ማለት ሌላ ነገር ጋር ያጋነኙብሃል በዚህ ሰዓት አዕምሮህ ይጨነቀል**

**ራስሽን ለሌላ ሰው አሰውቀሻል ፡ የለም አላሰወቁም ፤ ቤተሰብም ያውቃሉ እነሱ ጋር ግልፅ ነኝ ሌላ ቦታ ለጎደኞቼ/ዬ እንደምነግርህ እኔ መኖሬን በራሱ ብነግራት እንደምትርቀኝ ስለሚሰመኝ እና ማንም ፤ከብዙ ጎደኞቼ ያየሁት ስለ ኤች አይቪ ያለቸው አመለካከት የወረደ ስለሆነ ራሴን መደበቅ አስፈላጊ ሁኖ አግኝቼዋለሁ፡፡ ለዛ ነው ዝም ያልኩት….**

**ቨይረሱ እንዳለብሽ ያወቅሽው መቼ ነው፡** ያው ማለት እኔ እንኳ እነሱ ነግረውኝ ሳይሆን ፖስተር ፤ኤክስ ምልክት፤ ታያለህ፤ ብዙ ነገር ታነበለህ፡፡ ራሴ ነኝ ያወቅሁት ፤አውቃለሁ ግን ዶከትሩ የምን መድሃኒት እንደምትስወጂ ታውቂያለሽ ሲሉኝ፤ አዎ የኤችአይቪ ነው ብየቸዋለሁ ለኔ ማንም አልነገረኝም እዚህ ስመጣ ፖስተርም አለ ሁሉ ነገር አለ፤ታያለህ ታነበለህ ሁሌ እንደምትወስደው በትምርቱ ለይ አለ **በዚህ ሁኔታ ነው ያወቅሁት**

**ካወቅሽ በኋለ የተሰማሽ ስሜት/ሁኔታ ምን ነበር ፡** ማለት በፊት ልጅ እያለሁ ምንም አይሰመኝም ነበር ከፍ ስትል ነው ነገር ሚመጣ ነው እንጂ ድሮ ምንም አይሰመኝም አሁንም ማህበሩ እስካለ ድረስ ምንም አይሰመኝም እግዚአብሔር የተመሰገነ ይሁን፡፡ ማህበሩ በይኖር ኑሮ እዚህ ግቢ ምመጣ አይመስለኝም ምክኒያቱም እኔ ብቻ ስለሚመስለኝ አሁን ግን ይህ ማህበር ከተመሰረተ በኋለ ብዙ ጎደኛ ታፈራለህ ያው የአምዕሮ ነፃነት ታጋኛለህ

ለመጀመሪያ ጊዜ እንደሰማሁም የልጅነት ጊዜም ስለሆነ ከቤትም ይህን ያህል እንዳለስብ ያረጉኛል፤ ሰፈር ለይ የሆነ ህጸን እያለሁ ጨዋታ ስንጨወት ኤችአይቪ አለበት እኮ ለምንድነው አብረችሁ ምትጨወቱት ሲሉ ያኔ ብቻ ነው በልጅነት የተሰማኝ ከዛበኋለ እንኮ እናቴም እያረሳሰች ምንም እያለች ሚገርምህ ሱቅ ለራሱ ልገዛ ዕቃ እያየሁት የለም አልሰጥም የተባልኩበት አጋጣሚ ሁሉ ነበር

እንደዚህ በሚሉበት ሰዓት ምን ተሰማሽ፡ ማለት ሰው ነህና አልቅሼለሁ ሁላ ከዚህ በለይ ምንም ህይወት ይቀጥላል …ያው ከማህበረሰቡ ለይ መግለል ና መድሎ አልቀረም

በቨይረሱ ምክኒያት የደረሰብሽ ጫና/ተፅዕኖ/ አለ፡ በትምህርት ለይ የለም

**የወንድ ጎደኛ/ፍቅረኛ አለሽ፡** የለም ለወደፊቱስ ፡አሁን ለይ ትምህርት ለይም ስለሆንኩ አለስብም ለወደፊቱ ግን መቼም ሰው ነኝና ያን ነገር አልፈልግም ማለት አይደለም ማለት ጊዜውን ጠብቆ ቢሆን ጥሩ ነዉ አሁን ለይ እንደዚህ አይነት ሀሳብ የለኝም ግን ጊዤውን ጠብቆ ሁሉም ነገር ቢሆን ደስ ይላል፡፡ ለወደፊቱ ምን አይነት ባል እንዲኖርሽ ነው ምትፈልጊው/ቨይረስ ያለበት ወይስ የሌለበት፡ የሌለበት መሆን አይችልም ምክኒያቱም ከህሊና ዕዳ ነፃ መሆን ስለማትችል ማለት እሱ እሺ ቢል እንኳ በጣም ከበድ ነው ያን ሰው እኔ የሆንኩትን ጉዳት እንዲሆን ማድረግ ከበድ ነው ስለዚህ ያለህ አመራጭ እንደ እኔ ያለ ሰው ቢገኝ፡፡

ለወደፊት ልጅ እንዲኖርሽ ትፈልጊያለሽ ፡ አዎ …መቼም አልፈልግም ብል አንተም አታምንም

ከቨይረሱ ነፃ የሆነ ልጅ እንዲኖርሽና የእርግዝና መከለከያ ዘዴዎችስ /ጥንቃቄ

ሰዓቱ ለይ ስደርስ ዶክተሮችን ማመከር

**ስለ መድሃኒት አወሳስድን በተመለከተ፡** ያለበት ሁኔታ አሁን በጣም ጥሩ ነው ከድሮ በፊት የሆነ በተለይ ፆም ሲሆን የሆነ ጎደኛ ቤተክርስቲያን እናስቀድስ ሲሉ ጠዋት በልተህ መድሃኒት ስትወስድ ምን ሁሌ አንቺ አትፆሚም እንዴ ብለው የሆነ ነገር ነው ምትባል…በዛ ሰአት ፈጣሪ እንዲያው አንዴ ሚወሰድ መድሃኒት በሆነልኝ ብዬ ራሱ አውቃለሁ..አሁን የተመሰገነ ይሁን አንዴ ሚወሰድ መጣ ከዘም ሁሌ ጎደኞቼ ጋር እፆማለሁ …ቆይቷል አንድ አመት ሁኖል

**መድሃኒትን ያለ ፍራጫ ከሰዎች ፊት የመውሰድ ሁኔታን በተመለከተ፡**

ከቤተሰብ ፊት ምንም ችግር የለም ሁሉም ስለሚያውቁ ውጭ ለይ ግን በጥንቃቄ ነው ምወስደው ፡፡ ምን አይነት ጥንቃቄ ታረጊያለሽ ፡ ማለት ላስቲክ አለች የመድሃኒት መያዠ በወረቀት ከጥቅም ውጭ ስለሚሆን ያቺ ላስተክም ሙቀትና ቅዝቀዜን ትከለከለላች ያንም ከዘ ወስጥ አደርገለሁ ከዘ ሰዓቱ ሲደርስ ወጣ ብዬ እወስደለሁ

ከመድሃኒቱ ጋር የተያያዘ የጎንዮሽ ጉዳት አለ፡ ጉዳት የለም

**ከተቋም ርቀት ጋር በተያያዘ ፡ይህን ያህል አያስቸግረኝም**

ከማህበረሰቡ ሚታየው አመለካከት እንዴት ነው ፡ማህበረሰቡ ለይ እንደ ድሮ አይደለም አሁን እንኳ የለም ግን ትምህርት ቤት ለይ እንዳለብኝ ቢያውቁ በጣም ከበድ ነው ለምሳሌ ኤችአይቪ ስም ራሱ ሲጠራ ያስጣላል ብዙ ነገር አለ ከጎደኞች ጋር ብዙ ሰአት ስታሰልፍ ደሞ ብዙ አይነት ወሬ ሲወራ ወይ ተነስቼ እሄደለሁ ወይም እስከመጨረሻ አዳምጥና አንዳንዴ መልስ እሰጣለሁ ምክኒያቱም እኔ እየኖርኩበትም ስለሆነ ሁሉን ነገርም ስለማውቀው እና በጣም እንጃ ከበድ በጣም ፡ አንዷ እንዲያውም እናቴ ኤች አይቪ ያለበትን ሰው ሰለም እንደትይው ብለኛለች አለች

**ማን ምን ማድረግ አለበት ትያለሽ፡** የስራ ሁኔታ ቢመቻች ማለት ድርጅት ምናምን እንዲከፈት ቢደረግ እኔ ትምህርት ስጨርስ የሆነ እዚሁ ሀገረችን ምሰራበት ቢኖር ጥሩ ነዉ፡፡

ሁለተኛ ደሞ ችግር ነው ብዬ ማስበው ፡ እዚህ ጋ ስንገባ ኤ አር ቲ ሚለው ፖስተር ቢከላ /ፁሑፍ በይኖር ወይም ዞር በለው ቦታ ቢሆን/ ዘበኞችም ሌሎችም ስንገባ ቱክ ቱክ ብለው ያያሉ ..ወይ ደበቅ ያለ ቦታ ቢሆን ምክኒያቱም እኔ አንድንዴ ስገባ ጥበቆች ራሱ ያያሉ የሆነ ኤአርቲ ሚል ስለለ ይገበቸዋል ሁሉም የተማሩ ስለሆኑ የኤችአይቪ መድኃኒት ለመውሰድ እንደምንመጣ ይታወቃል ያን ጊዜ ብም ደስ ኤልም ..ምቾትም አይሰጥም

**ምን አይነት ቦታ ቢሆን ይመራጣል፡** ማለት ከሆስፒታል ወስጥ ሁኖም ዞር ያለ ቦታ አለ ዘበኞች ፤ተኝቶ ሚተከም ሰው በመይደርስበት ቦታ ቢሆን ጥሩ ነው …እዚህ ግን የሰፈር ሰውም ልታገኝ ትችላለህ ..ይህ ደም ፖስተርም ስለለ አንበው ሊረዱ ይችለሉ

**በቨይረሱ ሚደርሱብሽን ጫናዎችን ለመቋቋም ምን አይነት ዘዴ ትጠቀሚያለሽ**

በሰዓቱ ስሜታዊ እሆነለሁ ማለት ሁሌ ጸሎት ነው ማደርገው ፤በሽታ የለም አለ ለማለት ይከብዳል አንደንዴም ትረሳዋለህ ከወር አንዴ እዚህ መጣህ ይህን ቃል ስትሰማ ማለት ሁሌም ትምህርት ቤት ጎደኞች ጋር ስትውል ቨይረሱ ሲነሳ እና ሲዋራ ትዝ ሲልህ ያኔ ነው ያንተ ሚሰማህ ፡ የለም ብዬም አስባለሁ ያልኩት የሆነ ሰአት ታምሜ በተኘሁበት ሰአት ከዚህ ጋር ሚሄድ መድሃኒት ስጠኝ ብዬ ሳመክረው ዶክተሩን በልጅነትሽ ነው የጀመርሽ አለኝ ፤ አዎ አሁን ለይ እኮ ሊጠፋ ይችላል ምናምን አለኝ ሲመረምረኝ ውጤቱ የለብሽም ይላል እስኪ ታይው ምናምን አለኝ እዛኔም ደስም አለኝ በአንድም ደሞ እንደማይተፋም እሰማለሁ ሁለት ግማሽ ስትሆን ከበድ ነው ..እዚህ ደሞ ስሰማ ውጤቱ እንደዚህ ሊል ይችላል እርግተኛ መሆን አትችሉም አሉኝ፡፡ እኔም እንደላብኝ አምኜ ተቀበልኩ፤

እኔን ማያውቅ ሰው ቤት ውስጥ ስለ ኤች ኤይቪ ሲያወሩ/ወሬ ሲነሳ ወጥቼ እሄደለሁ

በቴሌቭዥን ስለ ኤች አይቪ ሚታይ ፊልም ይታያል ማየትም አልፈልግም አሰልፈዋለሁ

**ተሳታፊ፡ 11**

ኤችአይቪ ጋር መኖር ማለት ምን እንደሆነ ግልፅ ብታደርጊልኝ

ከኤችአይቪ ጋር መኖር ማለት ለኔ ደስታኛ ነኝ ምክኒያቱም እኔ ስለላመጣሁት ምንም አይመስለኝም ፡፡ ቤተሰብ ጋርም ሰለም ነው እስከሁን ድረስ ምንም የደረሰብኝ ነገር የለም እእእ..ማህበረሰቡም ጋም እንደዘ…ቨየረሱ ጋር መኖረ ምንም የተለየ ነግር የለም

በቨይረሱ ምክኒያት ሚሰማሽ ስሜት አለ

አዎ አንዳንዴ ስለ ኤች አይቪ ሁኔታ ሳስብ፤ ጎደኞቼ ሌላ ቦታ ለማዝነናት ሲሄዱ እኔ እንደ እነሱ ማልሆን ነበር ሚመስለኝ በፊት ለይ ምክኒያቱም ኤችአይቪ ኤድስ አለብኝ ብዬ ስለማስብ ፤እኔ እንጃ እንደዛ መደርግ አይመስለኝም ነበር ከማንኛውም ሰው ጋር እኩል እንደሆንኩ አምናለሁ፡፡ በዚህ ምክኒያት ትምህርት ቤትም አልሄድም ነበር፡፡ ማለት የሆነች ጎደኛዬ ነበረችና በጣም ነበር ምታሰቅቀኝ ማለቴ አንቺ ተጠቀሚ ነሽ ስለምትል ብዙ ጊዜ ክላስ አልሄድም ነበር …ሁሉም የሰማ ነው ሚመስለኝ እና ይህንን ፈርቼ ትምህርት ቤተ አልሄድም፡፡ እኔን ብቻ ያያዘኝ ነበር ሚመስለኝግን አደለም በሁሉም ለይ ሳየውም ምንም አደለም

በቨይረሱ ምክኒያት ያጋጠሙ ፈተናዎች አሉሽ፡ የለኝም

ግን የሆነ ሰአት ለስድስት ወር መድሃኒት አቋርጨለሁ

ከቤተሰብ ጋር ተገጭቼ አቋረጥኩ ከስድስት ወር በኋለ ተደወለልኝና መጥቼ መድሃኒት ጀመርኩ ስጀምር እጄን አመመኝ ሰውነቴ የመቁሰል በህርይ አመጣ ይህ ሁሉ የደረሰብኝ መድሃኒት በመቋረጥ ምክኒያት ነው፡፡ ይህ ነው በቨይረሱ ምክኒያት የደረሰብኝ ጫና

በደምሽ ቨይረስ እንዳለብሽ ማን ነገረሽ ፡ አባቴ በ11 አመቴ ነገረኝ

እዚህ መጥቼ የምን መድሃኒት ነው ምወስደው ብዬ ስጠይቀቸው ወባ ያምሻል ነው ሚሉኝ ከዛ ዶክተሩ እንዲነገራት ነገራት ከዛ ዶክተሮም ከስኩዋርና ከኤችአይቪ ከሚይዝሽ የቱ ይሻልሻል አለችኝ…እኔ ስኩዋር ምናምን አለውቅም እኔ እንጃ አልኳት…ከዘ አንቺ ያለብሽ ኤች አይቪ ነው ይህ ደሞ ምንም ማለት አደለም ከእሷ ከሰመሁ በኋለ ደሞ አባቴም ነገረኝ

በደምሽ ወስጥ ኤችአይቪ ቨይረስ አለብሽ ሲሉሽ ምን ተሰማሽ…ደነገትኩ/ድንጋቴ ተሰማኝ/ ከዘ የሆነ አለቀስኩኝ ከዘ አንዲት ጎደኛዬ ምንም ማለት አደለም ነገር አለችኝ

ማን ነግሮት፡ እንጃ ከቤተሰብ ነው መሰለኝ፤ እኔ ሳለውቅ እሷ ታውቃለች

ከዛ በኋለ መድሃኒትም አለቋርጥም

ራስሽን ለሌላ አሳውቀሻል፡ በእኛ ሰፈር ያሉ ሁሉም አንድበንድ ያውቃሉ

እኔ ነኝ የነገርሁቸው ምክኒያቱም ከእኔ ብዙ ነገር ስለሚዋሱ እንደይተላልፍበቸው ጥንቃቄ እንዲያደርጉ ብዬ ነው

ከሳወቅሽያቸው በኋለ ምን አይነት በህርይ አሳዩሽ ፡ አለገለሉኝም አልረቁብኝምም .እንደገና እንዳልርቅበቸው እነሱ እየቀረቡኝ መጡ

ለምን እነዚህ ሰዎችን መረጥሽያቸው፡ እንደ እኔ እንደይሆኑ ስለፈለግሁ እና ጥንቃቄ እንዲያደርጉ ስለምፈልግ

ከአወቁም ከማወቀቸው በፊትም የሚያሰዩኝ በህርይ አንድ ነው /ለውጥ የለውም/ ፤ጎደኛየም ጋር ይበልጥ ተቀራረብን

ኤችአይቪ ቨይረስ ያደረሰብሽ ተፅዕኖ አለ፡ በበህርይ ለይ

አዎ ፡ እነጨነጫለሁ፡ ለምን፡ እኔ እንጃ የሆነ ነገር ካሉኝ በማያስቀይም ሁሉ እቀየማለሁ ፤እቆጣለሁ ማለት ቨይረሱ ስለለበኝ ከሰው በታች ነኝ ብዬ ስለማስብ እና በቨይረሱ ያስያዙኝ እነሱ ስለሆኑም ጭምር …

በትምህርት ለይ፤ አንድያውን ትምህርት ሁሉ አቋርቼ ነበርኩ፡፡ ለምንድነው፤ ፈርቼ ምክኒያቱም ሊያስቅቁ ይችላሉ ብዬ

አሁን ግን ይህ የለም

**እጮኛ አለሽ** ?፤የለም

ለወደፊትስ ምን ታስቢየለሽ

አለስብም ፡ ለምን እኔ እንጃ ፡ ተጠቃሚ ስለሆንኩ

ለወደፊት ባል ማግባት ብትፈልጊ ምን አይነት ባል ነው ምርጫሽ፡ እንደ እኔ አይነቱን

ከቨይረሱ ነፃ የሆነ ልጅ እንዲኖረኝ እፈልገለሁ

ስለመድሃኒት አውሰሰድ በተመለከተ፡ በፊት መድሃኒት አወሰሰዴ ደስ ሲለኝ እወስደለሁ ደስ ካለለኝ እተወዋለሁ አሁን ለይ ግን አስተካክያለሁ ፡ አሁን 2015 ዓ.ም ጀምሬ

ለምን ፡ የሆነ ሰው/ቤተሰብ/ ከተቆጠኝ አልወስድም …እኔ ሰቆጡኝ አለማውሰዴን ጥሩ ነው ብዬ ነበር ግን አደለም …

የመድሃኒት ብዛት፡ በፊት ለይ ብዙ ስለሚሰጡኝ ይተርፋል ይሰለቸኛልም …በዚህም የተነሳ አልወስድም ..አሁን ለይ ግን ሁለት ፍሬ ስለሆነ አይሰለቸኝም

**መድሃኒትን ከሰዎች ፊት ያለ ፍርሃት ትወስጂያለሽ?**

አይ አልወስድም ፡ ከእኛ ቤት እነግዳ ከመጣ አልወስድም እተወዋለሁ

ለምንድነው ምትፈሪ፡ እንዲያዩኝ አልፈልግም

ወደ ክፍልሽ ገብተሽ ወይም ለሽንት ወጣ ብለሽ ለምን አትወስጂም ፡ አይይ አልወስድም በተሰብም ምወስድ ነው ሚመስለቸው ..አያዩኝም ….

ሰዎች ወደ እኛ ቤት ድጋሜ ለይመጡ ይችላሉ ብዬ ስለማስብ ነው

**የጎንዮሽጉዳትስ፡** የለም

ግን መድሃኒት ሰቋርጥ ራሴን ያመኛል እና ያነጫንጨኛል

የመድሃኒት ሰኣት እንደያልፍበሽስ ምን አይነት ዘዴ ነው ምትጠቀሚ፡

በአንድ ሰአት ቃና ስለለ ቃና ሲጀምር አንድ ሰአት ለይ እወስደዋለሁ አሊያ መብራት ከሌላ ደሞ በስልክ ሰአት ወይም የግድግዳ ሰአት እጠቀማለሁ

**ተሳታፊ 12፡**

ከኤችአይቪ ጋር መኖር ማለት ለአንተ ምን ማለት እንደሆነ አብራረልኝ

ያው መደሃኒት መዋጥ ነው እንጂ እንደማንኛውም ሰው መኖር ይቻላል ብዬ ነው ማስበው

ስለ ኤችአይቪ ሁኔታ ስታስብ ምን ይሰመሃሰል፡ ከአካበቢው ከሆነ ከአወቁ የተለየ ያስባሉ ይህን ያህል ብዙ አይሰመኝም ግን ከእነሱ የተለየሁ እንደሆንኩ ነው ሚሰመኝ

በምንድነው የተለየ የሆንከው ፡ መድሃኒት በመዋጥ ብቻ

ያጋጠሙ ፈተናዎች፡ የለም ጫናስ ፡የለም

ከቨይረሱ ጋር እንደምትኖር ራስህን እንዴት አወቅህ፡ ከልጅነቴ ነው ያያዘኝ አድጌ 12 አመቴ ለይ ነው ያወቅሁ እናት አባት የለኝም የነገሩኝም አሳደጊዎቼ ናቸው ምኖረውም ከእናቴ እህት ባል ጋር ነው

ሲነግሩህ ምን አይነት ስሜት ተሰማህ፡ ምንም አልተሰመኝም

ራስህን ለሌሎች አሳውቀሃል ፡ የለም አለሰወቁም ለምን እንደያገልሉኝ ፈርቼ

ያገለሉህ አሉ፡ እንደ ሰው ይለያያል ማያገልም ሚያገልልም አለ

ከዚህ በፊት እኔን በደምብ ሚወቁኝ አያገሉኝም ግን እንደ ሰው አመለከከት ይለያያል

በትምህርት በህርይ ለይ የደረሰብህ ተፅዕኖ/ጫና፡ የለም

ሴት ጎደኛ አለህ፡ የለም

ለወደፊቱስ እቅድ አለህ ፡ እንደ እግዚአብሔር ፈቃድ ነው እሱ ካለ ይሆናል

ያንተ ሀሳብ ምን አይነት ሚስት ማግባት ነው ምትፈልግ፡ ቨይራሱ ያለበት ሁነ ለኔ ምታስብ ጥሩ አመለከከት ያለት

**ስለመድሃኒት አወሳሰድን በተመለከተ:** መድሃኒት በሰዓቱ ነው ምወስድ አቋርጬ አለውቅም

**የክኒን ብዛት፡** አያስቸግረኝም በአንድ ቀን ሁለት ፍሬ ማታ ማታ አንዴ ነው ምወስድ

**የጎንዮሽ ጉዳትስ፡** የለም

**መድሃኒትን ከሰዎች ፊት የመውሰድ ልምድስ እንዴት ነው ፡** ያለፍርሃት እወስደለሁ

ቢጠይቁህስ ፡ እነግራቸዋለሁ

**የተቋሙ ርቀትስ ፡** ትንሽ ለትራንስፖርት ወጪ ያሰቸግራኛል ፡ አሰደጊውን ነው ማስቸግረው እሱ ነው ሚሰጠኝ

**ከአድሎና መግለል አንፃር ከማህበረሰቡ /ትምህርት ቤት ጎደኞችህ ደርሶብሃል፡**

ስለላወቁ የለም ግን ሚያደርሱብኝ አይመስለኝም

**በተቋሙ ሚሰጠው አገልግሎትን በተመለከተ ክፍተት አለ፡**

ክፍተት የለም በጣም ያሰተነግዱናል

**ድጋፍን በተመለከተ፡** ሚመለከተው አካል ተጠይቆ የምግብ እና የንፅህና መጠበቂያ ቁሳቁሶች ድጋፍ ቢደረግልን ጥሩ ነው ፤ያለው ሚችሉትን ድጋፍ ቢያደርጉ..

**ሌላስ፡** የለም

ቨይረሱ ይህን ያህል ተፅዕኖ የለም መልካም ተፅዕኖ ነው ያለው

ቤተሰብም ያውቃል ፡ ምንም አይነት አድሎና መግለል የለም

ቨይረሱ ከእኔ ወደ እነሱ እንደይተላለፍ ጥንቃቄ አደርገለሁ፡፡

**ተሳታፊ 13**

**ኤችአይቪ ጋር መኖር ማለት ምን ማለት እንደሆነ አብራረልኝ**

ከኤች አይቪ ጋር መኖር ለኔ አሁን ለይ ተቋረኝቼበታለሁ አሁን ለይ ራሴን እያወቅሁ ስለሆነ እንደማንኛውም ሰው ነው ምንም የተለየ ነገር የለውም፡፡ ከቨይረስ ነፃ ከሆነ ሰው ጋር አንድ አይነት ህይወት ተመሳሰይ ህይወት ነው ያለው፡፡ መጀመሪያ አካባቢ ሲነገረን ግን ያለው ስሜት ግን ትንሽ ሚያስደነግት ነገር ነበረው ..እኔ ከሌላ ሰው የተለየሁ ነኝ ብዬ አስብ ነበር፡፡ አሁን ለይ ግን የተሻለ ነው

ስለ ኤች አይቪ ሁኔታ ስታስብ ምን ይሰመሃል;

ለመጀመሪያ ጊዜ ሲነገረኝ በጣም መደነገት ነበር ተስፋ የመቁረጥ ነገር ነበር ኤችአይቪ ሲባል ማህበረሰቡ ለይ መገለል መድሎ ስነየው በጣም አስፈሪ ነበር ራስህ ተስፋ መቁረጥ ነገር ነበር አሁን ለይ በለው የዶክተሮች ክትትል በምነገኘቸው ስልጠና እንደ ሌሎች እኩል እንደሆነ ሁሉን ማድረግ እንደምንችል ነው ሚሰማኝ

በቨይረሱ ምክኒያት የደረሱ ፈተና/ጫናዎች አሉ በዚህ አንድ ገጠመኝ አለኝ

አዎ እንደ አጋጣሚ ነገር ስፖርት/ሰርከስ/ እሰራለሁ በዚህ ሁኔታ የገጠመኝ ነገር አለ ይኸውም ወደ ውጭ የመሄድ እድል አግኝቼ ምርመራ በማደርግበት ሰዓት ኤች አይቪ ስላለብኝ ሳልሄድ ቀርቼለሁ በዚህ ሰዓት ስሜት ወስጥ ገብቼ ተስፋ ቆርቼ ነበር …በዚህ ምክኒያት ወደ እንግሊዝ የመሄድ እድል ገጥሞኝ ቀርቼለሁ …ይህ ሁኔታ ለኔ በጣም መጥፎ ነበር መድሃኒትም ስፖርትም እስከመቋረጥ ደርሼ ነበር፡፡ ከዚህ የተለየ በቨይረሱ የደረሰብኝ ነገር የለም ጎደኞቼም ስላሉኝ ስለመድሃኒትም ሆነ ሌላውን ነገር እነሱን አወያያለሁ፡፡

**ከኤች አይቪ በደም ውስጥ መኖሩን እንዴት አወቅ**

ቨይረሱ እንደለብኝ ያወቁት አስር አመት እያለሁ ነው መጀመሪያ የተነገረኝ በዶክተሮች ነው ከዛ አባቴም ነገረኝ …በሚነገረኝ ሰዓት አባቴም በቦታው ነበር

ሲነገርህ የነበረው ሁኔታ ምን ይመስላል

በጣም አስደንጋጭ ነበር እንደ …ስፖርት ቤት ካሉ ሰዎች ጋር አባቴ ይግባበቸው ነበር እና አባቴ ሲነግረኝ መድሃኒት ምትወስደው ለስፖርት ጥንካሬ እንዲሰጥህ ነው ይለኛል፡፡ ለአቅም ማበረታቻ ነው ምትወስደው ይለኛል፡፡ ስፖርት ያስጀመረኝም አባቴ ነው በሽታው እንደይጸነብኝ እና መድሃኒት ውሰድ ስፖርት ለይ/ሰርከስ/ለይ ጎበዝ እንድትሆን ይለኛል፡፡ ልክ ይህ ተቀይሮ የኤችአይቪ ቨይረስ መድሃኒት ነው ምትወስደው ስባል በጣም ደንግቼ በር፡፡ ከሰማሁ በኋለ መድሃኒትን አቋረጥሁ፤ አባቴ እንድወስደው ቢነግረኝም ልሰማው አልቻልኩም ፤ በዚህ ሰዓትም ሰርከሱ ለይ በጣም ትክክል/በአሪፍ/ ሁኔታ ለይ ነበርኩበት..በዚህ ምክኒያት ሀገር መቀየርም አስቤም ነበር ፤በዘው መቅረትንም አስቤ ነበር …በዚህ የተነሳ ለስድስት ወር መድሃኒት አቋርቼው ነበር፡፡

መድሃኒት በምታቋርትበት ሰዓት ምን ተሰማህ;

የመዞር ስሜት፤ ትምህርት በአግባቡ አልማርም ፤ ስፖርት ስሳራ እንደ ሌሎች ጎደኞቼ እኩል መስራት አልችልም ፤ ሚዛን መጠበቅ የሚሹ ስራዎችን ስሰራ ያዞረኛል ወዲያውኑ ቁጭ ማለት አለብኝ በቶሎ ፤እንቅልፍ ማጣት ፤ጭንቀት፤ላብ ላብ ማለት ይስተወል ነበር፤ በዛ ሽፍታ በአጠቃለይ አቅም ማጣት አገጥሞኝ ነበር ….ይህ ሁሉ የተከሰጠው መድሃኒቱን በአቋረጥሁት በሶስት ሳምንት ውስጥ ነው፡፡

ይህ ነገር እንዴት ተስተካከለ

ከዘበኋለ ፤ራስህን እንትን ምትለው ነገር አለ አይደል

ራሴን ማዳመጥ ጀመርኩ እና እዚህ ማስፅፋው ወረቀት ነበረኝ፡፡ እዚህ መጥቼ ነርሷን ስጠይቃት አባትህ እንዲ እንዲህ…ብሎ ነገረኝ ለምን እንደዚህ ታዳርገለህ አለችኝ ብለ መከረችኝ

ልክ በዚህ ሰዓት ማህበር ሊጀምሩ ሌሎች ልጆች ነበሩና ለምን ከእነዚህ ልጆች ጋር አብረህ አትጀምርም አሉኝ እኔ ሐሙስ ቀን መትቼ ማህበሩ ቅዳሜ ነው የጀመረው፡፡ ሲጀምርም የዛሬ ስድስት አመት በሳበት አበለት ነው፡፡ አንተ ጎበዝ ነህ መምራት ትችላለህ ድፍረት አለህ/ብለው መክረው የማህበሩ አባል እንድሆን መከሩኝ፡፡ ቅዳሜ ስመጣ ሌሎችን ሳያቸው ተፅናነሁ እኔ ብቻ እንደልሆንኩ አወቁ፡፡

ራስህን ለሌሎች አሰውቀሃል

ለሁለት የቅርብ ጎደኞቼ ነግሬቸዋለሁ ፤ ለሌላው ግን አልተነገርኩም

ከነገረሃቸው በኋለ ያሳዩት ምላሽ ምን ነበር

መጀመሪያ ለይ ተደነግጠው ነበር …ከዛ ከቤተሰብ ጋ ነው ስወለድ ነው የተያዝኩት ስላቸው ይህ እኮ ያንተ ጥፋት አደለም በማለት እንደማፅናነት አረጉኝ…እንዲሁም የተሻለ ቀረቤታ አለን..አልረቁብኝም ፡፡

እነዚህን እንዴት መረጥከቸው ፤አዎ ከአስተዳደጋችን

አብሮ አደጎቼ ስለሆኑ አብረን ነው እንትን ያልነው ትምህርት ቤትም እያንዳንዱ ነገር በምንሄድበትም ሰፈር አብረን ነው፡፡ አድሎም መግለልም አለደረሱብኝም

ለሌሎችስ ለምንድነው ያልነገረሃቸው

ለሌሎች ያልተነገርሁት አለ አይደል ራሴን እንትን ለማለት ፡ የሰው አመለካከት ስታይ የሰው አመለካከት ለምንም ማይሆን ነገር ነው ፡፡ ከዘውጭ አንድ አንድ ጊዜ ልንገረው ብዬ ስሞክረው ለአንድ ሰው ቀጥታ አትነግረውም በተለያየ ነገር ነው ምትሞክረው ፤የሆነ ሰው እንደዚህ ተሞ ምናምን እያልኩ ከመነገሬ በፊት በእነደዚህ አይነት መሞከር የተሸለ ብዬ ነው ማምነው እንደዚህ አይነት ነገሮች ለምሳሌ፡የሆነ ሰው እንደዚህ ተሞ እንደዚህ ሆነ ስትለው አመለካከቱን መገመት ትችላለህ፡፡ እሱን ሲጀመር እንደዚህ መቅረብም አያስፈልግም፤ይህ ማለት እኮ እንዲህ እነዲህ ነው ካለ መናገር አያስፈልግም ያ ማለት አንተ ብትነግረው ከዛ የባሰ ነገር ነው ሚሆነው

ራሴን ከአሰወቁ በኋለ ምንም አይነት መድሎና ማግለል አልደረሰብኝም ፡፡

አንተ ሳትነግራቸው ከሰው ሰምተው ያደረሱብህ አድሎ/ማግለል አለ

አዎ እንደዚህ ስትል አንድ ነገር ትዝ አለኝ ትምህርት ቤት ለይ ነው ሃይል ስኩል አስረኛ ክፍል እያለሁ በየትምህርት ቤቶች ደም ልገሳ አለ እና በዛ ደም ልገሳ ወቅት የክላስ ተማሪዎች እንደአጋጣሚ ሁሉም ይሆናሉ ደም የመለገስ ፍላጎታቸው ከፍ ያለ ነው እና ሁሉም ጎደኞችም እንለግስ ብለው ሁሉም ተነስተው የመሄድ ነገር ጀመሩ ያ ማለት ደሞ እኔ እንትን ነበርኩ እና የመጨረሻ ጫና ተደርጎ ነበርና ከእነዛ ውስጥ ደም የሆነ ልጅ የሰፈር ልጅ አለ እና እንዴት እንደአወቀ አለውቅም እሱ ያውቃል ለሌሎች ተነግሮ እሱ የተነገረ እኔን ለመጥቀም ነው አትገፋፉት ተዉት እሱ መለገስ አይችልም ሲል ለምን ሲሉት እንደዚህ ኤችአይቪ በደም ውስጥ አለበት ብሎ ነግሮቸዋል ሲነግረቸው በዘው የተነገረቸው ተማሪዎች አልፎ አልፎ ነበር እሱ ጎደኞቼ ለሚለቸው ነው በዛ ሰዓት ለይ የሆነ አለ አይደል የትምህርተ ሁኔታ በአግባቡ አልሄደልኝም ለኔ …የሰሙ ልጆች ለኔ የሚያሰዩት ቀረቤታና ፊት ትንሽ የተለየ ነበር እኔ ግን በዛ ሰዓት ለይ ቀድሜ ውይይት ለይ ትምህርት ስለወሰድኩኝ ትንሽ የተሸልኩ ነበርኩ ትምህርቴ ራሱ ዘጠነኛ ክፍል ለይ የነበረ የደረጀ ውጤት አስራ ውስጥ ማግባት ምናምን ነበረ ግን ከዚህ በኋለ ነው ሁለተኛ የወጣሁኝ እኔን አጠነከሩኝ እንጂ ምንም አይነት ለውጥ አልነበረም

ከማህበረሰቡስ፡ ከማህበረሰብ እኔ ቤት ብዙም አልውልም ክላስ ነው ምውለው ላይብራሪ ነው ምውል ጌም ዞኖች ለይ ነው ማዘወትረው እና ስፖርት ቤት ነኝ በቃ፤ረጅም ሰዓት ሰፖርት ለይ ነኝ ስምንት ሰዓት ከገበሁ እስከ አስራ ሁለት ሰዓት አልወጣም ጠዋት ከገበሁ እሰከ ዘጠኝ ሰዓት እቆያለሁ ብዙ ጊዜ ለምሳ ራሱ ጊዜ የለኝም ሰፈር ለይም ብዙ አልታይም

ከቤተሰብስ፡ የኔ አንድ ታላቅና ታናሽ አሉኝ እነሱ ነፃ ናቸው እናቴና አባቴ አለበቸው

ትልቅ ወንድሜ ያስተውሳኛል ትንሹም እየሮጠ ለመምጣት ይሞክራል የሆነ ደስ ይላል ሚያስከፋ ቤተሰብ አይደለም ድጋፍ ይደረግልኛል ፡፡ ደሞ አባቴ አንዳንዴ ይረሳል ቀድሞ ይተኛል

**ቨይረሱ በትምህርት ለይ ወይም በበህርይ ለይ ያደረሰብህ ተፅዕኖ አለህ**

ከአወቁሁ በኋለ ትመህርት የመቋራረጥ ፡ መድሃኒት የመቋራረጥ/ጨርሶ አለመውሰድ በህርይ ነበረ እንዳአወቁሁ አከባቢ ከዘበኋለ ራሴን ከአራጋገሁ በኋለ ምንም የለብኝም

በአሁኑ ሰዓት የሴት ጎደኛ /ፍቅረኛ አለችህ፡ እስካሁን የለኝም

ለወደፊትስ ምን ታስባለህ ፡ መቼስ የሰው ልጅ ሁነህ ያን ነገር ካለሰብክህ በህይወትህ እስከለድረስ ያን ነገር አስባለሁ ግን ሚያግደኝ ነገር አለ ብዬ አሁን ለይ አለስብም በፊት አስብ ነበር ማውቀው ሰው እንኳ የለም ደሞ ሲነገረን ከማንኛውም ሰው ጋር ግንኙነት ማድረግ እንደሌለብን ነው በደም ንክኪ በስለታማ ነገር እንደሚተላለፍ ስለሚነገረን በዚህ ምክኒያት ሰዎች ማግለል ብቻ ሳይሆን ራስህን ከሰዎች ታገለለህይህ ነገር ነበር በፊት ለይ ከዘግን እዚህ ከጀመርን በኋለ ሰዎች እንዳሉ ስታውቅ በቃ ሚገድብ ነገር እንደሌላ አወቅሁ ያ ማለት የሴት ጎደኛ መያዝ ትችላለህ አግብተህ ትዳር መያዝ እንደምችል ነው ሚሰማኝ

የእጮኛህ የቨይረሱ ሁኔታዋስ ፡ እንደ እኔ ተመሳሳይ መሆን አለበት ብዬ ነው ማስብ

ስለኤች አይቪ ስርጭትና መከለከያ ዘዴዎችን በተመለከተ ምን ታስባለህ፡

ከስርጭቱ አሁን ለይ በለበት ሁኔታ አሁን ለይ የተሸለ ነው ብዬ ነው ማስበው የተሸለ ነው ሰውም ትንሽ እውቅና አለው ፡ ትንሽ ባሰበት ነገር ምትለው እኛም ትንሽ እያየን ነበር የሆነ ሰሞን ለይ በዚህ ጉደይ ሶሻል ወርከር ለይ ስራዎችን እየሰራን ነበርና እእእ..ቀበሌ እትን ልበልህ ሴተኛ አዳሪዎች ሚበዙበት መጠት ቤቶች ሚበዙበት ግሮሰሪ ሚበዙበት አከባቢ ለይ ትንሽ በተለየ ሁኔታ ስርጭቱ ሚስፋፋበት መንገድ አስተውለናል፡፡ ያ እንዴት ነው የሆነው ሰው በተለይ መጠት ቤቶች ለይ ማሰብ በአልኮል ለይ የተመረኮዘ ስለሆነ ኤች አይቪ ብዙውን ጊዜ ሚተለለፈው ደሞ ጥንቃቄ በጎደለው ግብረ ስጋ ግንኙነት ነው እየተበዛ ያለው ይመስለኛል ከዘበለፈ ሰው እውቅና አለው ሁሉም የተማረ ማለት ትችላለህ በከተማ ህብረተሰቡ ለይ አሁንም ድረስ የተወቀ ነገር የለም ከመግለልና አድሎ ለይ ቢታይ ከድሮ ይሻላል በዚህ ዙሪያ ድጋፍ ሚያደርግ ድርጅትም ስላለ ከበፊቱ ይሻላል

ስለመድሃኒት አወሳሰድ በተመለከተ እንዴት እየወስድህ እንደሆነ ተነገር፡

እኔ መድሃኒት በመጀመሪያ እኔ ቅዳሜ ቀን እየመጣሁ ነው ከጤና ተቋም መድሃኒት ምወስደው ከወስድኩ በኋለ በየቀኑ አላርም አለኝ ሁሌም ሁለት ሰዓት ከአምሳ ሰባት ደቂቃ (2፡57) ለይ ይጮሃል ከዘበኋለ ሦስት ሰዓት ለይ እወስደለሁ፡፡ ሰዓት አለሻረርፍም በሰዓቱ ነው ምወስደው፤ ምክኒያቱም ለመኖር ጠንካራ የሆነ እምነት ስላለኝ

ረስቼው ብተኛ እንኳ ወንድሜ አለ ከዘ ከላፋ ሊረሳይችላል

የክኒን ብዛት በተመለከተስ ፡ አንድ ፍሬ ነው ምወስደው ምንም አያሰለቸኝም

መድሃኒትን ከሰዎች ፊት ከመውሰድ አኳያስ ፡ብዙ ጊዜ እኔ ኮንዲሽን/ፊልድ/ እገባለሁ መለትም ለስፖርት ውድድር/ሾው ለማሰየት ስሄድ ይጄ ነው ምሄደው ወደ ሌላ ሀገር ስንሄድ አንድ ክፍል ነው ምንይዘው በዛ ሰዓት ለይ ሰው ይበዛል …አንዳንዴ የሆነ እንትን ማለት ነው ያለብህ የሰዎችን እንትን/ትኩረት/ መቀየር ዛሬ ጉንፋን ይዞኛል ብዬ እወስዳለሁ ብዙ ሚያጠራ ሰው የለም እንደዚህ አይነት ነገሮችን መጠቀም ዛሬ ራሴን አሞኛል ፓራስታሞል ልወስድ ነው ብርድ ብርድ ብሎኛል ነገር በማለት የተለያዩ ነገሮችን በማሰበብ እጠቀማለሁ፡፡ ከዘውጭ የኤችአይቪ መድሃኒት ነው ብዬ አልነገርም

የጎንዮሽ ጉዳትን በተመለከተስ፡ ከልወሰድኩት አለ

መድሃኒት እየወሰድኩ ምንም አይነት የጎንዮሽ ጉዳት የለም ከአቋረጥሁት ግን የራስ ምታት ስሜት እንቅልፍ ማጣት አንድ ቀን ሳልወስደው ብቀር በነጋታው ድብርት ይይዘኛል፡፡

በተቋም በኩል ያለው የአገልግሎት አሳጣት/እርካታን በተመለከተ

መጥቀስ ከላብኝ ምናልበት ከጤና ተቋም ጋር ምንመጠው ቅዳሜ ቀን ነው ያም ትምህርት አይኖረንም ስራም ብዙም አይኖርም በመከተል እዚህ ከመጣን በኋለ የትራንስፖርት አለን ትንሽ ቢሆንም አምጦ ይመልሰናል በቂ ነው የታክሲ ይሸፍናል አያሰቸግርም

ሌላስ ማን ምን አይነት ድጋፍ ማድረግ አለበት ትላለህ

እኔ ማን ለሚለው ይህ ብዬ መጥቀስ አልችልም ግን ሁሉም ሰው ግዴታ አለበት ብዬ አስባለሁ እያንዳንዱ ድርጅት እያንዳንዱ ተቋም በዚህ ዙሪያ ሚሰራ በሙሉ ድጋፍ/ትኩረት ማድረግ አለባቸው ብዬ ነው ማስብ ለምንድነው ምለው ሁሌም መድሃኒት እየተወሰደ ነው ሚኖረው በዚህ ሁኔታ ውስጥ እኛ ብዙ ጊዜ ስብሰበዎችን ከዶክተሮች ምክሮችን እናገኛለን ግን እንደ እኛ ብዙ እውቀት ያለገኙ አሉ እነዛ እስከ መሞት የአልጋ ቁረኛ እስከ መሆንና እስከ ሞት ደረጀ የደረሱ አሉ ይህ ነገር ለምን ሆነ ለሚለው ደሞ የድርጅቶች አለመቅረብ ነው /ብዙም ቀርበው አለመስራት / እነዚህ ልጆች ድጋፎችን ማግኘት አለባቸው የተለያዩ ሁኔታዎች ቢመቻቹላቸው በዶክተሮች ሚያገኙት ህክምና ብቻ በቂ አይደለም ፤ መድሃኒት መውሰድ ብቻ በቂ ነው ብዬ አለምንም የተለያዩ ስልጠናዎችን ማግኘት አለባቸው የህይወት ክህሎት ስለጠናዎችም ጭምር ማግኘት አለባቸው ከመድሃኒት ጋር ያለውን ነገር እንዴት ማስተዋቅ እንዳለበቸው ራሱ መድሎና መገለል ከመድሃኒት ቁርኝት ጋር ያለውን ነገር በሰፊው ቢያገኙ ያንን ነገር ማድረግ ቢችሉ የተሻለ ነው ብዬ አምናለሁ

በባለሙያዎች በኩል ሚታይ ክፍተት አለ // ከተቋሙ ማስተካከል አለበት ምትለው ካለ

ቢያስተካክሉ ምለው ሁለት ነግሮችን ነው

አንዳኛ ልጆች ከመጡ በኋለ እዚህ የሚሰጠቸው የምክር አገልግሎት መስጠት ብቻ ሳይሆን በየወሩ ክትትል ቢደረግ …ዛሬ/በዚህ ወር ወስጥ ምን ያህል መድሃኒት ሳትወስድ ቀረህ ተብሎ መጠየቅ ክትትል መደረግ አለበት…በተለያዩ ምክኒያት መድሃኒት ሳልወስድ እቀረለሁ….ለምሳሌ ሰርግ ሂዤ፤ ግሮግራም ኑሮኝ ፤ ፊልም እያየሁ ረስቼው ሊል ይችላልና ለዚህም ግሮግራም /መፍትሄ ቢነገረው …መድሀኒት እንዲወስዱ ተፅፎለት ብቻ መሄድ የለባትም …ሁኔታዎችን ሰዓቱን እንዲያመቻች ምክር ቢሰጠው አሪፍ ነው ብዬ አስባለሁ ..እንደዚህ አይነት አገልግሎት ከመስጠት አኳያ ትንሽ ክፍተት አለ

ሁለተኛው ደሞ ቅድም እንዳልኩህ እዚህ ከድርጅት ሚመጡ ድጋፎች ናቸው እነሱ የሆነ አንድ አንድ ቦታሂደህ ስታይ ጤና ቢሮም ካናል ፕላስ ከኤኤም ኤስዲ ከማህበራዊ ሕይወት ከተለያዩ እንደዚህ ድጋፍ ተደርጎል ልቀናል የሚል መልስ ይሰጡሃል ሁለችንም ምናውቀው ነገር ስላለ ማለት ነው ከድርጅቶች ይህ እኮ አልፎል ..ይባላል ልክ ዶክተሮች ጋር መጣን ስንጠይቅ ሚሰጥበት መልስ አልደረሰንም የሚል ነው መሃል ለይ የሆነ ችግር ያለ ይመስለኛል ከድርጅቶች ተልኮል የተባለ ነግር እዚህ አይደርስም …ይህም የዶክተሮች ችግር ነው ብዬ ነው ምወስደው

ሌላው ልጆች ጋ በደምብ መሰራት አለበት በይ ነኝ ምክኒያቱም ቅድም እንዳልኩህ የአልጋ ቁረኛ እየሆነ ያሉ ልጆች አሉ እኛ እያወቅነቸው የሞቱ አሉ እሰከ ቅርብ ድረስ በዚህ ሳምንት በዘኛው ሳምንት ሁሉ በየሳምንቱ አንድ አንድ ልጅ ይሞታል በቅርቡ እኛ ምናውቃት ልጅ ሙታለች ..ይህ ነገር እየሆነ ያለው የሰው ልጅ አዕምሮው ለይ ስትሰራበት ነው ስለመድሃኒት ያለውን ነገር በደምብ ስታስረደው ከገበው ምግብ እንደሚበለው ነው ምግብም ሚበለው ጥቅሙ ስለገበው ነው ለምን አያቆምም ታዲያ መድሃኒት ማይወሰድም ስላልገበው ነው ምንም አይሰረልኝም ስላለ ነው ፡፡ እዚህ ስለመጡ ብቻ መድሃኒት ብቻ ሰጠው መላክ በቂ አይደለም …በቂ የሆነ ስልጠና መውሰድ አለበቸው ሁኔታዎች ተመቻችተውለት ከተለያዩ ሀገረት የልምድ ልውውጥ መውሰድ ለምሳሌ እንደ ደሴ ኮምቦልቻ የተለያዩ ድርጅቶች አሉ ማህበራት አሉ ከእነሱ ልምድ በአገኘ ቁጥር ያ ሰው እኔም እንደ እነሱ ነኝ እኔም ያን ነገር ማድረግ እችላለሁ ብሎ ማመን እንዲችል በደመብ በደምብ ልጆች ጋ መሰራት አለበት ይህ ከሆነ ሚሞቱትን መቀነስ ይቻላል

ይህንን እየሰሩ ቢሆኑም የተለያዩ አካለት ስለመድሃኒት ቁርኘት ዙሪያ በደምብ ቢሰሩ ጥሩ ነው እላለሁ፡፡
