# Supplementary material for: The experience of living with human immunodeficiency virus among adolescents at Felege Hiwot Comprehensive Specialized Hospital Bahir-Dar, Northwest Ethiopia, A phenomenological study
Source: PLoS One. 2025 Jan 9;20(1):e0308347. doi: 10.1371/journal.pone.0308347 (PMC11717265; doi:10.1371/journal.pone.0308347)
Supplement: S2 File — (DOCX) [file pone.0308347.s003.docx]

**Participant One:**

**What does living with HIV mean to you?**

**Answer**: To me, living with HIV means eating and drinking like any other person, but this only happens when we take our medicine properly. Now, for example, there are many people who have HIV without being diagnosed. In the early 1980s, HIV was stigmatized. People were dying without taking medicine, one of their legs was amputated, and they used to have a separate bed.

Takes nutritious food. I also take the medicine properly. I eat food. I will fast. I don't have a problem because I take it at night.

**How do you feel when you realize that you are living with the virus?**

I was diagnosed with the virus when I was three years old, but when I was in the fifth grade I know that I am living with the virus. I don't feel anything, but I don't want this to happen to my brothers and sisters. In this case, it is good if you follow this matter because it is not only for them, but also for them, because they need someone to accompany them. For example, women should be monitored during pregnancy; Men should also go together and develop what is called learning. It should be given a test in any place.

**Do you support that learning/to have a test/?** Yes, I support it.

**Do you have something to look forward to/challenges faced due to presence of HIV/AIDS?**

There is nothing I can call a challenge. But one time for some reason I stopped taking medicine and it hurt. It has a headache, but it has been going on for about 5/6 years now, and it hurts when I stop taking medicine.

**Why did you stop taking medicine?**

I went somewhere and when I left I forgot the medicine, I left for a week and when I came back I felt sick then I stopped taking the medicine.

**Are you affected by the virus/any impact due to HIV/AIDS?**

Nothing has happened to me yet / it will be seen in the future.

**Who supports you?**

My sister, my sister's husband and my father support me.

**Who told you that you have the virus/how did you know?**

When I was a baby, I used to take Plumpy Net and syrup and I hated it so much and when she gave me Plumpy Net, I would say no to my mother. My mother told for me that you take the medicine so you don't die, that's childish stupidity because I didn't feel anything/I didn't feel anything. If this thing were told to me now, I would have felt a terrible feeling/I would have been very hurt. When I grew up, I knew that and was emotionally hurt. Now I think I was the only one who knew.

**In the future, do you think it would be great if they were told at an early age?**

**Yes;** it is better if they are told when they are young, because children are clean sheets of paper and they don't know anything, so if they are told this at the age of 9/10, it is the right age. Because at this age they focus on their play and they don’t think more. At this age they don’t think about the medicine and virus.

**Do you have a change of mood when you were told?**

I didn't have any change of mood.

**Have you announced yourself to someone else (others)**?

Yes, family knows, for example, my aunt/cousin's husband knows, and my closest/best/friend. She said that this does not mean anything and I know because I am a nurse's son.

**How did you choose her:** because she is my friend of 4 years

**What were the circumstances when you told her?**

When I told for her, it would have been much easier for me. I was tired of hiding this from her. It doesn't mean that she advises me like this when she is old, and if you say so, it includes our relationship.

**Why didn't you tell for others** / because there is still stigma and discrimination.

There is a person, who doesn't pretend to have the virus at school, and he doesn't participate in this program (OTZ), but only 5% of the community will be free from the virus, that is, most of the people will be infected with the virus, because people don't practice abstinence properly.

My father is the only one we have, my mother is dead.

My friend is closer than I told you, but she left, she didn't run away, and because she is a nurse's daughter, there is no such thing as isolation from **her/my friend**.

I was not stigmatized because someone else heard it.

**Effect of virus on education**

I don't know how the virus affected me in school, but without the OTZ program, it was difficult for me to attend school, that is, I had to miss school to take medicine, or my sister would bring medicine to me. OTZ program is on Saturday until six o'clock.

The other fast was very good. I like it because the medicine is in the morning and at night. My father doesn't allow me to fast. He wanted me to eat breakfast and take the medicine in front of him. He wanted me to take the medicine and go to school.

**Is there any effect on your character due to virus?** No it’s a natural anger of my character.

**Do you have a boyfriend?** I don't have

**What are your plans for the future?** The creator of the future knows.

But even if there is a negative person, I can't handle it. It's hard. Even if I have it, I'd prefer it to be a similar person. If you say something, he can accept it. If he is negative but doesn't love me, he will leave me, but if he truly loves me, he will stay with me. If he leaves me, this is very difficult for a girl.

**How do you see the spread of HIV/AIDS?**

I don't know whether the spread of HIV will decrease or not, but like us, they wants to give life here, even if others give their life, it would be good if they understand that they are not the only ones and talk about it.

**Who should do what to expand OTZ?**

The government should take responsibility and expand it. If the government releases a budget, the doctors will not say that they will not curse because they know the condition of the child and this is expected from the government.

It would be good if they could come together like us in this program, if you could help, and if you knew someone else

Life is lived while taking medicine, life passes even if you are bedridden.

**Do you want to have a child in the future?**

A child is God's grace, but I don't want my son to become what I am his mother. *I will use medicines to prevent the virus from being transmitted from me to him. I don't want my son to guess/repeat it, but if it happens unexpectedly, I will tell my son when he is a baby.*

**What are the precautions you can take to have a child free of HIV?**

Follow up and consult doctors and use sharp objects separately

**Medicine intake experience;**

I first started with syrup at Health Center, and then it changed to pills. Aunt, my cousin's husband and father make me take it. I don't know if I have time. Even if I have a job, I cut it down to take two hours. I don't hire an hour.

Once I spent an hour at a show and my brother was angry with me. This is for me

**Regarding the number of pills**

At some point I fainted and was sick and I was given a lot of medicine and it was too much for me, but after that it didn't bother me at all.

**Do you afraid to take medicine in front of people?**

**Yes**, if a guest suddenly comes, I will bring water into my room and take it, or I will take it outside. I take/do what I have to do earlier.

**Regarding side effects of medicine**

Anything that works has benefits and side effects. I have not seen any side effects so far. If I don't take the medicine properly, I can become bedridden /sick. It is said that it will not kill, but this disease will kill, so I will take medicine properly

**Who should do what for the adolescents?**

I mean, not only for me, there are children who need support

Support is needed for children, support is not only financial, but also psychologically with love, they should be cared for, everyone should show love.

You can quarrel with family and cut medicine, so it is better for parents to argue in front of children / Family arguments make children take medicine.

Even if the government supports professionals, the reason is that Sunday and Saturday are for us. It is for us that they come in on their day off.

In addition to this, even if everyone is examined and knows himself, medicine will be taken; Life goes on. No, this covid also kills, HIV also kills. Everyone should be tested. Just like covid, HIV should be tested on time. If we don't take medicine, it won't give us time.

**What does the community look like in terms of showing love?**

I have two positions from the community

One Sometimes there is a good society and sometimes there is a bad society. When I say badly, it means they may not showing a good face/ showing love.

The other way, I mean showing love. The society should see us as their children. When the creator first created us, each one of us was born with love. The creator gave us all love. So now we have given away something called money. Everyone is making money and making money out of love. There are many people who are taking medicine and are isolated from the society. Even if the society shows love, the reason is that the virus does not spread peacefully. It comes from our own selfishness. Others can interfere by applying the laws of God and everyone should know themselves and give love to others

**In terms of showing love from health professionals**

The masses give love/discuss with love in professions; Treat them. They care but rarely Now, there is a profession that produces a spark/occasionally there are professions that produce a spring. It is a unique thing.

**Any additional thing**

Finally, what I want to say is that my father gave me hope, which is that I will not take medicine, so don't worry, take it for a year, and once you are injected, I will buy you a medicine that will be given by injection for a year. If this medicine comes, it will be very cool for the health and for us, because it took me hours to take it every day. Don't think I forgot. But now that I think about it, I understand that my father knows and is giving me hope. It would be good if this medicine comes to us for one year/six months.

**Participant two**

**How would you describe living with HIV/AIDS?**

Yes, HIV is not what we wanted. It comes from our families, but it is necessary to accept what has come, why do you need to do unnecessary things because it is **not** my fault, you need to accept what has come and follow it by communicating with doctors and when you are diagnosed with HIV, by taking medicine properly, you can live like any other person and you can reach a good level of isolation, there is nothing difficult. You can reach the level, there is nothing difficult

**How do you feel when you think about HIV/AIDS?**

Sometimes I can't come out and say it to my friends. There will be something that will have an effect. Now, for example, if you take a class and say that I am like this, something very difficult could happen to me. Of course, I think something because of the virus. It doesn't mean that you won't think about it. It is possible to follow someone like me if I don't change. I am worried that the response from the society may be a good thing. I am worried because of the fear of isolation.

**Are there any challenges you have faced due to virus?** There are no challenges

**How did you know yourself/who told for you?**

I started taking medicine since I was a child. I grew up in the same place. My family didn't tell me that you are like this. I found out through my own process. When the association was established, there were 8/16 members of the groups. I was one of them, so the association made us aware. When they told us, I didn't think anything because I was taking medicine.

**What was the situation when you heard?**

Since many of my friends were with me, they used to tell me in different ways, for example, that the medicine is to be taken for life long.

**It was a great feeling if you knew you had the virus**

The family knew, so they followed me. I didn't feel anything, but I was **afraid.**

**Do you have made yourself known to** **others**? No, they do not know other than the family

I don't want to talk to them because now the society is said to be educated, but in practice it remains. The interviews coming from them are not good.

**Effect due to the virus**

There is no pressure caused by the virus, it is an effort

**Do you have a boyfriend / lover?** No

I don't think I'll have a chance. If my life is settled and settled like I am now / if I can manage and support my family on my own / not only because I have the virus, but even if I have it, I want to just follow it.

I don't have that much desire to live with my family; I want to live with my mother and sisters

I say that I don't need a female friend for the future, I don't need to separate now, but I am a person for the future and it won't be forgotten.

**What is your choice of girlfriends HIV status?**

If she accepts me, I don't think there is any problem if she accepts me. If she accepts me, we use condoms during intercourse.

**About taking medicine**

Today it is taken once a day and once at night, but in the past I used to take it three times a day. I have to take medicine regularly to get there

**Do you take the medicine freely in front of people?**

No; I take medicine at night, sometimes uncomfortable situations may occur.

**Is there any side effects? No**

**Are you tired of taking medicine all the time?** I don't know

**Regarding isolation**

I have not heard it said that it should be like this, but it is not known

Another thing is that I have seen that there are many types of people, I mean, there are all those who have died from taking medicine, and better medicine is coming every year. I hope that there will be a cure in the future

**How do you see the isolation of care given by professionals in terms of service delivery?**

**No problem from professionals, they treat us well.** Nothing else to say, I expect good things from the study.

**Participant three**

**How would you describe living with HIV?**

Living with HIV means no one likes disease for me, but even if the disease comes, I am equal to a person, I am not inferior to a person, and I am not discriminated against and stigmatized by HIV.

**Do you have the feeling due to the virus? No**

**What do you think about the virus?**

I think I'm alive and I'm happy with that, I don't feel any different.

**Do you have a problem/challenge?** Yes, if the disease gets a small gap, there is a possibility of getting stronger, but I had an operation due to appendicitis. It was a bit difficult on time. For me, the operation was too late. It took up to four hours. There was pus. Because of that, the drugs are heavy. It is not easy for many people. I used to take medicine and I consider this as the biggest challenge. There is no other problem

**How did you feel about the operation?** During the hour, I didn't feel anything. I was just sick and tired. They knew that if I went out, a person made like me would not have taken so much medicine. Due to this I think more.

**What were you thinking**? **I was thinking about the amount of medicine I took**

**Do you have announced yourself that you have a virus?**

Only family knows but not others

**When did you find out/know yourself?**

I started taking medicine when I was a child and it was given to me through breast milk. I didn't know anything at the time and the family was telling me that the medicine was for another disease... When I asked the nurse, she told me that clearly and appropriately, and here, the association has registered me.

**How did you feel when you heard that?** I'm not like other people on time. I had not faced any psychological challenges/feelings.

**Why didn't you tell your friends and the community that you have the virus?**

From the perspective of the community, that is, the friend you share with doesn't say the problem is my problem. I didn't tell them. **If you know yourself, you have a strong feeling**

**Are there different effects of HIV on education**? No

**Do you currently have a** lover/girl friend? I don't have one

**Do you have a plan for the future?**

I prefer the one who is similar to me. Even if it is transmitted from the virus, we should consult each other to prevent this, for example, reduce the frequency of sexual intercourse, follow the advice of doctors, and take the medicine on time.

**What are the precautions you should take to have an HIV-free child?**

The child can be born free. To make this happen, you need to monitor the pregnancy and follow the advice given by the doctor.

**What is your experience of taking medicine?**

I always take it at 3:00 p.m.;

**Who is reminding you?** Yes, there is no need for anyone else; conscience itself tells you to bring food as you remember. In other words, it does not make you hungry, but it remembers you, you go to medicine, you forget about it, you are cursed to cure the disease, or you are careless about yourself, it means that you have no desire, but when you want to hunt, you do a lot of things, for example; Walking every hour, swallowing, at night, when I have program to see football in DSTV house, I take medicine in my pocket and water. When the time comes, I go out and take it back.

**Are there a side effect related to the medicine**?

No, maybe the acne on my face was a pimple when I was doing the surgery. I didn't wash for six months. I heard that this bruise was caused by an operation, and they said that HIV can cause it like this, but I didn't know what the problem was because I passed my medicine, and I didn't judge that it was because of the acne.

**Does the distance make it difficult for you to take medicine?**

No difficulty, there is access of taxy.

**Regarding the cost of taxi**, since I am a student, I receive it from my mother. When they don't have a home, they accept it from their friends. This issue is not difficult, but because I don't work, I take it as a challenge when I call it difficult.

**Regarding the number of drugs**

I used to take a lot, but after 3/4 years, I only take one tab. This is also convenient for taking because it is convenient to take at night.

**Deprived of service, especially the problem/gap seen by the hospital**

Even if we completely run out of medicine, there is no shortage of medicine, but taking for one month what is given for three months

**Is there a visible gap in the provision of professional services?**

There is no gap because we have started them in the past. To tell the truth, if we say that we were raised by professionals rather than our families, it would not be an exaggeration. The care given by professionals is very cool.

**From the community side**

From the side of government; Almost the government is doing well for us and the ID that will be given tomorrow is very cool. If I happen to go to other country, I can get medicine by showing this ID.

**Due to the virus, do you have stress/change in behavior?**

This does not bother me but I need a lot of food because not only medicine is needed to stop the disease but also food. I don't have any other worries but this thing. It's your personal story that makes me worry. The other thing is when children fight with the family the child refuses taking medicine…….

**What is the reason why you did not tell yourself to others?**

I will tell you what I saw on TV, at Hawasa, **a women**, who calls her aunt, as HIV, and then the community accepted her and started calling her HIV, her real name is Yemisirach but society calls her HIV In addition, she was raped and gave birth to an 18-year-old child due to this she had faced different problems, but the government gave her a house...the moral compensation was paid to her. The attitude of our society has not changed much; tomorrow there is nothing to be said about me. / The mentality of our society has not changed much. There is nothing to say about tomorrow/. Notifying this is not a matter/obligation, but it may be a situation where it becomes an obligation. For example, when a car accident occurs, you can tell your friend so that he can be careful, because I have to see my friends as my brother. You can tell him about this matter, but you won't tell him that it's a rumor/you don't call it gossip.

You can tell your friend when he arrives, so that he can be careful, because I have to see your friend as my brother.

**Where do you want to see yourself in the future?**

What should you do/who/what should you do to get to this place?

Learning is especially important. It is not only about education that needs to be financially supported. Those who are good in education can also be selected and get a job. If they graduate, the government should start/create opportunity to work, that is, those who live with the virus should be prioritized. /OTZ program, we only have it separately, for example, there is no in Adis Abeba, not because I went and saw it, but because a boy who came from there told us, they don't have t-shirts in OTZ.

**What is the attitude of the community?**

If you tell the community, when you hate him, he will reveal the secret to you. If you don't tell him, he will believe you and accept you. Even if you tell him, even if you don't tell him about something that you did not tell him, for example, if you tell him, if you try to kiss him, he may say, "Don't touch me when you try to kiss me."

If the other gives a lesson on sexual issues / for example around girls/, the society should have a sign saying that the one who has the virus should be protected with a condom, and the person with the virus should be protected by peeling.

Through sexual intercourse, the society should protect those who have the virus with condoms, and they intention to perform sex without condom/they have a sign saying that it is because of peeling/.

**Who are they who say that it is done with a condom?**

People with the virus: This is wrong. Even if you use a condom, there is a strong possibility of transmission during intercourse.

**How do you take care of your future partner?**

I don't think that I will use a condom because I have a woman who is positive like me

Since I am the first and last child for my family, I will be well taken care of.

**Participant four**

**How would you describe living with HIV?**

Living with HIV, we live like any other person. There is nothing different. The virus is in the blood. It is like a young person living with the virus. When I live in this situation, I am doing the things that I need to do to prevent the virus from harming me. By doing my daily routine carefully so that I have; By taking anti-HIV medicine properly, by monitoring my health, by monitoring the amount of the virus, by knowing my health condition and by consulting with doctors, I will have better health.

**How do you feel when you think about HIV?**

When I was told about my attitude because I was a child before, it is difficult to relate it to the society's attitude at the time. It can affect your confidence again because I am a child. Since I am a child, we think that we can’t play with our friends equally. If they knew me by any chance, I felt that I would not be able to do many things together as independently as they did.

Now for this; now, when I am doing health monitoring here, with the advice given from time to time, there is an association of young people who use anti-HIV drugs or have HIV in their blood. I have been a member since I was a child and I have been a member since I was a child.

The reason is that there are people who come from faraway places, so we will be open for services on Saturdays, and the service will be open for students under the age of 19 through the OTZ program. I have my own group in the congregation, I am the representative of my group and I have enough understanding. In this way, **Sister (health professionals)** will facilitate us.

As the head of the department, I take care of the children under me. For this reason, I am the representative of our champion. We are getting used to this kind of discussion. Before, when I came for follow-up, I was with my mother, but now I come alone and take medicine. I am eligible for the championship by being selected. We will have a peer-to-peer discussion with our friends.

**Are there challenges due to virus?**

There will be psychological pressure/burden/, for example, a stranger may come to the house, even because I don't tell them. There is psychological pressure because of what he did to take medicine hidden from them. When there are strangers, I go into the bedroom with water and finish taking medicine.

The experience I have heard from other children is that some of them may take medicine with saliva when they swallow in these unfavorable conditions, and that medicine may rest in their stomach because it is convenient for them to swallow on their own. They face challenges of getting into addiction due to their bad habits.

Now I don't have this mental pressure/challenge/

**Who told you that you were living with the virus/how did you find out/who told you/where/what were the circumstances?**

I used to follow up here and I did not start with children. I did not take medicine for about eight years. Then, when I was in the 2nd grade, I was about 9/10 years old and children under 15 years of age must take medicine because it is already prescribed. At this time, my mother said that she should not take it, she could only stop it with food.

No, it's a forced condition, so I started taking medicine, so it was difficult for me because I didn't get used to the medicine I had difficulty of sleeping.

**How did you feel when you heard that?**

On the day I was told to take medicine, the day I was told why I should take it, I was immediately asked why, so my mother took me to the bedroom and told me that you must take medicine because you are infected with the virus. I used to take it in the morning and in the evening. She told me that any person can be healthy by taking medicine. She told me that the time of the medicine will pass and I will hire you. She scolded me a lot saying that I would suffer from mental pressure. Also, I used to take it in the morning and at night when I was tired from playing. The medicine is improved and taken once a day. First of all, she said there is pressure, why am I taking medicine and when will the other one take it? Like other people, I live by taking food. When I eat, my mother explained it to me very well.

**Do you have made yourself known to others?**

No, they didn't know. But there may be common ways, for example, when there is a blood donation, it raises the question of why you don't donate?

I will make them live

Finding a solution by wearing it at this time is because I don't gain kilos/weight/, or changing my mind by saying that I don't have the experience.

**Why did you not want to inform others?**

First, there is a problem of attitude in the community, so I don't think that the community is educated. If the society hears it, they can be stigmatizing from social life. They can also make a suggestion and from the point of view, when we are playing football, something can be said to them or to me, things that build together.

Also, from the point of view of being able to make suggestions, they can say something to them or to me when we are playing ball. They can say something to them or to me. There are things that they have built together, for example, there is drinking water in a highland and eating together. I understand that I can't follow along with them and thus the psychological pressure is reached.

**The effect of HIV on your education:** There is not much, thank God

In the past, I may have to eat when I am fasting. When someone is fasting, why don't they fast?

I used to think about this issue, but now I take medicine once at night so I don't think about it, and as I get older, my understanding increases.

**Do you currently have a girl friend**? I don't have one. Of course, there are many kinds of social relations because there is education here. Sexual desires can also occur at this age. But how can I deal with this? I have received a lot of training in the past. **No**

I don't have any sexual relationship with anything other than class/neighborhood friendship

I always think that when I grow up, I will finish school, graduate from university, start my own business and start a family. When I think about that, I ask myself what will happen to me. I have the idea of ​​giving birth to a free child by making a woman who is in a life/health condition a personal part of my life, by using the prevention, by using various things, by using the medicine that has been treated.

**Regarding taking medicine**

When I used to take it, I didn't tell you in detail, it used to be in the morning and at night, now I only take it once a day at night, which depends on the size of the virus and the condition of the disease. My viral load is at the level of ``best/First'' and since I am in that condition, I can only take one tab.

**The experience of taking the drug in front of people without fear**

Although there are people who know about it, I take it because it is not bigger than life

**Are there any side effects?** No

**The distance of health institution in relation to cost**: I used to think about it before. I used to think about how it is to walk every month, but now that we have come to this neighborhood, it doesn't bother me.

But when we talk with my friends, there are children with low income who come from near and far, and they come to Aske, one by one, because they come here because they say that this program will improve our transportation, we see them struggling when it is said that there is no budget.

**Where do you place yourself in the future?**

After finishing my studies at the university, I want to be part of the government consultant in the social sector. I have my own reasons for saying this.

When I consulted the people in my secondary school, they told me that it would be good if you worked around this.

**What is expected from different institutions?**

Different stations provide support in different fields.

Both the government and humanitarian organizations should pay attention and work. There may be people who have many talents who hide themselves in addiction

The government also paid attention to making productive power available

**How is the service provided by the hospital, especially the service provided by the professionals?**

There is no problem on the part of the professionals. I want to thank the hospital on this occasion. They have done a wonderful job by giving psychological advice to people like this.

The main thing is that when you live in this health condition / with the virus / it is psychological pressure and for this I have received counseling services through this program, but there may be children who do not have this opportunity. If a structured job opportunity is created; If our society is made aware, HIV needs to be taken care of just like high blood pressure and DM.

**Participant Five**

**What does living with HIV mean to you and how would you describe it?**

I'm a normal person, I'm living like any other person, I'm living, I'm not missing anything, I'm alive, I'm the best of all, so no one can see me and know that I have HIV-AIDS. It's different from the way he lives, we are living where we get checked up and we get checked up, he doesn't go once a year, but it's not easy for us to get checked up every month. And it doesn't mean anything

**How do you feel about living with HIV?**

I don't feel anything because I only recently found out that when I was a child I was greatly affected by real life, but now it doesn't seem like it's just me and me.

Being depressed doesn't feel like this, yes

**Are you stressed due to the virus/effect due to virus?**

It used to be said that I had a mental problem, because no one knew; only my mother and I knew, and the doctor himself could not know that I had it. I forgot that I quit because I joined this company, I left because I had a strong psychological problem.

Yes, for example, I don't talk to anyone, I come home from school, I walk, I sing, I'm at home, I close my door, I deliver essays one by one.

**What kind of essay;**

For example, from the point of view of the children at the school, one is about the love of the country, one is that we are following the outside world separately; Thoughts come to me one by one. It means that they told me, but they understand me, it means that they read my book and there are many things, but I was not affected by HIV AIDS. People either believe that she is crazy or that she is in love.

**Did you experience this effect before or after you became a member of the OTZ program?**

There is something else that happened to you: no

I don't listen to anyone else, I only follow my own ideas, I only care about my mother and my brothers, whether they come from school or not.

Why?

Now, there is no such thing

What do they say when you are like this? They say many things to me

When I'm silent there are people who think that he is in love, but my mother is the only one who helps me, so she is silent. I'm an orphan, but I'm raised in an organization, so it doesn't seem like anything because you think so; I am growing up in an organization called S.O.S

**Who told you that you have the HIV virus/how did you know?**

The person I knew told me that he is not in Bahir-Dar, but I was going to ask them when I attended Shimbit Health Center. They told me themselves. They told me that they knew him when I was 14 years old. He wanted me to go there and ask him. As soon as I reached the door, he said, "I'm looking for you." Then I wasn't the only one there, because there were children growing up with me. We found out together. I didn't feel anything when they told me, and what surprised me was that I was told that this was at Shimbit Health Center.

**How did you feel when you were told that you have HIV?**

I remember saying that I would not take medicine at some point/time. It was from that I was brought here because they told me that I would not take medicine. My brothers told me that my neighbors would be here. Then they told me that you have your brothers in this, so I came here and they advised me.

**The reason why you said I won't take it**

The reason I said I don't take medicine is because I'm tired

Why don't I always take it? Why don't you tell me?

**Do you let others know that you are living with the virus? Have you tell yourself for others?** No they don't know

It is my mother who knows me, my guardian and the head of the organization, but no one else knows.

**Didn't you tell anyone else?**

I didn't tell anyone else

What will happen if I do not tell them if I take care of them, what will happen to them?

**Do you be careful that not to transmitted?** Yes

If you make yourself known, there are people who will dismiss you/you have been stigmatized

**Have you been stigmatized?**

No. They will do everything I ask because they think they can hear what they are thinking. Any of my brothers with the virus in their blood will do anything I ask. We have check-ups every year. If we feel something, we can change it.

**Does the virus have an impact on your education?**

There is nothing. When I said that I have received a scholarship, I have HIV in my blood.

How can I go? They said that there is no problem. I accepted it, but other children know me. I did not ask these brothers. How can you be silent?

Do you believe it is a scholarship?

They said nothing. They said nothing. They didn't ask me anything.

**Have you received a scholarship?**

Yes, I am going to Addis Ababa recently

They told me that there is no problem even after I have been checked

I will go to Ghana after an interview in Addis Ababa

My friends, how come you didn't ask them, shut up, where are you going to pass?

**I still have no problem and I haven't noticed any effect**

**What about on your behavior/feeling:** No

**Do you currently have a boyfriend/girlfriend? No**

**Do you have any idea of ​​getting a husband/partner in the future?**

God only knows about the future, we don't know the terms of tomorrow, so I can't tell you this.

I don't worry about it

**Do you have a plan to have a child?** No

**Regarding taking medicine,** it's convenient for me when I'm fasting because it's once a day, so I take it at one o'clock at night. My sister or mother is the ones who remember me. But I will take it myself.

When you take it in front of other people, do you take it without fear?

There is no problem with the family. But I don’t take in front of other people.

**From the library: I'm going out to drink water**

**Regarding the amount of medicine**, now I take only one tab, in the past I used to take a lot, but now it is much better and it is convenient for me in terms of my religion. I don't believe in food, I just take it; so far I haven't been under any pressure/burden

In the context where I was taking it twice a day I did not fast, because they did not allow me to fast, they used to give me medicine, but now I do not eat anything.

Where I used to take medicine twice a day, there was no fasting at that time. This was my challenge.

**Are there any side effects?** It is a new drug, it has it because it is difficult to get used to when it is new, for example, because I wake up at night, the taste/tone in my mouth is different and I don't like it.

**What are you going to do at night?**

There is study; Interviews are usually held at night, so I got up at night for the interview.

Now there is this flavor/tone, now I'm used to it.

**Things related to standing distance:** it is close, so I come with my feet

**Do you go somewhere else?** Yes, most of the time we go outside Bahir-Dar and we give them to our bosses, whether it's for children or older ones. For example, when I go to Addis Ababa, I explain it to them and give it to the boss as an object because other children shouldn't see it.

So that others don't see it, you give them to the boss saying yes/I will disappear. I don't care if others see it, we have a duty to give.

**Is there a gap compared to the services that the hospital is providing?**

It has been one year and six months since I started taking medicine here. Children accepted me immediately, I had a hard time getting used to them; I was only with my brothers, my brother is their boss, but the ideas he raised are very dear, they ask questions, I have to answer my duty, then slowly came to practice; Now we are like brothers and sisters with them. When I came out here, if you had seen me yesterday, I didn't have the courage to talk to them.

But now I have confidence, I participate in other meetings; I have the skills to go out and talk about the African Children's Day, and what is there in this organization?

Many brothers and sisters are shy, and even those who fight. We learn a lot of things and it's a cool thing to do

**Is there a gap from professionals?**

The service being provided is great for me

**Is there stigma/discrimination from society?**

When I was a child, no one knew me. I said it to myself, but my brothers and sisters asked why I was taking medicine. I don't think so because I don't know, but if I know, I don't think they care when they kiss. They thought I was sick and sleeping.

No one knows except the organization and my family.

**What type of support is needed for young people living with the virus?**

The different trainings are being offered here. I was going to participate in two separate trainings.

You think that the training that is being given is enough, because the chiefs are brothers, they tell me what training has been given and what is being given is awesome.

**Do you say that the support provided by the government is enough? But it is not enough**

For example, there are people who are economically disadvantaged, and apart from giving them soap/sanitary care, I think that other things should be provided for them, for example, if they provide psychological counseling services beyond this service.

**What is expected from the community**? It is enough if only counseling services are provided. If they know that it is the families and doctors who do everything for us, it is their duty to keep a secret.

If you have anything else you want to add, I lost my family because of the virus. They were able to leave because they knew. But no one can/shouldn't blame the family for saying that my mother is like this. It is my mother who made me pregnant. I believe that this is a matter of luck. It is from the point of view of what people have given us, not what we seek for ourselves

Some people say that the problem with the society is that when you wake up with anyone, they should not say that she has experienced such things. They don't know our background and our credentials and can't review us.

**Participant Six:**

**Describe what living with HIV means to you**

Living with HIV is just like living with a friend, that is, the medicine you always take with you is a habit you do every day, so there is nothing special about it.

**What do you feel when you think that the virus is in your blood**?

When my friends do something special, I feel something

**What things are different?**

When they have friends/lovers...well!...I wonder why I don't have friends, but I think I'm better than them anyway.

**Is there anything stopping you from getting a girlfriend?**

Besides, you can’t do what you want when you want/ because of the virus

**Do you have problems/challenges due to the virus?**

Things I have experienced: There are many things, at least when you are a woman, there are some pressures you face; you will be wanted, you will be raped, these same many men will make you wanted/ you will be sexually harassed/... I have experienced sexual harassment/ rape. .They all tried to strangle me but I diverted them.

**Who in what situation**

He wants me to be his friend, but I told him so many things that he wouldn't be me, and he couldn't understand me; I didn't want to talk at that time, I told him that I didn't because I preached that it was not the right time and time to talk. Then at the time I was talking, it was night and the neighbor was in another place and he didn't follow me. When I was about to enter the neighborhood, he abducted me. I got in / escaped /.. This is my biggest challenge

Another challenge gossip/rumour

What kind of rumour: If people find you here in the hospital while they are walking, what will you say to them. I don't say when I am going to take medicine; I tell them that I am going to ask someone. Then they look at your face and try to understand, but you ignore all that pressure and pass them off as a joke. This is the small challenge. Else...no

**Who told you that the virus is in your blood? /How did you know?**

I know that it is my family that makes me drowns. But when I came here, my family told me that it was the flu. The flu wouldn't go away. Then one day I came for a follow-up and people were talking about HIV. There are neighbors who take HIV with me. I thought about it, but there it is written as OH with my medicine card. I asked the doctor one day. What is this, Sister I said: This means HIV! I am an HIV user. She said she doesn't know where she is. When my family brought me, they told me she was going to take flu medicine. Then the sister replied that there is no problem with this medicine. At the time, it may have been because she wanted to not be surprised.

**Do you were surprised?** I didn't preach that I would have it, and I heard it six or seven years ago;

**What did you feel when you heard it?** I laugh and cry and it's the end of happiness. Although it made me cry at the time, I asked him how he came to be with me.

**Who did you ask? Who told you about my father?** He said, "No, ask the doctor. She told me that's the way it is. It's okay, and that's the medicine we take." She died. My father himself confirmed that you should not cut your medicine because there is a lot of loss of life there. You will have a happy life with the same family

I was in sadness for a long time because of this, I used to cry every day, then my father saw me one day and said, "Why are you crying?" I said to my father, why am I taking this medicine? My friend does not take it. After that, this is not a problem. Even if you take medicine, you will live a better life. He said that you should study and work like everyone else. You will live your life. You will live the same life as anyone else. I was happy when he said that because I knew that I can do everything. Before, we were taught that it was a disease for which there was no cure, so I thought it was everything that would kill me. Besides my father, my sister and my friends were on my side. My friend who is very close to me said that she called me so that I don't have to line up for medicine, she said to take me, it's too late, she said to me, the neighbor also called me and told me that there is a code or something, they will call me a policeman.

**What kind of code do you use?** The code can be whistle.

**Do you told yourself for others?** I told anyone/your friends that you are living with the virus. I didn't tell anyone but one of my friends knows my status.

**Why did you choose her:** Because we grew up together; she is with me.

What kind of reaction did she have after hearing it? After I told her, she loves me more and I love her more

**Why don't you tell for others?** So many things can happen to me.

When you are studying at school, do you think about the lesson or what are the children thinking? This teaching separates and disturbs the heart. I think that if my classmates know, they will talk about me

**Do you currently have a boyfriend?**

I have a friend, he is actually here with me, he knows himself and we will take the lessons he gives together

**Do you have started a sexual relationship?** we have not started

You will have a discussion about HIV transmission and ways of transmission; We know because we learn here and go.

**What kind of precautions have you taken for your future life, especially to give birth to a virus-free child?** We will recommend him for future career

As for the future life, we want to have a happy life

**Regarding taking medication**; It is always taken and when the time passes, it is woken up by an alarm; When the alarm goes off, I go home and take it

**Regarding the number of pills:**

I take two pills at night and I fast, and the amount does not bother me.

**How is the fear of taking in front of people?**  I do not take in front of people; I take medicine when I come from my room. If there is a stranger in the room, I tell them that it is for another disease, for example, I say it is medicine for the flu.

**What about the side effects of the medicine?** There is no other side effect except when you don't take it.

**Standing distance:** In terms of transportation; it’s not that much of a problem; sometimes when you receive it from family, it can be a bit overwhelming, but here it is given to us for taxi/transportation.

**Do you think it would be good if someone would support the adolescence?**

It would be great if there were different job opportunities for the youth. That is, if there are short courses, it can be educational or professional training.

Another thing is to make sure that there is no shortage of medicine if children who do not have the opportunity to get an education are able to get here and benefit from it.

**Regarding service delivery**: The service provided here is very cool and they take care of us like sisters, brothers and mothers in the professions; Of course, there are some disagreements, but there may be something that we can solve by ourselves, and there may be something that they can solve, we will solve it by caring for each other.

**Do you face Isolation and discrimination from society?**

In the past, it was widely seen because the community was not educated, so they think that even if you touch them, it will be passed on to them, but now they are being taught separately, so now they are isolated. Now this is the flu, HIV means the flu, what makes us feel ashamed is like a screw and pressure that make us quiet. Now I don't think I have to. The society's attitude is a little bit different. There is a tendency to isolate, especially the educated.

**Are there precautions you can take to ensure that the virus is not transmitted from you to others**?

Deciding and using prevention methods; learning; I don't want people to care if I bleed, because I don't want that person to run away after seeing the blood and touching the blood; But because you are human, an accident can happen.. Be careful at this time. If there are people nearby, they can tell you. Another thing is that when women go to Bajjaj, they can be naked and raped. That woman does not know what she is wearing. Why does he dare to do it against her will? The end of something against her will is not beautiful and she will be a victim. It means that both of them gave their lives.

**Regarding support:**

If it is supported by the budget, it would be great if there is something to motivate a company/organization so that the association can act independently.

**Participant Seven:**

**How would you describe living with HIV?**

Well, HIV has its own problems. It means thank you; the discrimination and isolation from the old society is decreasing. But it is not completely gone. Uuhhmmm..There are also problems.

When we talk about society separately in education, when we learn class separately, there is a topic that comes up in the area of ​​biology. At that time, there is an opportunity for students to consider a person with HIV as a human being, starting from my friend; Recently, my friend told me that if I had HIV, I would kill myself; I also understood this and tried to explain; Taking medicine that separates HIV from stress, cancer, and cancer every day is like pressure and I think HIV is a problem, it is only transmitted from person to person, that thing cannot be changed because the creator has given it once, and I don't think there is anything else that will cause you to spit yourself out. He said, "There is a big problem from the community. What can I tell you, It is very difficult. There is a lot left."

**What do you feel when you think about HIV?** I will never say that I am happy because I am not. But I accepted it and I am living by taking medicine.

*I won't tell you that I am ever happy, because I am, but I accept it and live by taking medicine.*

**What makes you unhappy?**  Because the virus is in my blood.

**What's wrong with you?**

Morally separate means that most of the time when I always think like this, I remember that there is always a separate topic of HIV in the biology book in every class. When the teacher explains it to us, one of them explains it well and the other one tells us to never read what he calls HIV, and he says something that is bad and something that will hurt you. There is a problem with the teachers. There is a problem with teachers, and when you hear that, sometimes you feel a psychological problem/pressure/.

**The challenge you faced:**

Medicine፡ we had a program with my friends and medicine. They put their things in my bag so they can take my bag at will, which is a very difficult test to carry medicine.

When they ask me to drink alcohol in ceremony/party, I refuse the alcohol at this time, they say something ‘’fara’’ and nothing. It means that they will exaggerate you with something else. Let my mind be troubled at this time.

**Have you introduced yourself to someone else?** No, they did not informed;

My family also knows that I am open with them that I will tell my friends elsewhere because I feel that they will avoid me if I tell them that I am living. That's why I kept quiet.

**When did you know you had the virus?** That is to say, they didn't even tell me, but the poster, the X sign. You will see you read a lot. I knew it myself, but when the doctor told me, "Do you know what medicine you are taking?" Yes, I told them it's HIV, no one told me when I came here, there is a poster, and everything is there. You see, you read, you always take it from the lesson. This is how I found out."

**How did you feel when you found out?** I mean, when I was a child, I didn't feel anything. When I grow up, things will come. I don't think I will come to this campus if the association doesn’t exist, because I think I'm the only one. I don't feel anything, God be praised. But now, after this association is established, you will have many followers and you will find the same freedom of leadership. When I heard it for the first time, it was my childhood and I was told that I thought so much from home. When I was a stubborn boy in the neighborhood, when we were playing games, she has HIV. Why are you playing together? That was the only time I felt threatened as a child. After I threatened, my mother kept saying nothing. There was every occasion where I was told that I was not looking at the store to buy something for myself.

**How did you feel when you were told like this**: I cried because I am a human being, no life will go on apart from this. Isolation and discrimination from the community is still existed.

**There is no pressure/impact due to the virus.** There is no difference in education.

**Do you have a boyfriend/lover?** No,

**What about the future?** I don't think about it now because I'm studying.

**What kind of husband do you want to have in the future/with or without the virus?**

It can't be without one because you can't be free from the debt of conscience. Even if he agrees, it’s very hard to make that person the damage that I am, so the only option I have is to find someone like me.

**Do you want to have a child in the future?** Yes, you don't believe me when I say that I never want to.

To have a child free of the virus what types of precautions you take?

When the time comes, I advise the doctors.

**Regarding taking medicine,** the current situation is very good. In the past, especially when it was a fast, when they said to sanctify a certain church, when I eat in the morning and take medicine, people always say something like, "Are you not fasting?" Now, whether it is praised or not, once it is taken away, I will always fast with my friends. It has been a year.

**Regarding taking medicine without fear in front of people:**

There is no problem in front of the family, because they all know it, but I will take it carefully in front of other people.

**What kind of precautions do you take?** There is a rubber band, because it is useless to hold medicine in paper, it prevents heat and cold. I will do that from the time I go out and take it.

**There is a side effect associated with the drug**: no harm

**In relation to the distance from the institution**, it does not bother me that much

**What is the attitude of the community?** The community is not segregated like it used to be, not even now, but if they know that I have to be segregated at school, it is very difficult. I will give it because I am living there and I know everything and it is very difficult. One of them even said that my mother gave up on a person with HIV.

**Who should do what?** You say, if the working conditions are favorable, if an organization is opened, it would be good if I could work here in our country when I finish school.

**Secondly**, I think it's a problem. When we enter here, if we put up a poster that says ART / if there is text or if it is in a place where we turn around / the guards and others will see us when we enter. .. It does not provide comfort.

They understand that since they are all educated, it is known that we will come to take HIV medicine.

**What kind of place does it lead to?**

I mean, even if you take it from the hospital, there is a place far away from the guards, it is good if it is in a place where a person who is being treated while sleeping can be reached...but here you can also find people from the neighborhood. ..This blood poster is also there, so you can understand it by reading it.

**How did you feel when you found out?** I mean, when I was a child, I didn't feel anything. When you grow up, things will come. I don't think I will come to this campus if the union exists, because I think I'm the only one. But now, after this association is established, you will have many followers and you will have the same freedom of thought.

When I heard it for the first time, it was my childhood and I was told that I thought so much from home. When I was a stubborn boy in the neighborhood, when we were playing games, he had HIV. Why are you playing together? That's the only time I felt threatened as a child. My mother kept saying nothing.

How did you feel when you were told like this: There was every occasion where I was told that I was not looking at the store to buy something for myself. I cried because you are a human being, no life will go on apart from this. There is isolation and discrimination from the community

There is no pressure/impact due to the virus. There is no difference in education

Do you have a boyfriend/lover? No, what about the future? I don't think about it now because I'm studying. I don't think about it, but in the future, because I'm always human, it doesn't mean that I don't want that thing. It would be good if he kept his time. I don't have such an idea now, but it would be nice if he kept his time and everything.

What kind of husband do you want to have in the future/with or without the virus? It can't be without one because you can't be free from the debt of conscience. Even if he agrees, it's very hard to make that person the damage you are.

**Do you want to have a child in the future?** Yes, you don't believe me when I say that I never want to

To have a child free of the virus and how to prevent pregnancy / precautions

When the time comes, I advise the doctors

Regarding taking medicine, the current situation is very good. In the past, especially when it was a fast, when they said to sanctify a certain church, when you eat in the morning and take medicine, you always say something like, "Are you not fasting?" Now, whether it is praised or not, once it is taken away, I will always fast with my friends. It has been a year.

**Regarding taking medicine without fear in front of people:**

There is no problem in front of the family, because everyone knows it, but I will take it carefully. What kind of precautions do you take? There is a rubber band, because it is useless to hold medicine in paper, it prevents heat and cold.

There is a side effect associated with the drug: no harm

**In relation to the distance from the institution,** it does not bother me that much

What is the attitude of the community? The community is not segregated like it used to be, not even now, but if they know that I have to be segregated at school, it is very difficult. I will give it because I am living there and I know everything and it is very difficult. One of them even told me that my mother has given up on a person with HIV.

**Who should do what?**

You say, if the working conditions are favorable, if an organization is opened, it would be good if I could work here in our country when I finish school.

Second, I think it is a problem. When we enter here, if there is a poster that says ART / there is text or it is somewhere around it / the guards and others will see us when we enter. It is known that we will come, even if I don't like it..it will not give comfort

They understand that since they are all educated, it is known that we will come to take HIV medicine.

What kind of place does it lead to? I mean, even if you are taken from the hospital, there is a place far away from the guards. It is good if it is in a place where a person who is being treated while sleeping can be reached. But here you can also find people from the neighborhood.

**What kind of method do you use to deal with the pressure caused by the virus?**

Saying that I will be emotional on time is always a prayer; it is difficult to say that there is no disease.

Sometimes you forget that when you come here once a month, when you hear this word, it means that when you are always hanging out with friends at school, when the virus starts up and spreads, that's when you hear it. Yes, it can be removed now. I have nothing. When he examines me, he says you don't need the result. Let's see. I have nothing.

Let's see what I have to say. I'm happy there. I hear that he doesn't spit out anything. It's hard when you're two and a half. When I hear him here, the result can be like this. You can't be sure. I also admitted that I had to.

When someone who doesn't know me talks about HIV at home, I go out

Whether I want to see a movie about HIV on TV or not, I will not watch it.

I get married and have children? There is a big gap in understanding that they can marry those who are similar. Oh well, later on, they will say that I am not married and I will not have children. There is no hope for them. There are those who want to see their children grow up one by one, make wealth and reach a great position.

**How did the virus affect adolescents?**

One is that their age is when they think a lot about their future dreams.

It has a special effect on their future life; it will make them forget about it. As in the context of Ethiopia, it is possible for those who get nutritious food; this is the isolation and discrimination that they do not grow up like their peers, because they are worried. So the effect is heavy depending on their age.

**Participant: 11**

**If you make it clear to me what it means to live with HIV**

Living with HIV means I'm happy because I don't think I brought it. on nothing has happened to me as long as I am at peace with my family and the society as well. There is nothing different about living with the virus.

**Are you feeling sick because of the virus?**

Yes, sometimes when I think about the HIV situation; when my friends went to other places to entertain her, I used to think that I would not be like them because I think I have HIV/AIDS.

Because of this, I did not go to school. I mean, she was my best friend and she used to make fun of me, I mean, I didn't go to class because she said you are a slave...I think everyone has heard about it and I don't go to school because I'm afraid of it. I thought it was just me, but it wasn't.

**Do you have any challenges due to the virus?**

But at some point I stopped taking medicine for six months.

I had a fight with my family and quit. After six months, they called me and came and started medicine. When I started, my hand hurt and my body started to hurt. This is the pressure I got due to the virus.

**Who told you that you have a virus in your blood?** My father told me when I was 11 years old

When I came here and asked them what medicine to take, they said that it is better for malaria, then the doctor told her to tell her, and then the doctor said, "Which one is better than DM and HIV? I don't think DM is any good, but I told her that you have HIV. It doesn't mean anything. After I heard from her, my father also told me."

**How did you feel when they told you that you have HIV in your blood?**

I was shocked/I felt shocked/I cried. One of my friends didn't say anything.

Who told him: I thought he was from the family; she knows when I know

After that, I did not stop taking medicine

**Have you disclose/Introduce yourself to others**: everyone in our neighborhood knows about it

I am the one who told them because they are borrowing a lot from me so that they should be careful about what they transfer.

**After you informed them, what kind of kindness did they show you?** They didn't isolate me, they didn't stay away from me

**Why did you choose these people፡** because I want them not to be like me and because I want them to be careful. Even before they know it, It is the same in behavior / it doesn't change / we are closer to my friend

**Has the HIV virus affected you?**

Yes, I am worried. Why, if I have something on my hand, I will take revenge on everything that doesn't offend me. Because I think I am inferior because I have the virus and because they are the ones who infected me with the virus.

Distinguish by education. I had dropped every single lesson.

**For what;** I was afraid because they might laugh

But now this does not exist

**Do you have a boyfriend/lover**? No

**What do you think about the future?**

I don't think why because I am a user

If you want to marry a future husband, what kind of husband would you choose?

Kind of like me I want to have a child free of the virus

**Regarding taking medicine**: I used to take medicine before, I take it when I feel happy, I leave it when I feel unhappy, but now I have fixed it. Now I have started taking medicine regularly since 2015.

**Why**: If someone/family/ avoids me, I won't take it...

**Number of medicines**: Before, they give me a lot, so I get tired of it...so I don't take it. Because of this, I don't take it.

**Do you take medicine without fear in front of people?**

No, I will not take it. If a guest comes from our house, I will not take it, I will leave it

**Why are you afraid?** I don't want them to see me

**Why don't you take it when you go to your room or go out to urinate?** : No, I won't take it, my family think I will take it and they don't see me.

It's because I think people don’t come back to our house.

**Side Effects**: None

But when I stop taking medicine, I feel sick and tired.

**What kind of method do you use when the medication time is up?**

Since there is a program of kana movie on TV at one hour, when the program starts, I will set it aside for one hour; otherwise I will use a phone clock or a wall clock.

**Participant 12:**

**Explain what living with HIV means to you**

I think that it is possible to live like any other person, except to swallow the medicine

**When you think about the HIV situation, what do you think**? If they think different from those around them, I don't feel that much, but I feel that I am different from them.

**How are you different?** Only by swallowing medicine

**Challenges faced:**

There are no challenges and no pressure

**How did you find out you are living with the virus?** It has been with me since I was a child. When I grew up I was 12 years old, they told me that I don't have a mother and father. They are my guardians and I live with my mother's sister's husband.

How did you feel when they told you? I didn't feel anything

**Do you have made yourself known to others?**

There are those who isolate me. It varies from person to person

Before, those who knew me well did not exclude me, but it varies according to people's views

The impact/pressure you have had on your education: None

**Do you have a female friend? No**

**Do you have a plan for the future?** It is according to God's will

**What kind of wife do you think you want to marry?**

If she have the virus, who have a good attitude

**Regarding taking medicine;** I take medicine on time. I don’t stop it.

Number of pills: I don't mind, I take two pills a day and one at night

**Side effects:** None

How about taking medicine in front of people?

If they ask me, I will tell them

**About the distance of the institution**, I am a little worried about the cost of transportation. He/guardian/ is the one who gives me trouble.

**In terms of discrimination and isolation, have you received from your friends in the community/school?**

I don't think they will deliver to me because they don't know

**Is there a gap in the service provided by the institution?**

There is no gap; they will help us a lot

**Regarding support**: It would be good if the relevant body was asked and provided with food and hygiene materials. It is good if they support what they can.

Other: No

The virus does not have that much effect, but it has a good effect

**The family also knows**: there is no discrimination or isolation

I am careful not to spread the virus from me to them.

Participant 13

How do you explained to me what it means to live with HIV

For me, living with HIV is just like anyone else's, now that I'm out of it.

The same life is the same life as a virus-free person. But when he told us at the beginning, his feeling was a bit shocking. I thought that I was different from other people. But it is better now.

**How do you feel when you think about the HIV situation?**

When I was told for the first time, I was very shocked. It was a matter of despair. It was scary when I saw the isolation and discrimination of the society.

Are there any challenges/stress caused by the virus? I have an experience with this

Yes, incidentally, I work in sports/circus. There is something that happened to me in this situation, that is, I had a chance to go outside and when I was tested, I had HIV, so I didn't go.

Because of this, I was faced with the possibility of going to England... This situation was so bad for me that I stopped taking medicine and sports. Nothing happened to me because of the virus, and I have friends, so I talk to them about medicine and other things.

**How do you know if you have HIV in your blood?**

They found out that I had the virus when I was ten years old. I was first told by the doctors, then my father told me... when I was told, my father was also there.

What is the situation you were told?

It was very shocking. My father used to communicate with people like … Sports House and when my father told me that you take medicine to give you strength for sports. He tells me that you take it as an incentive.

It was my father who introduced me to sports. He tells me that if the disease persists and take medicine, do sports/circus/be good. I was shocked when I was told that this was changed to an HIV drug.

After hearing that, I stopped taking the medicine. Even though my father told me to take it, I couldn't listen; At this time, I was very good at the circus.. For this reason, I thought about changing the country, I also thought about not being there... Because of this, I stopped taking medicine for six months.

How did you feel when you stopped taking the medicine?

Feeling dizzy. I don't study properly; When I do sports, I can't work as well as my friends; When I do things that require balance, it makes me dizzy, I have to sit down immediately, insomnia, anxiety, sweating. I had a general impotence with that rash....all of which paid off within three weeks of stopping the medication.

How is this thing fixed?

After all, what do you call yourself?

I started listening to myself and here I had a paper to write. When I came here and asked the nurse, she told me that your father said, "Why are you treating me like this?" She advised me.

There were other children who were about to start an association at this time, so they said to me, why don't you start with these children? I arrived on Thursday and the association started on Saturday. When it started, it was six/seven years ago. They advised me to become a member of the association. When I came on Saturday, I was relieved to see others, they knew I was the only one.

Have you introduced yourself to others?

I told two of my best friends; I was not told about the other one

**What was their reaction after you told them?**

At first they were shocked...then they told me that I was caught when I was born with the family, and they consoled me by saying that this is not your fault...Also, we have a better relationship...they did not leave me.

**How did you choose these?** Yes, from our upbringing

Because they are my co-parents, we are together, as we said, in school and in every neighborhood we go to. I was not discriminated against

Why didn't you tell others?

There is something I haven't said to others, isn't it, to say to myself: When you see a person's point of view, a person's point of view is something that doesn't matter.

When I tried to tell him one of the genres, you don't tell someone directly, you try different things. I believe it's better to try something like this before saying something like that.

You don't need to approach him like this when you start. If he says that, you don't need to say it. If you tell him, then something worse will happen.

I have not experienced any discrimination or exclusion after introducing myself.

There is discrimination/isolation that they heard from people without you telling them

Yes, when you said that, I remembered something. It's a separate school. When I was in the tenth grade of Hail School, there was a blood donation in every school and during that blood donation, all the students of the class happened to be there. What I was and it was the last pressure/influence/

Among them, there is a boy from the village who has blood and how did he know, he knows, he was told to others, he was told to benefit me, don't push him, why did they say he can't donate? Something happened, didn't it? The education situation didn't go well for me.

But at that time, I was a little better because I had already taken a discussion class. My education itself was different in the ninth grade. It was nothing to get a grade in the top ten, but it was after this that I came second. They strengthened me, but there was no change. From the community: From the community, I don't spend much time at home. I spend it in classes. I spend it in the library. I don't see much. I only go to game zones and I'm at home for sports. I go to sports for a long time. If I come in at eight o'clock, I don't leave until twelve o'clock. If I come in in the morning, I stay until nine o'clock.

From the family: I have an elder and a younger, they are free, they have my mother and father

My elder brother remembers me and the younger one tries to come running. It's not a family that upsets me. My father sometimes forgets and goes to sleep early

**Has the virus affected you academically or socially?**

After I found out, it was okay to stop studying, to stop taking medicine/not to take it at all.

**Do you currently have a girlfriend/boyfriend?** I don't have one yet.

What do you think about the future? When will you become a human being, if you don't tell me that, I will think about that for the rest of your life, but there is something that is stopping me. Not only that, but you isolate yourself from people. This was the thing before. After we started here, when you found out that there are people, I just realized that there no is another thing that limits you. That means you can have a girlfriend.

I think your girlfriends viral status must be the same as mine

What do you think about HIV transmission and prevention methods?

I think it's better to separate from the broadcast now. I think it's better to separate now and people have a little recognition. If you think about it a little bit, we were also watching it for a while. We have noticed a slightly different way of distribution in the areas where grocery stores are concentrated.

How did that happen? Especially in pubs, people are dependent on alcohol because HIV is usually transmitted through unprotected sexual intercourse. It is better than before because there is an organization that supports this.

Talk about how you are taking your medication.

First of all, I am coming on Saturday, I will take the medicine from the health facility. After I take it, I have an alarm every day, it always goes off at two o'clock and fifty-seven minutes (2:57). I will take it on time even if it is not late because I have a strong faith to live.

Even if I forget it, my brother can forget it

As for the number of pills: I take one fruit and it doesn't bother me

Rather than taking medicine in front of people, many times I go to the conditioning/field, that is, when I go to a sports competition/show. Today I think I have a cold, there is no one who is clear, using such things, today I am fooling myself, I am going to take paracetamol, I think I have a cold. I am not saying that it is an HIV medicine.

As for the side effects: If I take it, there is

There are no side effects while taking the medicine, but if I stop taking it, I get headaches, insomnia, and if I don't take it for a day, I get depressed the next day.

Regarding the lack of service/satisfaction on the part of the institution

I must mention that the meeting with the health institution is on Saturday, and we will not have classes and there will not be much work. After we come here, we have transportation, but it is small, and he will bring us back.

Who else should give what kind of support, you say?

I can't say who said this, but I think that everyone has an obligation. I think that every organization, every institution that works around this should give all the support/attention. There are those who have acquired a lot of knowledge, and there are those who have reached the level of bedridden and even death.

This is because the organizations are not coming forward to find out why this is happening / these children need to get support. I don't think it's enough to just take medicine, they should get different trainings, they should also get life skills, how to promote the things related to drugs, discrimination and stigma, I believe it would be better if they were able to do that if they were able to do that.

There is a gap that can be seen by the experts

If you fix it, I will tell you two things

After some children come here, counseling services are not only given to them, but if they are followed up every month...take it today/this month and ask how much medicine you have not taken. My life schedule He may forget while watching a movie, so if he is told a program / solution for this... he should not be told to take medicine... I think it would be great if he could be advised to arrange the time... There is a small gap in providing this kind of service.

Second, as I told you earlier, when you go to any place like the health office, Canal Plus, AMSD, and social life, they will give you the answer that if you are separated from social life, we will be supported. We have not received an answer. I think there is a problem in the middle. Something said to have been sent from the company does not arrive here. I think this is a problem with the doctors. The other thing is that children should be dealt with carefully because, as I told you earlier, there are children who are becoming bedridden and died. This thing is happening when the mind of a human being works on it separately, when you explain to him what is about medicine, it is like eating food if he understands it, and he eats food because he understands the benefits. It is not enough to send him medicine just because he came here.

They should receive adequate training, conditions should be favorable for them, exchange of experience from different countries, for example, there are various organizations like Dese፣Kombolcha, there are associations. It is possible Even if you are doing this, I would say that it would be good if the different bodies work closely around drug addiction.
