# Supplementary material for: The experience of living with human immunodeficiency virus among adolescents at Felege Hiwot Comprehensive Specialized Hospital Bahir-Dar, Northwest Ethiopia, A phenomenological study
Source: PLoS One. 2025 Jan 9;20(1):e0308347. doi: 10.1371/journal.pone.0308347 (PMC11717265; doi:10.1371/journal.pone.0308347)
Supplement: Attachment — Submitted filename: plosone-reviewer-edited form.pdf [file pone.0308347.s005.pdf]

Recommendation: No Recommendation

Cancel | Save & Submit Later | Upload Reviewer Attachments | Proof & Print | Proceed

Reviewer Instructions

Thank you for agreeing to contribute your expert opinion to the evaluation of this manuscript.

Assessing the manuscript

As you evaluate the manuscript, consider whether it satisfies the *PLOS ONE* criteria for publication. Consult these resources for additional information:

- Reviewer Guidelines. An overview of our peer review process and what to consider in your review.
- PLOS Reviewer Center. Peer review how-to information and strategies.
- Other questions? Contact us at plosone@plos.org

Submitting your review

To complete your review, answer the questions in the review form below. (A red asterisk (*) indicates that a response is required.) Enter the main body of your review under question 5, along with any concerns about potential competing interests on the part of the authors, data availability, or research ethics. Declare your own potential competing interests under the section titled Confidential to Editor. This information is visible only to the editor and the journal office.

Review Questions

***Comments to the Author**

Insert Special Character

1. Is the manuscript technically sound, and do the data support the conclusions?

The manuscript describe a technically sound piece of scientific research however there is insufficient data that supports the conclusions. furthermore, there is no conclusion drawn in this manuscript.

Please select a response

---

*2. Has the statistical analysis been performed appropriately and rigorously?

Please select a response

---

*3. Have the authors made all data underlying the findings in their manuscript fully available?

Authors have presented their findings well, however, there is not technicality and it lacks scientific principles of writing.

Please select a response

---

*4. Is the manuscript presented in an intelligible fashion and written in standard English?

The manuscript rs presented in standard English, however, there is grammatical errors throughout

Please select a response

*5. Review Comments to the Author

This manuscript present a significant concept and they have researched it adequately, however i have noted the following:

The introduction does not address the topic- it is generalized to the HIV positive community, adolescents are only mentioned as an example not the main focus and also does not provide the overview of the problem- introduction should quantify the problem, globally and local

It do

Character Count: 0

also present the current status of the problem, currentinterventions and the gaps identified- reason for this work. less literature was cited and also lacks scientific element throught the manuscript

*6. PLOS authors have the option to publish the peer review history of their article (what does this mean?). If published, this will include your full peer review and any attached files.

If you choose "no", your identity will remain anonymous but your review may still be made public.

Do you want your identity to be public for this peer review? For information about this choice, including consent withdrawal, please see our Privacy Policy.

Yes

Response required
Your full name*, as it should appear in the published peer review:

Rirhandzu Mabasa. (Limit 2 to 20000 Characters)

Character Count: 0

***Confidential to Editor**

1."I have no competing interests.

Character Count: 0

2. Did you receive any assistance in preparing this review (e.g. from a post-doc or graduate student)? No

Character Count: 0

3. If accepted, do you think this submission should be highlighted on the PLOS ONE website? PLOS ONE does not evaluate manuscripts based on perceived significance or readership. We aim to provide tools for readers to filter and evaluate our publications. (optional)

Please select a response

ORCID Reviewer Credit

Note: This reviewer's default response is 'No' so this Review will not be sent to ORCID; you cannot change this as you are proxied in for this person. The Reviewer may change this after you submit the review, by going to their Completed Reviewer Assignments folder and using the 'ORCID Deposit Authorization' action link. They must log in to do so - you cannot do that by proxy.

Cancel | Save & Submit Later | Upload Reviewer Attachments | Proof & Print | Proceed
