# Supplementary material for: The experience of living with human immunodeficiency virus among adolescents at Felege Hiwot Comprehensive Specialized Hospital Bahir-Dar, Northwest Ethiopia, A phenomenological study
Source: PLoS One. 2025 Jan 9;20(1):e0308347. doi: 10.1371/journal.pone.0308347 (PMC11717265; doi:10.1371/journal.pone.0308347)
Supplement: Attachment — Submitted filename: Plos one Article for review.docx [file pone.0308347.s004.docx]

**PLOS ONE**

**The Experience of Living with Human Immunodeficiency Virus among Adolescents, A Phenomenological Study**

--Manuscript Draft--

| **Manuscript Number:** | PONE-D-24-01002 |
| --- | --- |
| **Article Type:** | Research Article |
| **Full Title:** | The Experience of Living with Human Immunodeficiency Virus among Adolescents, A Phenomenological Study |
| **Short Title:** | The Experience of Living with Human Immunodeficiency Virus among Adolescents, A Phenomenological Study |
| **Corresponding Author:** | Bazezew Asfaw Guadie, BSC ,MSC in Pediatrics and Child Health Nursing Amhara Regional Health Bureau: Amhara National Regional Health Bureau Bahir Dar, ETHIOPIA |
| **Keywords:** | Lived experience, phenomenology, adolescents, human immunodeficiency virus |
| **Abstract:** | Background  Living with the human immunodeficiency virus is challenging for individuals, including adolescents, due to stigma and discrimination by society in addition to the physical effects of the disease. The previous studies in Ethiopia were more inclined toward the quantitative aspect of this problem. But the lived experiences of adolescents living with the human immunodeficiency virus were not well investigated before in Ethiopia.  Hence, exploring the lived experience of adolescents living with the human immunodeficiency virus is crucial to overcoming the actual problems they face.  Objective  To explore the lived experiences of adolescents living with human immunodeficiency virus at Felege Hiwot Comprehensive Specialized Hospital.  Method  A phenomenological approach was conducted among thirteen purposively selected adolescents at Felege Hiwot Comprehensive Specialized Hospital, Bahir-Dar, northwest Ethiopia, from March 25 to April 30, 2023. The sample size was determined using the information saturation technique. An in-depth interview technique using a semi-structured open ended interview guide was used to collect data. An audio recorder was used to capture the conversation. Verbatim data transcription was used. The transcribed data were translated from the Amharic language to English using the conceptual translation method. The translated data was imported to Atlas.ti.8 software for further analysis. The inductive thematic analysis technique was done with illustrative quotes to present findings. To ensure the quality of the data a debriefing, member checks, and feedback from colleagues were employed.  Result  Findings were summarized under four main themes (lived with burdens of immunodeficiency virus, disclosure, ART adherence, and future aspiration) with eleven sub- themes. The participants expressed how challenging it has been for them to live with the human immunodeficiency virus due to social issues as well as emotional and psychological problems. They hid their condition from others due to concerns about stigma and discrimination.  Conclusion  Adolescents living with the human immunodeficiency virus experienced numerous psychological, social, emotional, and adherence challenges. Persistent health education for the community is recommended to avoid stigma and discrimination against adolescents with the virus. Keywords: Lived experience, phenomenology, |

*Powered by Editorial Manager® and ProduXion Manager® from Aries Systems Corporation*

|  | adolescents, human immunodeficiency virus |
| --- | --- |
| **Order of Authors:** | Bazezew Asfaw Guadie, BSC ,MSC in Pediatrics and Child Health Nursing |
|  | Yinager Workineh, BSc, MSC, PhD student |
|  | Minyichil Birhanu Belete, (BSC N, MSC PHN, MPH in Epidemiology, Assistant P |
|  | Meseret Mekuriaw Beyene, BSC, MSC |
|  | Tarik Yenew Tessema, BSC, MSC |
|  | Ayalew Kassie Melese, BSC, MSC |
| **Additional Information:** |  |
| **Question** | **Response** |
| **Financial Disclosure**  Enter a financial disclosure statement that describes the sources of funding for the work included in this submission. Review the [submission guidelines](http://journals.plos.org/plosone/s/submission-guidelines#loc-financial-disclosure-statement) for detailed requirements. View published research articles from [*PLOS ONE*](https://journals.plos.org/plosone/) for specific examples.  This statement is required for submission and **will appear in the published article** if the submission is accepted. Please make sure it is accurate. | The author(s) received no specific funding for this work. |
| **Funded studies**  Enter a statement with the following details:   - Initials of the authors who received each award - Grant numbers awarded to each author - The full name of each funder - URL of each funder website - Did the sponsors or funders play any role in the study design, data collection and analysis, decision to publish, or preparation of the manuscript? |  |
| Did you receive funding for this work? |  |
| **Competing Interests**  Use the instructions below to enter a competing interest statement for this submission. On behalf of all authors, disclose any [competing interests](http://journals.plos.org/plosone/s/competing-interests) that could be perceived to bias this work—acknowledging all financial support and any other relevant financial or non- | The authors have declared that no competing interests exist |

*Powered by Editorial Manager® and ProduXion Manager® from Aries Systems Corporation*

| financial competing interests.  This statement is required for submission and **will appear in the published article** if the submission is accepted. Please make sure it is accurate and that any funding sources listed in your Funding Information later in the submission form are also declared in your Financial Disclosure statement.  View published research articles from  [*PLOS ONE*](http://journals.plos.org/plosone/) for specific examples. |  |
| --- | --- |
| **NO authors have competing interests**  Enter: *The authors have declared that no competing interests exist.*  **Authors with competing interests**  Enter competing interest details beginning with this statement:  *I have read the journal's policy and the authors of this manuscript have the following competing interests: [insert competing interests here]* |  |
| * typeset |  |
| **Ethics Statement**  Enter an ethics statement for this submission. This statement is required if the study involved:   - Human participants - Human specimens or tissue - Vertebrate animals or cephalopods - Vertebrate embryos or tissues - Field research   Write "N/A" if the submission does not require an ethics statement.  General guidance is provided below. Consult the [submission guidelines](http://journals.plos.org/plosone/s/submission-guidelines#loc-guidelines-for-specific-study-types) for | Ethical approval was obtained from the Institutional Review Board of Bahir Dar University College of Medicine and Health Sciences with an ethical approval protocol number of CMHS/IRB/01/757/2023 and support letter was obtained from Amhara Public Institute for study site. After submission of the permission letter for ART coordinator the discussion with health care providers at ART clinic was done about selection of participant. after taking assent from adolescents , consent from family or guardians was taken and each and every information taken from participant kept secret. |

*Powered by Editorial Manager® and ProduXion Manager® from Aries Systems Corporation*

| detailed instructions. **Make sure that all information entered here is included in the Methods section of the manuscript.** |  |
| --- | --- |
| **Format for specific study types**  **Human Subject Research (involving human participants and/or tissue)**   - Give the name of the institutional review board or ethics committee that approved the study - Include the approval number and/or a statement indicating approval of this research - Indicate the form of consent obtained (written/oral) or the reason that consent was not obtained (e.g. the data were analyzed anonymously)   **Animal Research (involving vertebrate animals, embryos or tissues)**   - Provide the name of the Institutional Animal   Care and Use Committee (IACUC) or other relevant ethics board that reviewed the study protocol, and indicate whether they approved this research or granted a formal waiver of ethical approval   - Include an approval number if one was obtained - If the study involved *non-human primates*, add *additional details* about animal welfare and steps taken to ameliorate suffering - If anesthesia, euthanasia, or any kind of animal sacrifice is part of the study, include briefly which substances and/or methods were applied   **Field Research**  Include the following details if this study involves the collection of plant, animal, or other materials from a natural setting:   - Field permit number - Name of the institution or relevant body that granted permission |  |
|  |  |
| **Data Availability**  Authors are required to make all data underlying the findings described fully available, without restriction, and from the time of publication. PLOS allows rare | No - some restrictions will apply |

*Powered by Editorial Manager® and ProduXion Manager® from Aries Systems Corporation*

| exceptions to address legal and ethical concerns. See the [PLOS Data Policy](http://journals.plos.org/plosone/s/data-availability) and [FAQ](http://journals.plos.org/plosone/s/data-availability#loc-faqs-for-data-policy) for detailed information.  A Data Availability Statement describing where the data can be found is required at submission. Your answers to this question constitute the Data Availability Statement and **will be published in the article**, if accepted. |  |
| --- | --- |
| **Important:** Stating ‘data available on request from the author’ is not sufficient. If your data are only available upon request, select ‘No’ for the first question and explain your exceptional situation in the text box. |  |
| Do the authors confirm that all data underlying the findings described in their manuscript are fully available without restriction? |  |
| **Describe where the data may be found in full sentences. If you are copying our sample text, replace any instances of XXX with the appropriate details.**   - If the data are **held or will be held in a public repository**, include URLs, accession numbers or DOIs. If this information will only be available after acceptance, indicate this by ticking the box below. For example: *All XXX files are available from the XXX database (accession number(s) XXX, XXX.*). - If the data are all contained **within the manuscript and/or Supporting Information files**, enter the following: *All relevant data are within the manuscript and its Supporting Information files.* - If neither of these applies but you are able to provide **details of access elsewhere**, with or without limitations, please do so. For example:   *Data cannot be shared publicly because of [XXX]. Data are available from the XXX Institutional Data Access / Ethics Committee (contact via XXX) for researchers who meet the criteria for* | most of the data are included in manuscript but some of the data like audio records, transcription and translations are restricted for the sake of ethical issue. The supporting information and other data will be available via corrospondence email adress |

*Powered by Editorial Manager® and ProduXion Manager® from Aries Systems Corporation*

| *access to confidential data.*  *The data underlying the results presented in the study are available from (include the name of the third party and contact information or URL).*   - This text is appropriate if the data are owned by a third party and authors do not have permission to share the data.   * typeset |  |
| --- | --- |
| Additional data availability information: |  |

*Powered by Editorial Manager® and ProduXion Manager® from Aries Systems Corporation*

[Click here to access/download;Manuscript;Manuscript file.docx](https://www.editorialmanager.com/pone/download.aspx?id=35177391&guid=159c042c-baca-45de-a050-d8c422f3bb8f&scheme=1)

### 1 The Experience of Living with Human Immunodeficiency Virus among

2 **Adolescents at Felege Hiwot Comprehensive Specialized Hospital Bahir-Dar,**
3 **Northwest Ethiopia, A Phenomenological Study**

#### 4 Bazezew Asfaw Guadie1*, Yinager Workineh2, Minyichil Birhanu Belete2, Meseret

5 **Mekuriaw Beyene1, Ayalew Kassie Melese1, Tarik Yenew Tessema3**
6 **1 Department of Nursing Bahir Dar Health Science College, Bahir Dar Ethiopia**
7 **^2^Department of Pediatrics and Child Health Nursing, School of Health Sciences, College of**
8 **Medicine and Health Sciences, Bahir Dar University, Bahir Dar, Ethiopia**
9 **^3^Infectious Disease Screening, Bahir Dar Blood Bank Office, Amhara National Regional**
10 **State Health Bureau, Bahir Dar Ethiopia**
11 *** Corresponding author**
12 **Email:** [**bazezewasfaw@gmail.com**](mailto:bazezewasfaw@gmail.com) **(basfaw929)**
13 **Abstract**
14 **Background**: Living with the human immunodeficiency virus is challenging for individuals,
15 including adolescents, due to stigma and discrimination by society in addition to the physical
16 effects of	the disease. The previous studies	in	Ethiopia were more	inclined toward the
17 quantitative aspect of this problem. But the lived experiences of adolescents living with the
18 human immunodeficiency virus were not well investigated before in Ethiopia. Hence, exploring
19 the lived experience of adolescents living with the human immunodeficiency virus is crucial to
20 overcoming the actual problems they face.
21 **Objective**: To explore the lived experiences of adolescents living with human immunodeficiency
22 virus at Felege Hiwot Comprehensive Specialized Hospital.
23 **Method**: A phenomenological approach was conducted among thirteen purposively selected
24 adolescents at Felege Hiwot Comprehensive Specialized Hospital, Bahir-Dar, northwest
25 Ethiopia, from March 25 to April 30, 2023. The sample size was determined using the
26 information saturation technique. An in-depth interview technique using a semi-structured open
27 ended interview guide was used to collect data. An audio recorder was used to capture the
28 conversation. Verbatim data transcription was used. The transcribed data were translated from
29 the Amharic language to English using the conceptual translation method. The translated data
30 was imported to Atlas.ti.8 software for further analysis. The inductive thematic analysis
31 technique was done with illustrative quotes to present findings. To ensure the quality of the data
32 a debriefing, member checks, and feedback from colleagues were employed.
33 **Result**: Findings were summarized under four main themes (lived with burdens of
34 immunodeficiency virus, disclosure, ART adherence, and future aspiration) with eleven sub-
35 themes. The participants expressed how challenging it has been for them to live with the human
36 immunodeficiency virus due to social issues as well as emotional and psychological problems.
37 They hid their condition from others due to concerns about stigma and discrimination.
38 **Conclusion**: Adolescents living with the human immunodeficiency virus experienced numerous
39 psychological, social, emotional, and adherence challenges. Persistent health education for the
40 community is recommended to avoid stigma and discrimination against adolescents with the
41 virus. **Keywords**: Lived experience, phenomenology, adolescents, human immunodeficiency
42 virus

43

44

# 45 Introduction

46 The lived experience of people living with human immunodeficiency virus (PLWH) is a
47 challenge or difficulty that individuals have faced in their life span due to being victims of
48 human immunodeficiency virus (HIV) infection (1). Some of the challenges, and difficulties
49 people faced due to having an HIV infection were cognitive, physical, psychological, social,
50 and information seeking. The lived experiences of people living with HIV faced problems
51 such as emotional shock, the fear of the consequences, the feeling of guilt, discouragement,
52 and the escape from reality (2). For instance, emotional shock is one of the unpleasant events

that lead to loathing and social isolation after they are diagnosed HIV positive (2).

53 Adolescence is the phase of life between childhood, and adulthood, from the ages of 10 to
54 19years (2), and it is a unique stage of human development and an important time for laying
55 the foundations of good health. Adolescents and young adults represent an increasing
56 proportion of people living with HIV globally (3).	Quality of life is recognized as an
57 essential endpoint in the disease management of HIV/AIDS with calls to include good
58 quality of life as a ‘fourth 90’ in the 90-90-90 testing, and treatment targets introduced by the
59 World Health Organization(WHO) in 2016- for who? (4). People with HIV, particularly adolescents,
60 often experience cognitive impairments that have a wide range of negative effects. Even
61 though effective combination antiretroviral therapy (ART) has increased life expectancy for
62 people with HIV, cognitive disorders continue to be a major concern for PLWH and those
63 who provide care for them (5). People with AIDS illnesses experience uncertainty about their
64 prognoses, worse treatment outcomes, poor social relationships, identity crises, and a lower
65 quality of life. They are also confronted with social, and psychological challenges, such as
66 the prospect of financial losses, depression, substance abuse, poor access to quality social
67 support systems, and discrimination (6). Most HIV-infected children are growing into
68 adolescence, and engaging in unprotected sexual relationships which create a public health
69 risk of transmitting a resistant virus strain. By considering this, intensive effort in clinical
70 care, support of stakeholders, and research is urgently needed to sort out the causes, and the
71 gap to support these vulnerable groups (7). there is no focus since this paragraph focuses on people living with HIV not adolescents. Introduction should provide the overview of the problem, the extent and the gaps identified- Scientific writing???

# 73 Materials and methods

## 74 Study area and study period

75 The study was conducted at the Felege Hiwot Comprehensive Specialized
76 Hospital in Bahir-Dar city. Bahir Dar city is the capital of Amhara National Regional
77 State which is 565 km away from Addis Ababa, the capital city of Ethiopia. It is found in
78 Northwestern Ethiopia and is surrounded by the shores of Lake Tana, and the river Abay-Irrelevent
79 or Blue Nile. Based on the report of the city administration plan and service officer, the
80 city has a total population of 422,580. Of whom, 215,516 (51%) were females. In this
81 city administration, there are sixteen public and private ART center health facilities with
82 a total of 13,869 clients taking ART. Of these, 536 were adolescents who had ART
83 follow-up services. Felege Hiwot Comprehensive Specialized Hospital is one of the
84 public health facilities that have an ART clinic. This hospital is a referral hospital that
85 was established during the regime of Emperor Hailesilasie in April 1963. It also provided
86 services for neighboring regions like Benishangul Gumuz and served more than 10
87 million people—relevance????The study was conducted from March 25 to April 30, 2023.

88

## 89 Study design

A phenomenological approach was conducted to explore the lived experience of

90 adolescents living with HIV/AIDS. A phenomenological approach is used to seek to
91 understand the feelings of people and describe the universal essence of a phenomenon by
92 investigating the everyday experiences of human beings (41).

## 93 Study participant recruitment procedure

94 Purposively selected adolescents living with HIV/AIDS who have ART follow-up
95 at Felege Hiwot Comprehensive Specialized Hospital were recruited. Adolescents who
96 knew their HIV status for at least two years, were free from comorbidity, and volunteered
97 for an in-depth interview were included. A brief explanation of the study objective for the
98 ART center focal person, health care workers assigned to the ART clinic, and team
99 leaders of adolescents was given to obtain study participants. After this, the list of
100 adolescents who knew their HIV status more than two years ago and had attitudes
101 towards free talk about themselves was obtained from health professionals and team
102 leaders of the Operation for Triple Zero (OTZ) and psychosocial programs. Consider rephrasing To obtain study participants firstly, the nurse contacts adolescent while they are collecting their
103 drugs, asks them if they are willing to participate in the study, and refers them to the data
104 collector. Secondly, the principal investigator gives detailed information about the study
105 and asks for written consent from family or guardians, and assent from adolescents,-specify, did all adolescents give consent/assent? then
106 conducts interview with those who gave consent and assent until the information gets
107 saturated- consider rephrasing.
108 110

## 109 Sample size determination

110 The sample size was determined by information saturation. Hence based on this
111 approach, thirteen purposefully selected adolescents on ART were included. The
112 information was saturated at the 10th participant, but an additional three participants were
113 interviewed to capture new information if it emerged, and to confirm the saturation point.

## 114 Data collection method and tools

115 An in-depth interview of data collection method was applied using semi-
116 structured open ended interview guide questions. Interview guides was prepared in
117 English, and then translated into the local language (Amharic) for in-depth interviews.
118 Patients’ socio-demographics and the lived experiences of adolescents living with
119 HIV/AIDS were incorporated in the interview guide. Semi-structured open-ended
120 interview guiding questions were used to probe information and the guiding interview
121 question incorporates the questions to answer the objective of the study by using probing
122 methods like how, what, and why questions was applied during data collection time.

## 123 Data collection procedure

124 A quiet and separate room was identified to avoid disturbance during the interview-where?. An audio
125 recorder was turned on during the interview. Field notes were taken during the interview
126 process. All data were collected by the principal investigator with appropriate probing related
127 to the objective of the study.
128 The interview was conducted face-to-face in a quiet room. During the interview, the
129 participants were requested to begin the interview,. Similar to line 124-125 Interviews were conducted until conceptual saturation
130 had been reached (to the point where no further new information was obtained). The probing
131 technique was applied by using how and why to get adequate data on the point of interest.
132 Participants were coded as participants (P): P1, P2, P3, etc., according to their order of
133 interview. The interviewer had taken field notes during the interview –same as lin125regarding body
134 language, verbal and nonverbal cues, and the principal investigator’s reflections on the
135 interview, and the audio was labeled with the identification code. After all, questions have
136 been addressed, and the interviewer has given the participants chance to discuss or mention
137 any additional issues they want to discuss or mention. Finally, the interviewer thanked them
138 after the completion of the interview and made arrangements for the follow-up contact to
139 ensure the transcription was done as the participant said.-consider rephrasing- apply principles of scientific writing

## 140 Operational definition

141 **Adolescence**: Phase of life between child hood and adulthood, from ages of 10 to 19 years (2).
142 **Lived experience**: is an interpretive process situated in an individual’s lifeworld (41).
143 **Guardian**: An individual who provide care for adolescent and have full responsibility for them
144 and may or may not have blood relationship with adolescent

## 145 Trustworthiness

146 To assure trustworthiness a comprehensive field note, audio recording, verbatim
147 transcription, member checking (the data record, transcribed data, and interpretation
148 reviewed by the participant who provided the data), and saturation of data were applied. An
149 external inspection was performed with the initial findings of the data by coauthors and
150 experts to assure the credibility of the findings. An audit trial was done with an intense
151 description of the research beginning from the study until the report writing of the findings to
152 assure dependability, and a thick rich, textual description of the lived experience of the
153 adolescents living with HIV was done to confirm the quality of the data probed. In addition
154 to this, appropriate probing of the data and a thorough description of the result with quotes
155 were applied during data collection and analysis.

## 156 Credibility

157 To achieve credibility of the data, a comprehensive field note, audio recording,
158 verbatim transcription, member checking (the data record, transcribed data, and interpretation
159 reviewed by the participant who provided the data), and saturation of data were applied.
160 Several steps were taken during data coding and analysis like, the development of the data
161 codebook, peer review, and negative case analysis. During the data presentation, quality-
162 enhancement efforts were documented, and a deep description of the research context, the
163 people who had participated in the study, and the experiences and processes observed during
164 the inquiry (a thick description) was employed.

## 165 Dependability

166 Careful documentation, member checking, and an audit trial (a record kept of how
167 this qualitative study was conducted) were used to ensure the dependability of the study.

## 168 Conformability

169 To ensure conformability, raw data was recorded using a smart phone audio recorder
170 during the interview to guarantee conformability, and it was achieved using quotes by linking
171 the words of the participants with the discoveries.

### 172 Transferability

173 To make judgments about transferability, appropriate probes were done to obtain
174 detailed information on responses. The data collector had observed the participants' body
175 movements, facial expressions, eye gaze, and tone of voice during each interview, and
176 everything was recorded (a comprehensive field note was taken). Throughout the study,
177 reflectivity and bracketing methods were employed to minimize respondent bias and the risk
178 of reactivity by holding back the principal investigator's preconceived ideas about the issue
179 under study. After data collection, the principal investigator transcribed the audio-recorded
180 data in the participant’s local language in written form, which was translated to English by a
181 third party, and then the data was coded, categorized, and analyzed based on breadth, depth,
182 context, and nuance for ease of interpretation.- (from line 140-182) unless if the journal requires these- I think is not necessary

## 183 Data management and analysis

184 The ATLAS.ti 8 software was used to facilitate the data analysis. Data collection and
185 analysis were carried out simultaneously. The verbatim transcription of the data was
186 performed daily. The transcribed data were translated conceptually by the principal
187 investigator and language experts. Thematic analysis was used to analyze the data with the
188 implementation of six steps for the analysis process. The principal investigators familiarized
189 themselves with the data by reading the transcribed data repeatedly, initial codes were
190 generated by the principal investigators, generated codes were organized based on their
191 similarity, themes were searched intensively, themes were reviewed, and final themes were
192 named, and defined for thematic analysis. Codes were generated inductively. Similar codes
193 were merged, and irrelevant codes were removed. Categories (sub-themes) emerged from
194 codes that had similar ideas. Themes were created using categories with related concepts.
195 The findings obtained were interpreted according to the data. Quotes were used to highlight
196 each category and show associations with each theme. The principal investigators had a
197 agreed on the final theme analysis. Interpretations of the qualitative data were
198 checked against the verbatim transcripts for accuracy, and consistency of the data.

## 199 Ethical consideration

200 Ethical approval was obtained from the Institutional Review Board of Bahir Dar
201 University College of Medicine and Health Sciences with an ethical approval protocol
202 number of CMHS/IRB/01/757/2023. A permission letter was taken from the Amhara Public
203 Health Institute (APHI) to the study site. Written informed consent and assent were obtained
204 from family or guardians, and adolescents respectively. A briefing on the study objective,
205 benefits, and risks was done for all participants. After explaining the purpose of the study,
206 each participant was asked for permission to record audio. Their name was replaced by a
207 code identification number during data collection, interview, analysis, and reporting time. All
208 research processes adhered to the fundamental ethical principles of autonomy, beneficence,
209 non-maleficence, confidentiality, and the pursuit of justice.

# 210 Result

211 **Table 1**:- Socio-demographic status of the study participants on the experience of living with
212 HIV among adolescents at Felege Hiwot Comprehensive Specialized Hospital, Bahir-Dar,
213 Northwest Ethiopia, 2023

| S.no | Sex | Age | Educational level |
| --- | --- | --- | --- |

| Participant 01 | F | 18 | Grade 12 |
| --- | --- | --- | --- |
| Participant 02 | M | 17 | Grade 1 1 |
| Participant 03 | M | 18 | Grade 9 |
| Participant 04 | M | 18 | Grade 12 |
| Participant 05 | F | 15 | Grade 8 |
| Participant 06 | F | 19 | Grade 12 |
| Participant 07 | F | 18 | Grade 11 |
| Participant 08 | M | 15 | Grade 8 |
| Participant 09 | M | 15 | Grade 8 |
| Participant 10 | F | 16 | Grade 10 |
| Participant 11 | F | 17 | Grade 9 |
| Participant 12 | M | 18 | Grade 10 |
| Participant 13 | M | 19 | Higher educational level |

## 214 Emerged themes

215 After a deep understanding of the data, four main themes and eleven sub-themes
216 emerged from the data as shown below in Table 2. Then the synthesis of the findings
217 was written in each emerged main themes with respective subthemes. Finally, the
218 synthesis of the findings was supported by representative quotes from the participants- data analysis
219 Table 2:- Themes, sub-themes and codes emerged from the data of the experience of living with
220 HIV among adolescent at FHCSH, Northwest Ethiopia, 2023.

| **Main themes** | **Sub themes** | **Codes** |
| --- | --- | --- |
| Lived with burden of HIV/AIDS | Psychological stress | Inferiority, silence, lack of interest, unhappy life, worry/thinking more, talking alone, loss of family,  missing class/school, stereotyped/seen as crazy |
|  | Stigma and discrimination | Isolation, gossips/rumors, talks from other people,  negative attitude of community, fear |
|  | Negative emotional feelings | Sadness, crying, angry, shocked |
| Disclosure | knowing their own HIV status | Told indirectly , know, disclose |
|  | Frustration to disclose their condition to others- better like- disclosure of HIV status to others | Hidden, secret, no comfort to talk freely, careful to talk |
| ART adherence | Habit of medicine intake-meaning???? | Refuse the treatment, stopping the medicine/missing the dose |
|  | Barriers to ART adherence | To many tab, interferes with fasting,  concern of religion, fear of taking in front of others |
|  | Facilitators for ART adherence | Help, remembering them  alarm from peer and family |
|  |  | Hope, hardship in relationship, |

| Future aspiration | Sexual relationship | no freedom to have a friendship |
| --- | --- | --- |
|  | Forming family/being married | Independency  future wish |
|  | Demand of support | Financed, lack of support  suffering for transportation fee, need |

## 221 Theme 1: Lived with burden of HIV/AIDS

222 Adolescents had experienced several burdens in their entire life span due to
223 HIV/AIDS. The adolescent reported that knowing their HIV status resulted in feelings of
224 despair and hopelessness, tied with a sense of forthcoming death. Some of the problems they
225 faced in their life span were psychological challenges related to daily medication intake,
226 anxiety due to fear of stigma and discrimination from society, fear of premature death and
227 sickness, and a lack of freedom in school because talks and gossip from the community and
228 peers about HIV/AIDS disturbs their emotions and makes them live a terrible life. In
229 addition, these adolescents had kept their medicine intake-ART??? a secret and most of them stayed
230 non-adherent to the regimen due to fear of being discovered by others while taking medicine.

### 231 Subtheme 1: Psychological stress

232 Participants described HIV as physically and emotionally hurtful. They have
233 experienced illness, and loss of parents, siblings and family members due to HIV/AIDS.
234 Experiences of the deaths of parents and family members have shown to be a distressing
235 issue for them. They had also experienced psychological stress related to concerns about their
236 deaths.
237 All adolescents understand that the acquisition of the disease is not their fault thus; they have
238 been living an unhappy life due to the mistakes of their family. Most of the participants
239 explained that they are living with the virus as any person does, but they consider themselves
240 different from other people because of their daily intake of medicine, which affects their
241 mindset. Due to this, all of the participants experienced some psychological distress and
242 emotional liabilities in their lives due to being victims of the virus. Most of the respondents
243 feel depressed and uninterested in everything when they think about their HIV status. They
244 were irritated by HIV because this disease affects their minds.
245 For example, a 18 years old female participant explained as**:** *“HIV has its own problems. I*
246 *won't tell you that I am ever happy, because I am not, but I accept it and live by taking*
247 *medicine, because the virus is in my blood”.* (Participant: 07).
248 Participants experienced psychological disturbance due to HIV, which makes them worry
249 more due to fear of isolation, bad talks about HIV from neighbors, classmates, and others.
250 This affects their mood, which leads to despair and hopelessness. Due to this, adolescents
251 with HIV lived with terrible feelings. They have also have emotionally hurt as victims of the
252 virus.
253 This is supported by a 17- year- old female participant explanation as: “*I have felt a terrible*
254 *feeling… I was emotionally hurt.” (*Participant: 01).
255 Some participants explain life with HIV/AIDS as nothing new except taking medicine, and
256 they see themselves as equivalent to other individuals, and they suffer a lot due to their daily
257 intake of medicine and disease condition. This idea is supported by an 18- year-old	male
258 participant as follows; *“No one likes disease …, but even if the disease comes, I am living by*
259 *taking medicine with some stress.” (*Participant: 03).
260 Living with HIV is associated with taking ART for life long and taking medicine
261 through their entire life span makes them live an unhappy life. Adolescents have faced
262 emotional disturbances that lead them to despair in everything. In addition to this, they had
263 faced problems of emotional disturbance like anxiety, worry due to the loss of their family by
264 virus, fear of premature death, and despair in their life span.
265 For example, an 18- year- old female participant said that: “*If I don't take the medicine*
266 *properly, I can become bedridden /sick. This makes me to think more … this disease will kill,*
267 *so I will take medicine properly.” (*Participant: 01.)
268 Adolescents with HIV lived an unpleasant life because talks on topics related to the virus
269 were not comfortable and thoughts coming from society disturbed their emotions. This
270 experience is supported by the idea of an 18- year- old female participant explained as
271 follows:
272 *“I remember that there is always a separate topic of HIV in the biology book in every*
273 *class. When the teacher explains it to us, one of them explains it well and the other one tells*
274 *us to never read what he calls HIV, and he says something that is bad and something that*
275 *will hurt us. There is a problem with teachers, and when I hear that, sometimes I feel a*
276 *psychological problem. I cried because I am a human being, no life will go on apart from*
277 *this.” (*Participant: 07).

### 278 Subtheme 2: Stigma and discrimination

279 Almost all of the participants had suffered from anxiety due to fear of rumors about them,
280 isolation, stigma, and discrimination from their neighbors, classmates, and community at large.
281 According to the report of adolescents, the community has a negative attitude towards the
282 perception of HIV/AIDS because stigmatization of people with HIV still exists.
283 Most participants had not faced any stigma or discrimination from their family or guardians,
284 and their intimate friends, but all of them had experienced isolation and discrimination from
285 the community. A participant describes their experience with stigma and discrimination with
286 very heartbroken speech while manifesting tears in their eyes. For example, an 18- year- old
287 female participant explained as:
288 “*When we were playing games with my neighborhood friends, she has HIV! Why are you*
289 *playing together? That was the only time I felt threatened as a child and there was every*
290 *occasion where I was told that I was not looking at the store to buy something for myself.*
291 *There is a big problem from the community. What can I tell you; it is very difficult! There is a*
292 *lot left.”* (Participant: 07).
293 The attitude of the community towards HIV/AIDS has not changed; they have a negative
294 attitude and adolescents living with HIV/AIDS do not freely interact with society because of
295 their disease condition. Due to this, the adolescents faced different challenges from the
296 neighborhood, classmates, and even teachers. Participants explain that individuals who know
297 their HIV status don’t let them play with them due to fear of HIV, and they talk more about HIV
298 in front of adolescents, and they isolate themselves from their party or team which leads
299 adolescents to face psychological problems and unpleasant feelings. For example, an 18- year-
300 old female participant explained as:
301 *“There is an opportunity for students not to consider a person with HIV as a human being,*
302 *starting from my friend; recently, my friend told me that if I had HIV, I would kill myself.*
303 *Isolation and discrimination from the community have still existed.* (Participant: 07).
304 Many participants didn’t tell others about their HIV status due to fear of stigma and
305 discrimination from the community. This idea is supported by an 18- year- old female participant
306 as follows: “*I do not tell my HIV status for others because there is still stigma and*
307 *discrimination”.* (Participant: 01).
308 The talks and rumors about the virus from the people do not give comfort to the adolescents.
309 People explain HIV in a bad way, and they talk more about stigmatizing the adolescent from
310 their social life. This idea is supported by a 17- year- old male participant as: “*I don't want to*
311 *talk to them because now the society is said to be educated, but in practice it remains, the*
312 *interviews coming from them are not good.” (*Participant: 02).
313 The community had a negative attitude towards HIV and they stigmatize the person with
314 HIV. This is supported by an 18 years old female participant explanation as: *“One of them*
315 *even said that my mother stays away from a person with HIV.”* (Participant: 07).
316 Adolescents reported that they are struggling with gossips or rumors, and unnecessary talks
317 from their classmates about their HIV status and the classmates talk more and stigmatize the
318 child in school. Due to this, they cannot play with them freely and intend to refuse to go to
319 school. Most of the participants do not take ART medicine in front of others due to fear of
320 stigma and discrimination after disclosing their status. For instance, an 18 years old female
321 participant explained as: *“If a guest suddenly comes, I will bring water into my room and*
322 *take it, or I will take it outside. I do what I have to do earlier because there is still stigma and*
323 *discrimination.”* Participant: 01).
324 327
325 328

### 326 Sub theme 3: Negative emotional feelings

327 Participants considered HIV as a negative part of their lives, showing feelings of sadness,
328 fear, and anxiety about their illness. The majorities of the participants were shocked and cried a
329 lot when they heard their status for the first time. Participants faced different kinds of reactions
330 after being informed for the first time later on they faced depression /sadness anxiety, and
331 silence. It made them worry a lot, feel angry, be silent, and stay alone were some of the
332 emotional feelings which most of the participants had experienced. This is supported by a 19-
333 year-old female participant explanation as: *“It was difficult for me because I didn't get used to*
334 *the medicine I had difficulty of sleeping and I would suffer from psychological problem.”*
335 (Participant: 04).
336 Most of the participants were confused about the reason for taking their medication and tried
337 to seek the real information behind the intake of drugs throughout their life span but some of
338 them were told early in childhood period and remained free from such confusion. Some of
339 the participants did not know the drug they were taking for the treatment of the virus and
340 they were told that the medicine is taken for the other disease. Mostly the participants know
341 themselves by their effort. For example, a 19-year-old female participant said that:
342 *“I was in sadness for a long time because of this, I used to cry every day, and then my father*
343 *saw me one day and said, "Why are you crying?" I said to my father, why am I taking this*
344 *medicine? My friend does not take it”.* (Participant: 06).

### 345 Theme 2: Disclosure

346 HIV disclosure has several effects on the life of adolescents living with the human
347 immunodeficiency virus. Emotional disturbance and psychological distress were some of the
348 effects seen following disclosure. The fear of stigma and stigmatization by others made it
349 difficult for adolescents to freely disclose their HIV status to others. Most of the respondents of
350 the study stated that they feared people will get to know their HIV status and spread the news to
351 others, because of this most of them preferred to keep their status to themselves apart from
352 others.

### 353 Subtheme1: Knowing their own HIV status

354 Despite being born with HIV, the participants know about their HIV status during early
355 adolescence. Learning about their HIV status is an emotional and critical point in adolescents’
356 life. Participants reported reacting with sadness, anger, hopelessness, a forthcoming sense of
357 death, and worry about what their HIV diagnosis meant for their future. For instance, a 19-year-
358 old female participant said that: “*I laugh and cry and it's the end of my happiness. Although, it*
359 *made me cry at the time”* (Participant: 06).
360 Participants were confused about the daily intake of medication and they try to seek the real
361 information behind the intake of drugs throughout their life span but some of them were told
362 early in childhood period and remained free from such confusion.
363 This is supported by a 17-year-old male participant's explanation as: *“I started taking*
364 *medicine when I was a child… My family didn't tell me that you are like this. I found out*
365 *through my process.”* (Participant: 2).
366 Some participants reported that their status was explained by their parents. This idea is
367 supported by a 17-year-old female participant as: “*My father told me when I was 11 years*
368 *old”.* (Participant: 11).
369 Participants found themselves that they are living with the virus through their own effort and
370 they got the reason for the daily intake of medicine by their own process*.* For example, a 18
371 years old female participant said that: *“No one told for me, when I came here, there is a poster,*
372 *and everything is there. I see, I read, I always take it from the lesson. This is how I found out."*
373 (Participant: 07).
374 In the process of self-discovery of their HIV status the peer to peer discussion in
375 psychosocial and operation for triple zero program helps them to understand themselves
376 about they are living with the virus. During this discussion health professionals and team
377 leaders of the program told indirectly about the virus. They learn that the medicine is taken
378 daily throughout their life span. Some of participants were not know the drug they were
379 taking for the treatment of virus and they told that the medicine is taken for the other disease.
380 For example, an 18 years old male participant said that: *“I didn't know anything at the time*
381 *and the family was telling me that the medicine was for another disease. When I asked the*
382 *nurse, she told me that clearly and appropriately, and here, the association has registered*
383 *me.”* (Participant: 03).

### 384 Subtheme 2: Frustration to disclose their condition to others

385 Participants don’t want to disclose their status because of fear of stigma and discrimination.
386 Almost all of the participants kept their HIV status a secret from strangers and other persons
387 except their family/guardians, health professionals, and intimate friends. Keeping their status
388 hidden from other individuals due to, fear of isolation, unnecessary talks, and gossip from the
389 community leads adolescents to develop psychological discomfort and low self-esteem.
390 Participants take ART medicine carefully and they never took the medicine freely in front of
391 other people due to fear of accidental disclosure of their status. Participants do not disclose their
392 status for others. Due to this, they are suffering from psychological disturbance following daily
393 intake of medicine and disclosure issue apprehension.
394 For example, a 17 years old male participant said that: “*I don't want to talk to them because*
395 *now the society is said to be educated, but in practice it remains. The interviews coming from*
396 *them are not good.”* (Participant: 02).
397 Participants of the study had concerns about disclosing their status to others, and even if they
398 did, they were very mindful as to whom they should trust such sensitive information. Participants
399 stated that they lived themselves trying to keep as a secret from others. The participants disclose
400 to their family members and they do not worry more about the family. Some participants disclose
401 their HIV status selectively merely to their intimate friends.
402 For example, a 19-year-old male participant said that: *“I didn't tell anyone but one of my*
403 *friends knows my status.” I think that if my classmates know, they will talk about me.”*
404 (Participant: 06).

### 405 Theme 3: ART adherence

406 All of the participants had lived with doubtfulness regarding taking medicine and they had
407 asked themselves and family for what disease the medicine was taken. This question was before
408 knowing of their serostatus and immediately after understanding their status they remain non-
409 adherent to the ART. But after reassurance of themselves and emotional stability, they stay
410 adherent to the ART regimen.
411 414
412 415

### 413 Subtheme 1: Habit of medicine intake

414 Some adolescence intentionally refuse to take medications and their parents find it hard to
415 convince them due to the questions they ask like why I am the only one taking drugs every day?
416 What did I do wrong? As a result, they disengage themselves from treatment leading to poor
417 ART adherence and retention in care before knowing themselves living with HIV.
418 Adolescent practice persistent intake of the drug and remains adherent to the treatment after a
419 full understanding of HIV/AIDs. Psychosocial and operation for triple zero (OTZ) program
420 in the hospital has a great role to engage them to stay adherent to the ART regimen. For
421 example, a 17-year-old female participant explained that: “*I used to take medicine when I feel*
422 *happy, and I leave it when I feel unhappy, but now I have fixed it after joining the OTZ*
423 *program. Now I have started taking medicine regularly since 2015”.* (Participant: 11).
424 In another way, some adolescent refuses medicine intake after disclosure of the serostatus for
425 them because many of them consider that being a victim of HIV is not due to their fault and
426 they pushes the issue to their family. For example, a 19- year- old male participant said that;
427 *“After hearing that I am living with HIV, I stopped taking the medicine. Even though my*
428 *father told me to take it, I couldn't listen; Because of this, I stopped taking medicine for six*
429 *months”. (*Participant: 13).
430 They prefer to stop the medicine and decide to get a cure from their faith and perceive full
431 recovery with the help of God.

### 432 Subtheme 2: Barriers to ART adherence

433 Taking ART was challenging for the participants due to side effects, pill burden, interference
434 with fasting, and daily medication intake schedules. Participants reported that they had
435 experienced suffering from pill burden and side effects of the ART drug. For instance, a 18-
436 year- old male participant said that: *“I was thinking about the amount of medicine I took”*
437 (Participant: 04). Confusion of adolescents behind the intake of medicine makes them stay non-
438 adherent in the treatment. In addition to this being victim of the virus without their fault leads
439 them to refuse treatment and most of the adolescent had experience of stopping medicine due to
440 emotional upset on the way of disease acquisition. Some of the participants had practiced
441 religious healing from the God in who they had faith holding their medicine intake and ignoring
442 the advice of family and health professionals. Some of the participants retain themselves from
443 persistent intake of medicine due to interference of fasting and medication intake time in which
444 taking medicine in the morning is not go with fasting.
445 For example, an 18- year- old female participant said that: “*I may have to eat when I am fasting.*
446 *When someone is fasting, why don't I fast? I suffer a lot.”* (Participant: 07).
447 Participants reported that they had experienced missing the dose several times due to
448 forgetfulness of their medicine when they went to another place. They left their medicine
449 while going to another place. This is supported by an 18- year- old female participant as:
450 *“I went somewhere and when I left I forgot the medicine….I stopped taking medicine”*
451 (participant: 01).

### 452 Subtheme 3: Facilitators for ART adherence

453 In the management of their HIV/AIDs, nearly all ALHIV described that the support of a
454 caregiver and OTZ program has a great influence to stay adherent to the therapy. Some of the
455 participants took medicine regularly and on time without missing the dose and they use the
456 mobile alarm for on-time intake of the medicine. The family/guardians and their intimate friends
457 notify them to take the medicine. Adolescents had informed about the importance of treatment
458 adherence from peer-to-peer discussion and OTZ program service in the hospital. In addition to
459 counseling and advice from health professionals, family, and peers alarms the adolescents to take
460 their medicine on time in every aspect they helped to stay adherent with the ART treatment. For
461 instance, a 19-year-old female participant explained as:
462 “*My colleague said to take me, it's too late, she said to me, the neighbor also called me and told*
463 *me to take the medicine…These help me to take the medicine on time.”* (Participant: 06).
464 Family/guardians' support makes the adolescent to lead their life with hope and had a
465 significant influence to stay adherent to the regimen. For example, a 15-year-old female
466 participant said that: *“My families do everything I ask because they think they can hear what*
467 *they are thinking. Any of my brothers with the virus in their blood will do anything I ask.”*
468 (Participant: 05).
469 The participant reported that the health service provided in the ART clinic for ALHIV is
470 awesome and they are satisfied with the care delivered to them by the staff of the ART clinic.
471 This idea is supported by a 19-year-old female participant as: “*The service provided here is*
472 *very cool and they take care of us like sisters, brothers and mothers in their professions”.*
473 *(*Participant: 06).
474 Most of health professionals provide care appropriately and they got thankful from their
475 clients. For example, a 18- year- old male participant said that: *“To tell the truth, if we say*
476 *that we were raised by professionals rather than our families, it would not be an*
477 *exaggeration. The care given by professionals is very cool.” (*Participant: 03).
478 481

### 479 Theme 4: Future aspirations

480 Most of the participants have hope and aspirations for what they want to be in the future life.
481 Participants have no desire to live with their families and want to lead their life without keeping
482 support from others. The majority of the participants want to get married with a positive spouse
483 and to have children in the future.

### 484 Subtheme 1: Sexual relationship

485 All of the participants were not in sexual relationships, and all of them have never had sexual
486 intercourse, but most of the participants have a plan to have a boy/girlfriend. Participants prefer
487 similar individuals with the virus for their future plans. Some of the participants reported that
488 currently they have no desire to begin sexual intercourse before finishing the class and they plan
489 to begin sexual relationships after accomplishment of education and having their income. This is
490 supported by a 18- year- old male participant as: *“I don't have any sexual relationship… I always*
491 *think that when I grow up.”* (Participant: 04).
492 Participants state that there is no intention to have a sexual partner now and in the future. For
493 example, a 15- year- old female participant explained as: “*God only knows about the future, we*
494 *don't know the terms of tomorrow, so I can't tell you this. I don't worry about it.”*(Participant:

498	05).

499 An 18 year old female participant replied the answer for the question “Do you have a
500 boyfriend/lover?” explained as “*No*; *I don't think about it now because I'm studying.”*
501 (Participant: 07).
502 Some participants have the intention to have partner who are similar serostatus to them in the
503 future life. For example, a 19-year-old female participant said that:
504 “*I have a friend, he is actually here with me, he knows himself and we will take the lesson,*
505 and *we have not started we will have a discussion about HIV transmission and ways of*
506 *transmission; we know because we learn here and go.”*(Participant: 06).

### 507 Subtheme 2: Forming family/being married

508 Most of the participants reported they want HIV positive spouse to marry in the future. This
509 would help them in understanding each other in their lifespan. Participants hope to have a family
510 and children in the future time. Most of them have the intention to form a family after securing
511 their life with income and becoming independent. They report that their family had an interest to
512 have a spouse in the future life and they needed to marry their adolescent with the same
513 individual. For example, a 18- year- old male participant said that: “*I don't have any sexual*
514 *relationship with anything other than class/neighborhood friendship, I always think that when I*
515 *grow up… I will have to start a family.”* (Participant: 04).
516 Some of the participants reported that they were worried to marry the person they want
517 because of their HIV status. For instance, an 18- year- old female participant explained as:
518 “*The creator of the future knows, but even if there is a negative person, I can't handle it. It's*
519 *hard. Even if I have it, I'd prefer it to be a similar person.”(*Participant: 01).
520 Other participants reported that they don’t care about their spouse’s HIV status. If the other
521 person is comfortable with it while knowing his status, they can go on with HIV negative
522 person if the transmission of HIV can be prevented and if the individual accepts marrying
523 HIV positive individual. For example, a 17- year- old male participant explained that: *“I*
524 *don't think there is any problem if she accepts me. If she accepts me, we use condoms during*
525 *intercourse.”*(Participant: 02).

### 526 Subtheme 3: Demand of support

527 Participants reported that they are getting good support from the health professionals working
528 at ART clinics of hospitals and their families/guardians. Most of the participants told that they
529 have a great demand for support from the government, stakeholders, and community to assist
530 them in different perspectives and they want to have job opportunities for themselves to lead
531 their life independently. In addition to this, almost all of them had sought to have better medicine
532 which is taken once or twice a year that cures HIV/AIDS in the future.
533 Participants’ family members and their guardians provide appropriate support for the
534 adolescents and they stand by the side of adolescents in any condition or circumstance. Some
535 participants reported family support is one of the coping strategies which help them while
536 living with HIV/AIDS which helped them to continue with their life, and helped them not to
537 give up in their life. Most of the participants reported that there is a misunderstanding of
538 HIV/AIDS in society because the stigma and discrimination still existed in the community.
539 The participants also stated it would be better if people don’t discriminate HIV positive
540 people. The participant wants support from society from different perspectives and the
541 society should give a great emphasis on their view towards rejection and isolation of people
542 with the virus. Adolescents had faced challenges of isolation from society. Some people
543 don’t consider people with HIV as human and they do not give dignity to people living with
544 HIV. The entire participant reported an intense need from the concerned body to support
545 them with finance, material, and food and to create job opportunities to generate income for
546 themselves. The participant wants to be supplied with different materials from any concerned
547 body. For example, a 18- year- old male participant said that: “*It would be good if the*
548 *relevant body was asked and provide support for us with food and hygiene materials. It is*
549 *good if they support what they can.”* (Participant: 12).
550 Another participant states the need for adolescents to have their own job the concerned body
551 should facilitate the opportunity to have jobs to generate income for them to stay
552 independent. For instance, a 19-year-old female participant explained as:
553 *“It would be great if there were different job opportunities for the youth. That is, if there are*
554 *short courses, it can be educational or professional training.” And if it is supported by the*
555 *budget, it would be great if there is something to motivate a company/organization so that the*
556 *association can act independently.* (Participant: 06).

## 557 Relationship between emerged themes with the experience of living

558 **with HIV among adolescents**
559 There is an interrelationship between emerged themes and sub-themes that shows the lived
560 experience of adolescents with HIV/AIDS. Being a victim of HIV/AIDS has its own problem in
561 the life of human beings. The disease can cause physical, psychological, and social problems
562 among HIV-infected individuals. Therefore, this is harmonious with the emerged theme of “lived
563 with the burden of HIV/AIDS, ART Adherence, disclosure, and future aspiration” in which
564 adolescents had experienced suffering from several problems in their life span. Daily intake of
565 medicine, stigma, discrimination, and psychological distress due to disease makes adolescents
566 lead terrible life. Most adolescents were frustrated and kept their status a secret due to fear of
567 stigma from society which makes them practice non-adherence to the regimen. However, family
568 and social support play an important role in their adherence. Lack of support, stigma, and impact
569 of the disease curtains the life of adolescence and their future aspirations which they had
570 experienced such problems throughout their life span.
571 (Figure 1)

# 572 Discussion

573 The aim of this study was to explore the lived experience of adolescents living with HIV.
574 Psychological, emotional, disclosure issues, ART adherence, and the future aspirations of
575 adolescents living with HIV are major public health problems globally. The findings of this
576 study reveal the lived experiences of adolescents with HIV in four main themes. These lived
577 experiences were grouped under four main themes of lived with the burden of HIV/AIDS,
578 disclosure, ART adherence, and future aspiration with eleven perspective sub-themes.
579 Adolescents had experienced several burdens in their entire life span due to being victims of
580 HIV/AIDS. Some of the problems they had encountered in their life span were challenges
581 related to daily medication intake, psychological stress, and the effects of negative emotions
582 that affect social interactions. In addition, adolescents had experienced missing doses that
583 made them non-adherent to the ART regimen. Fear of disclosure of their HIV status to their
584 friends and neighbors is one of the challenges faced by adolescents living with HIV/AIDS as
585 a result of the stigma and stigmatization by the community. Participants have no desire to
586 live with the family and they have a hope and aspirations to lead their life independently.
587 Participants reported that they have plans and interests to marry a person with HIV to form a
588 family in their life. They want to get married to HIV positive spouse in the future life. The
589 adolescent with HIV kept their status hidden and not shared with other people. This finding is
590 similar to the research done in Brazil and Ghana in which their HIV status was kept as a
591 secret that they don’t share other than family (24, 30). Participants of the study had
592 experienced anxiety about disclosing their status to others, and even if they did, they were
593 very mindful as to whom they should trust such sensitive information. This finding is similar
594 to the study conducted in Ghana among adolescents living with HIV (30). Participants
595 experienced different kinds of negative feelings toward HIV. These feelings are sadness,
596 worry, feelings considering themselves as different from others, and loneliness and they took
597 the disease as a challenge and something that may affect their life negatively. These findings
598 are consistent with the results found in Brazil, South Africa, Tanzania, and Zimbabwe in
599 which negative feelings get superior compared to the positive feelings of adolescents (23, 25,
600 41, and 42). Some participants reported that they can live as a normal person can live.
601 Although they have to take care of themselves to live as healthy individuals and participants
602 believe they are capable of doing what a healthy person can do. There is nothing that blocks
603 them from doing what they want. This finding is similar to the research done in Brazil and
604 South Africa (23, 24). Some participants stopped their medication due to various reasons and
605 they always ask the families why only I took the medicine and not others. After disclosure,
606 the question “why I took the medicine?” was erased from the participant’s life and disclosure
607 helped them to better understand their disease and resulted in increased adherence. This
608 finding is consistent with the research done in Brazil (23). Telling the truth for adolescents
609 has a great clinical significance for them to strengthen their adherence. Most of the
610 participants were informed of their status for the first time by their parents, relatives, health
611 care providers, and friends and others found about it from advertisements about the ART
612 drugs, posters, and how others treated them by themselves. This is a similar finding to the
613 research done in Kenya in which some participants find out about their status by their effort
614 (42). Some participants knew when their parents talked with the health workers about the
615 disease. This finding is similar to the research done in South Africa and Swaziland (24, (43)).
616 The findings revealed that participants reported that learning their HIV status resulted in
617 feelings of despair and hopelessness, coupled with a sense of soon death, loss of interest in
618 families in every setting, and giving up on everything. This finding is similar to the study
619 conducted in Ghana, Tanzania, and Zimbabwe (30, 41, 42). This finding has a clinical
620 advantage that makes health professionals conscious of the early handling of adolescents
621 with such problems. The reactions observed by the participants when they first heard about
622 their status were being shocked, crying, worried, angry, and suicidal feelings. Additionally,
623 they also decreased communication with others, struggled with their family, and feared death
624 was developed which is similar to a study done in Swaziland (44).
625 Adolescence experienced that they can confront their disease as a clinical phenomenon, but
626 they don’t tell others about their HIV status due to fear of different reactions from people.
627 Stigma and discrimination is the main reason they were not disclosed to others. There are
628 different reactions gotten from people which were unnecessary talks/gossip, distancing
629 themselves from them and exposing them to other people. These findings are similar to the
630 research done in South Africa and Swaziland (24, (44). But they don’t hide their status from
631 those who have the disease since understanding with each other would be there. Participants
632 who have told their status to others faced good and bad feedback. A good experience was a
633 friendship becomes stronger after feedback where some received care and they considered
634 their disease as normal. Although breaking up the relationship and distancing were the bad
635 reactions they had experienced in their life. This result was similar to the study done in South
636 Africa (24). There were no participants who had sexual intercourse until now which plays a
637 great role in the prevention of HIV transmission, and most of the participants perceived to
638 have a positive spouse for the future so to understand and help each other and not transmit
639 the virus to others is their reason. They also believed they have to tell their future spouse
640 about their HIV status before marriage. Few of the participants preferred to marry HIV
641 negative spouse in the future which is the opposite finding of studies done in Uganda and
642 Tanzania where the majority of participants preferred to have HIV positive spouse (45, 41).
643 Participants’ perception towards having a similar spouse in the future and practicing
644 abstinence till marriage limits the dissemination of the virus in the community which has a
645 great clinical advantage in control of viral spreading and disease management. In contrast to
646 this the perception of adolescents to marry negative individuals in the future alarms health
647 professionals to give a great focus on this issue. The participants had got satisfactory services
648 in the ART clinics and the care provided by health professional is very fantastic and they are
649 treated as a family. This finding is similar to research done in Brazil and Zambia (23, 42).
650 The psychosocial, operation for triple zero and the tea and coffee program in the ART clinic
651 help them a lot in their life span. They reported it has an impressive effect to motivate and
652 support them in different ways. This program helped them to eliminate the feeling that they
653 are the only ones with the disease in addition to this, they had gotten knowledge of sexual
654 relationships, coping mechanisms of stigmatization, and ART adherence and they freely
655 discuss their plan. It also helps them to have friends and even future spouses. This finding is
656 similar to research done in Swaziland and South Africa (44, 24). The participants reported
657 that they had experienced stigmatization due to HIV from the society and they suggest the
658 continuous provision of education for the society. Family and psychological support are the
659 supports that are helped them to lead their life. This finding is similar to a study done in
660 South Africa (24). Participants were satisfied with the support provided by the government,
661 but they expect more from the government for the innovation of an advanced medication or a
662 cure for HIV to be developed as the science is progressing in the future. They would like it if
663 the government supports them financially too. This need of participants is similar to research
664 done in Uganda (44). Most of participants wanted to build a family and have children in the
665 future, and some of them don’t want to get married in the future and they don’t want to build
666 their own family because their disease might disrupt their sexual life comparable to a
667 research done in South Africa (24). Participants expressed fear of dying prematurely because
668 they expressed fear that they might not be able to accomplish their dreams before death
669 catches up with them which makes them develop psychological problems. This finding is
670 similar to a study conducted in Ghana (30).
671 Generally, the findings of this study reveal several advantages mainly for health care
672 providers who primarily fronted to appreciate the problems of adolescents living with HIV to
673 handle the problem early before experiencing severe problems. Early management of
674 psychological problems, stigma, and discrimination, identifications of the habit of medicine
675 intake, barriers and facilitators for ART adherence, dealing with disclosure issues, and future
676 aspiration has great clinical implications mainly to control and limit the viral dissemination
677 within the community.-does it still fall under the discussion of the reults?

# 678 Limitations of the study

679 The study explores only the adolescents’ view and the study don’t address family/guardians,
680 health care providers, and key informants' views on the lived experience of ALHIV.

# 681 Conclusion

682 This study explored the lived experiences of thirteen adolescents living with HIV/AIDS who
683 have ART follow-up at Felege Hiwot Comprehensive Specialized Hospital. The analysis result
684 of this study was concluded based on the four main themes of “lived with the burden of
685 HIV/AIDS, disclosure, ART adherence, and future aspirations” with perspective eleven sub-
686 themes. Adolescents living with HIV had experienced numerous psychological, emotional and
687 social challenges.	HIV disclosure and its related effects on ALHIV cause an emotional
688 disturbance that makes them face sufferings in their life journey. The fear of stigma and
689 stigmatization by others made it difficult for adolescents to freely disclose their HIV status to
690 others and not to take medicine freely in front of others which affects their ART adherence. The
691 majorities of participants have hopeful future about their life and wants to form their own family
692 in the future. Most of the participants told that they have a great demand for support from the
693 government to have job opportunities for themselves to lead their life independently. Any of the
694 concerned bodies are suggested to resolve the burden of HIV/AIDS with the provision of
695 different support for adolescents, assist them in disclosure, and enhance their ART adherence. In
696 addition to this persistent health education programs for the community to create awareness and
697 change the attitude of society towards stigma and discrimination of people with HIV/AIDS
698 should be implemented.

# 699 Recommendations

700 Different interventions at individual, community, and organizational levels are recommended
701 to responsible bodies to address the problems and health demands of ALHIV.

#### 702 For government and stakeholders

703 It is better to create job opportunities for ALHIV to generate income for themselves to lead
704 their life independently that may decreases the double burden of adolescence living with the
705 virus.
706 It is better to provide a technical support and training for the community targeting the key
707 population of adolescence living with human immunodeficiency virus towards stress coping
708 mechanism and disease prevention.

#### 709 For health professionals working at ART clinic

710 It is better to nurture the counseling service focusing on coping mechanism of psychological
711 problem to relieve them from psychological distress and to stay adherent to the ART
712 regimen.
713 It is better to give persistent health education for the community towards stigma and
714 discrimination.

#### 715 For researcher

716 Further study should incorporate the families, guardians, health care providers, and key
717 informants to triangulate the data on the lived experiences of adolescents living with
718 HIV/AIDS.

# 719 Acknowledgement

720 It is our pleasure to say thanks to Bahir Dar University for ethical approval and giving us
721 ethical clearance to conduct this study. We are also indebted to thank Amhara Public Health
722 Institute for offering us support letter to pursue the study. Last but not the least we would like to
723 extend our gratitude for study participants and staffs of Felege Hiwot Comprehensive
724 Specialized Hospital for their support during the data collection process.

# 725 Author Contributions

726 **Conceptualization**: Bazezew Asfaw Guadie, Yinager Workineh, Minyichil Birhanu Belete,
727 Meseret Mekuriaw Beyene, Tarik Yenew Tessema, Ayalew Kassie Melese
728 **Data curation:** Bazezew Asfaw Guadie, Yinager Workineh, Minyichil Birhanu Belete, Meseret
729 Mekuriaw Beyene, Tarik Yenew Tessema, Ayalew Kassie Melese
730 **Formal analysis:** Bazezew Asfaw Guadie, Yinager Workineh, Minyichil Birhanu Belete,
731 Meseret Mekuriaw Beyene, Tarik Yenew Tessema, Ayalew Kassie Melese
732 **Investigation:** Bazezew Asfaw Guadie, Yinager Workineh, Minyichil Birhanu Belete, Meseret
733 Mekuriaw Beyene, Tarik Yenew Tessema, Ayalew Kassie Melese
734 **Methodology:** Bazezew Asfaw Guadie, Yinager Workineh, Minyichil Birhanu Belete, Meseret
735 Mekuriaw Beyene, Tarik Yenew Tessema, Ayalew Kassie Melese
736 **Project administration:** Bazezew Asfaw Guadie, Yinager Workineh, Minyichil Birhanu Belete,
737 Meseret Mekuriaw Beyene, Tarik Yenew Tessema, Ayalew Kassie Melese
738 **Resources:** Bazezew Asfaw Guadie, Yinager Workineh, Minyichil Birhanu Belete, Meseret
739 Mekuriaw Beyene, Tarik Yenew Tessema, Ayalew Kassie Melese
740 **Supervision:** Yinager Workineh
741 **Validation:** Bazezew Asfaw Guadie, Yinager Workineh, Minyichil Birhanu Belete, Meseret
742 Mekuriaw Beyene, Tarik Yenew Tessema, Ayalew Kassie Melese
743 **Writing original draft:** Bazezew Asfaw Guadie, Yinager Workineh, Minyichil Birhanu Belete
744 **Writing review and editing:** Bazezew Asfaw Guadie, Yinager Workineh, Minyichil Birhanu
745 Belete, Meseret Mekuriaw Beyene, Tarik Yenew Tessema, Ayalew Kassie Melese

# 746 Reference

747 1. Senyurek G, Kavas MV, Ulman YI. Lived experiences of people living with HIV: a descriptive
748 qualitative analysis of their perceptions of themselves, their social spheres, healthcare
749 professionals and the challenges they face daily. BMC Public Health. 2021;21(1):904.
750 2. Sawyer SM, Azzopardi PS, Wickremarathne D, Patton GC. The age of adolescence. The
751 Lancet Child & Adolescent Health. 2018;2(3):223-8.
752 3. Bongfen MC, Torpey K, Ganle J, Ankomah A. Level of adherence and associated factors
753 among HIV-positive adolescents on antiretroviral therapy in Cameroon. African Journal of

754	AIDS Research. 2020;19(4):269-75.

755 4. National Comprehensive HIV Care Guideline 2018.pdf: Federal Minstry of Health; 2018 [cited
756 2023	06/02].	Available	from:	https://[www.afro.who.int/sites/default/files/2019-](http://www.afro.who.int/sites/default/files/2019-04/National%20Comprehensive%20HIV%20Care%20%20Guideline%202018.pdf)
757 [04/National%20Comprehensive%20HIV%20Care%20%20Guideline%202018.pdf](http://www.afro.who.int/sites/default/files/2019-04/National%20Comprehensive%20HIV%20Care%20%20Guideline%202018.pdf).
758 5. Alford K, Daley S, Banerjee S, Vera JH. Quality of life in people living with HIV-associated
759 neurocognitive disorder: A scoping review study. Plos one. 2021;16(5):e0251944.
760 6. Brashers DE, Neidig JL, Goldsmith DJ. Social support and the management of uncertainty for
761 people living with HIV or AIDS. Health communication. 2004;16(3):305-31.
762 7. Haberer J, Mellins C. Pediatric adherence to HIV antiretroviral therapy. Current HIV/AIDS

763	Reports. 2009;6(4):194-200.

764 8. UNICEF. Adolescents Living with HIV:Developing and Strengthening Care and Support
765 Services	2016	[cited	2023	3/2/2023].	Available	from:
766 https://[www.unicef.org/eca/sites/unicef.org.eca/files/2017-](http://www.unicef.org/eca/sites/unicef.org.eca/files/2017-10/Adolescents_Living_with_HIV.pdf)
767 [10/Adolescents_Living_with_HIV.pdf.](http://www.unicef.org/eca/sites/unicef.org.eca/files/2017-10/Adolescents_Living_with_HIV.pdf)
768 9. RETA H. ADHERENCE TO ANTIRETROVIRAL THERAPY AND ASSOCIATED
769 FACTORS AMONG ADULT PEOPLE LIVING WITH HIV/AIDS: THE CASE OF
770 YERGALEM HEALTH CENTER, SOUTHERN, ETHIOPIA: ST. MARY’S UNIVERSITY;

771	2017.

772 10.	Walle BG, Tiruneh CM, Dessie T, Selomon N, Kassaw A, Chekole B, et al. Factors
773 associated with children’s HIV-positive status disclosure in Wolaita Zone, Southern Ethiopia: a
774 cross-sectional study. Italian Journal of Pediatrics. 2022;48(1):1-7.
775 11.	Conway M, Ely A. Adolescents living with HIV: Developing and strengthening care and
776 support services: UNICEF; 2016.
777 12.	Kim S-H, Gerver SM, Fidler S, Ward H. Adherence to antiretroviral therapy in
778 adolescents living with HIV: systematic review and meta-analysis. AIDS (London, England).

779	2014;28(13):1945.

780	13.	Hudelson C, Cluver L. Factors associated with adherence to antiretroviral therapy among

781	adolescents living with HIV/AIDS in low-and middle-income countries: a systematic review.

782	AIDS care. 2015;27(7):805-16.

783 14.	WHO. Global Health Observatory data repository 2022-07-20 [cited 2023 15/02/2023].
784 Available from: https://apps.who.int/gho/data/node.main.622?lang=en.
785 15.	Ajiboye AS, Eshetu F, Lulseged S, Getaneh Y, Tademe N, Kifle T, et al. Predictors of
786 HIV Testing among Youth 15-24 Years in urban Ethiopia, 2017-2018 Ethiopia Population-
787 based HIV Impact Assessment. medRxiv. 2022.
788 16.	Worku ED, Asemahagn MA. Epidemiology of HIV Infection in the Amhara Region of
789 Ethiopia, 2015 to 2018 Surveillance Data Analysis. 2020;12:307-14.
790 17.	Health FMo. National Comprehensive HIV Care Guideline 2018.pdf 2018 [cited 2023
791 06/02].Availablefrom:
792 https://[www.afro.who.int/sites/default/files/201904/National%20Comprehensive%20HIV%20](http://www.afro.who.int/sites/default/files/201904/National%20Comprehensive%20HIV%20)
793 Care%20%20Guideline%202018.pdf.
794 18.	Landry TMM. Navigating life with HIV: The lived experiences of youth living with HIV:
795 The University of Western Ontario (Canada); 2014.
796 19.	Jerene D, Abebe W, Taye K, Ruff A, Hallstrom I. Adolescents living with HIV are at
797 higher risk of death and loss to follow up from care: Analysis of cohort data from eight health
798 facilities in Ethiopia. PloS one. 2019;14(10):e0223655.
799 20.	Galea JT, Wong M, Muñoz M, Valle E, Leon SR, Díaz Perez D, et al. Barriers and
800 facilitators to antiretroviral therapy adherence among Peruvian adolescents living with HIV: a
801 qualitative study. PloS one. 2018;13(2):e0192791.
802 21.	Solomon N, Molla M, Ketema B. “I want to perform and succeed more than those who
803 are HIV-seronegative” Lived experiences of youth who acquired HIV perinetally and attend
804 Zewditu	Memorial	Hospital	ART	clinic,	Addis	Ababa,	Ethiopia.	Plos	one.

805	2021;16(5):e0251848.

806 22.	Brinsdon A, Abel G, Desrosiers J. “I’m taking control”: how people living with
807 HIV/AIDS manage stigma in health interactions. AIDS care. 2017;29(2):185-8.
808 23.	Dybul M, Attoye T, Baptiste S, Cherutich P, Dabis F, Deeks SG, et al. The case for an
809 HIV cure and how to get there. The Lancet HIV. 2021;8(1):e51-e8.
810 24.	Abadía-Barrero CE, Castro A. Experiences of stigma and access to HAART in children
811 and adolescents living with HIV/AIDS in Brazil. Social science & medicine. 2006;62(5):1219-

812	28.

813 25.	Crowley T, van der Merwe A, Skinner D. Adolescent HIV Self-management: Lived
814 Experiences of Adolescents, Caregivers, and Health Care Workers in a South African Context.
815 Journal of the Association of Nurses in AIDS Care. 2019;30(4):e7-e19.
816 26.	women UDMtsoca. HIV and AIDS in adolescents July 2021 [cited 2023 02/19/2023].
817 Available from: https://data.unicef.org/topic/hiv-aids/.
818 27.	and ED, Survey H. HIV/AIDS in Ethiopia 2011 [cited 2023 02/19/2023]. Available from:
819 https://dhsprogram.com/pubs/pdf/DM26/DM26.pdf.
820 28.	Singh A, Jain S. Awareness of HIV/AIDS among school adolescents in Banaskantha
821 district of Gujarat. Health and Population: Perspectives and Issues. 2009;32(2):59-65.
822 29.	Ammon N, Mason S, Corkery JM. Factors impacting antiretroviral therapy adherence
823 among human immunodeficiency virus–positive adolescents in Sub-Saharan Africa: a
824 systematic review. Public Health. 2018;157:20-31.
825 30.	Doat AR, Navab E, Sadat Hoseini AS. Lived experiences of adolescent living with
826 human immunodeficiency virus in Ghana: A phenomenology study. Nursing Open.

827	2021;8(1):299-307.

828 31.	Tosin P. The lived experiences of youths who have lost a parent to AIDS in Wannune,
829 Nigeria: a descriptive phenomenological study 2014.
830 32.	Okonkwoh C. Factors Affecting Antiretroviral Treatment Adherence amongst Young
831 Adults Living with HIV/AIDS in Yaba, Lagos, Nigeria: A Qualitative Study: Master Thesis for
832 Public Health & Society. Available at: [http://www](http://www/) …; 2011.
833 33.	Mesic A, Halim N, MacLeod W, Haker C, Mwansa M, Biemba G. Facilitators and
834 barriers to adherence to antiretroviral therapy and retention in care among adolescents living
835 with HIV/AIDS in Zambia: a mixed methods study. AIDS and Behavior. 2019;23:2618-28.
836 34.	Marques TA. Experiences of Caring for Adolescents Living With HIV.
837 35.	UNICEF. Adolescents Living with HIV: Developing and Strengthening Care and Support
838 Services	2016	[cited	2023	02/20/2023].	Available	from:
839 https://[www.unicef.org/eca/sites/unicef.org.eca/files/2017-](http://www.unicef.org/eca/sites/unicef.org.eca/files/2017-10/Adolescents_Living_with_HIV.pdf)
840 [10/Adolescents_Living_with_HIV.pdf.](http://www.unicef.org/eca/sites/unicef.org.eca/files/2017-10/Adolescents_Living_with_HIV.pdf)
841 36.	Kasande M, Natwijuka A, Katushabe Snr E, Tweheyo Otwine Snr A. Experiences of
842 Caring for Adolescents Living with HIV (ALHIV): A Qualitative Interview with Caregivers.
843 HIV/AIDS-Research and Palliative Care. 2022:577-89.
844 37.	Chory A, Callen G, Nyandiko W, Njoroge T, Ashimosi C, Aluoch J, et al. A pilot study
845 of a mobile intervention to support mental health and adherence among adolescents living with
846 HIV in Western Kenya. AIDS and Behavior. 2022:1-11.
847 38.	Falcão J, Zerbe A, Mellins CA, Mantell J, Brittain K, Kapogiannis B, et al. The secret life
848 of young adolescents living with HIV in northern Mozambique-a mixed methods study. BMC

849	Public Health. 2021;21(1):1-13.

850 39.	Madiba S, Josiah U. Perceived stigma and fear of unintended disclosure are barriers in
851 medication adherence in adolescents with perinatal HIV in Botswana: a qualitative study.
852 BioMed Research International. 2019;2019.
853 40.	Juliet Houghton Country Director CHIVA South Africa[www.chiva-africa.org](http://www.chiva-africa.org/) SHCSC.
854 ART	Adherence	in	Adolescents	2018	[cited	2023	02/20/2023].	Available	from:
855 https://sahivsoc.org/Files/1A_Juliet-Houghton.pdf.
856 41.	Neubauer BE, Witkop CT, Varpio L. How phenomenology can help us learn from the
857 experiences of others. Perspectives on medical education. 2019;8(2):90-7.
858 42.	Abubakar A, Van de Vijver FJ, Fischer R, Hassan AS, K Gona J, Dzombo JT, et al.
859 ‘Everyone has a secret they keep close to their hearts’: challenges faced by adolescents living
860 with HIV infection at the Kenyan coast. BMC public health. 2016;16:1-8.
861 43.	Shabalala FS. Longing for belonging: Adolescents' experiences of living with HIV in
862 different types of families in Swaziland: Universiteit van Amsterdam [Host]; 2017.
863 44.	Kimera E, Vindevogel S, Reynaert D, Justice KM, Rubaihayo J, De Maeyer J, et al.
864 Experiences and effects of HIV-related stigma among youth living with HIV/AIDS in Western
865 Uganda: A photovoice study. PloS one. 2020;15(4):e0232359.
866 866

Figure	[Click here to access/download;Figure;Figure 1.docx](https://www.editorialmanager.com/pone/download.aspx?id=35177494&guid=e9533e79-a28e-4e9c-8274-ce476cc38c93&scheme=1)

#### Lived with burden of HIV/AIDS

- Psychological stress
- Stigma & discrimination
- Negative emotional feelings

#### Disclosure

- Knowing their own HIV status
- Frustration to disclose their condition to others

#### Experience of living with HIV among adolescents

**ART adherence**

- Habit of medicine intake
- Barriers to ART adherence
- Facilitators for ART adherence

#### Future aspirations

- Sexual relationship
- Forming family/being married
- Demand of support

Figure 1:- Relationship of themes and sub themes with the experience of living with HIV among adolescents at Felege Hiwot Comprehensive Specialized Hospital, Bahir-Dar, Northwest Ethiopia, 2023.
