# Supplementary material for: The experience of living with human immunodeficiency virus among adolescents at Felege Hiwot Comprehensive Specialized Hospital Bahir-Dar, Northwest Ethiopia, A phenomenological study
Source: PLoS One. 2025 Jan 9;20(1):e0308347. doi: 10.1371/journal.pone.0308347 (PMC11717265; doi:10.1371/journal.pone.0308347)
Supplement: Attachment — Submitted filename: Response to reviewers for plos one.docx [file pone.0308347.s006.docx]

**Response to reviewers**

Thank you for your constructive feedback and comments, editors and reviewers. We have made the following point-by-point changes to the clean document as recommended by the reviewers. We are grateful and appreciate the reviewers for their positive feedback on our manuscript and recognition of the significance of our research.

| Reviewer | General comments | Comments | Answer/correction | Page number |
| --- | --- | --- | --- | --- |
|  | 1. PLOS ONE format | Write manuscript in PLOS ONE format. | As much as possible our manuscript is written following the format of the PLOS ONE criteria | Whole document |
|  | 2. Data availability | All data should be attached. | Audio records are restricted due to privacy issue. Availability on public may compromise participant privacy but the rest of data related to work of the paper are uploaded as supplementary information. | Avail at supplemntary information |
|  | 3. Tittle on the online submission and on the manuscript should be identical | Amend the tittle on the online submission form or the tittle in the manuscript | Thank you for your comment  It is corrected accordingly | Page 1 |
|  | 4. Reference | The reference should be complete and correct | Thank you for your comment  The extra reference is attached with manuscript from literature review. Thus attached references from literature review part of the paper were removed. | Reference part |
| **Reviewer** #1 | **Questions** | **Comment** | **Answer/correction** | **Page number** |
| **Reviewer** #1 | 1. Abstract: line 17-18 | Para phrasing  /replacing of words needed/ | Thank you very much for your comment  It is corrected accordingly | Page 1 |
| **Reviewer** #1 | 2. Page 8   Abstract line 39 and conclusion part line 696 | What ‘‘persistent health education’’ mean | Thank you very much for your comment, we use this phrase to mean “ongoing, comprehensive health education” and It is corrected accordingly at abstract and conclusion part | Page 2 and |
| **Reviewer** #1 | 3. Page 10 Line 74: | Replace the study area with its qualitative equivalence study setting | Thank you very much for your comment  It is corrected accordingly | Page 4 |
|  | 4. Page 11, line 91-93 | Appropriateness of the design needs further explanation | Thank you very much for your comments  It is corrected accordingly and we try to explain the design briefly. | Page 5 |
|  | 5. Page 11, line 95 | Which type of purposive sampling techniques used/chosen and its appropriateness for the study | Thank you very much for your comments  The random type of purposive sampling technique is used |  |
|  | 6. Page 11, line 99-100 | Who are team leaders of adolescents and how they were appropriate for recruiting study participants | Thank you very much for your comments. The explanation given accordingly  Adolescents on ART have a group and each group has coordinator nominated as leader from them who guides the group for discussion. | 5 |
|  | 7. Page 11   line 100-102 | Reasons of the inclusion cutoff point >2 years for recruiting participants who knew their status | Thank you very much for your comments.  At least two years is taken since most of research done on this issues incorporates adolescents who know their status two years and above. In addition to this, Those adolescents who know their status two years and above recovered and remain free from emotional feelings following knowing their HIV status | 5 |
|  | 8. Page 13   Line 136-137 | Inconsistency with result quotation | Thank you very much for your comments  The relevant quote is chosen for the result | 12-24 |
|  | 9. Page 15-16   Line 186-201 | Elaborate more on the analysis | Thank you very much for your comments  Elaboration of the result is done | 9 |
|  | 10. Page 17-19 | Process of selecting four main themes needs further explanation | Thank you very much for your comments  The process of theme selection explained at result part. | 9 |
|  | 11. Page 18-19   Line | Describe why the sub theme future aspiration is emerged as major theme | Thank you very much for your comments  Future aspiration taken as major theme since the participants think and talk about the future life and they are living under experience of past and current events with thinking for tomorrow. That is why we draft the future aspiration as main theme. | 9 |
|  | 12. Result | Unclear quote | Thank you very much for your comments  Each and every quote written with modification. | 12-24 |
|  | 13. Result | Be more selective about use of examples | Thank you very much for your comments  It is corrected accordingly | 12-24 |
|  | 14. Page 34   Line 557-570 | The information is better to merge with line 218-221 | Thank you very much for your comments  It is corrected accordingly | 11 |
|  | 15. Page 35   Discussion | Make more specific, short and clear for easy understanding | Thank you very much for your comments  It is corrected accordingly |  |
|  | 16. Page 40   Line 696 | What persistent health education mean | Thank you very much for your comments.  We use it to mean “ongoing, comprehensive health education”  It is corrected at the document | 29 |
|  | 17. Page 49   Figure 1 | Show clear clarification on how one main theme is related to another main theme and even how each sub theme in main theme is related other subthemes | Thank you very much for your comments  It is described at topic of “relationship between the main themes and subthemes” part | Page 22-23 |
| **Reviewer #2** | 1. The introduction does not address the topic and does not overview of the problem globally and locally | Address global and local overview | Thank you very much for your comments.  The global and local context of the problem is incorporated and corrected accordingly | Page 2-3 |
|  | 2. Lack of technicality and scientific writing | Lack of scientific writing | Thank you very much for your comments  It is corrected accordingly | The whole document |
|  | 3. Lack of practical recommendation 4. |  | Thank you very much for your comments  It is corrected accordingly | 30 |

Additional explanation on raised questions and comments

1. Who are team leaders of adolescents and how they were appropriate for recruiting study participants?

Answer:

In our study setting the ART clinic has psychosocial program for all adolescents who are taking ART and they are grouped under four team. For each team there is assigned adolescent as a leader of group members. The psychosocial program focuses on discussion about adherence, disclosure issue, operation for triple zero (OTZ), HIV transmission and prevention methods. OTZ focuses on good adherence, good in viral load, and good in follow up. This program targets to make zero high viral load, zero poor adherence and zero missed follow up. Those team leaders are familiar with their group members and they disclose each other. Due to this we use team leader’s inorder to give the list of adolescent who are at least two years and above.
